# Synthesis of Enantiopure Reversed Structured Ether Lipids of the 1-*O*-Alkyl-*sn*-2,3-diacylglycerol Type

**DOI:** 10.3390/md13010173

**Published:** 2015-01-07

**Authors:** Carlos D. Magnusson, Anna V. Gudmundsdottir, Kai-Anders Hansen, Gudmundur G. Haraldsson

**Affiliations:** Science Institute, University of Iceland, Dunhaga 3, 107 Reykjavik, Iceland; E-Mails: carlos_magnusson@hotmail.com (C.D.M.); annavalborg@gmail.com (A.V.G.); Kai-Anders@gmx.de (K.-A.H.)

**Keywords:** diacylglyceryl ethers (DAGE), ether lipids, structured lipids, *n*-3 PUFA, EPA, DHA, *n*-3 PUFA oxime esters, lipase, chemoenzymatic synthesis, focused lipid library

## Abstract

This report describes the synthesis of reversed structured 1-*O*-alkyl-2,3-diacyl-*sn*-glycerols (DAGEs) possessing a pure saturated even number fatty acid (C6:0–C16:0) at the *sn*-2 position along with a pure EPA or DHA located at the terminal *sn*-3 position of the glycerol backbone of chimyl, batyl and selachyl alcohols. These adducts were synthesized by a highly efficient two-step chemoenzymatic process involving an immobilized *Candida antarctica* lipase to introduce pure EPA and DHA activated as oxime esters exclusively to the *sn*-3 terminal position of enantiopure chimyl, batyl and selachyl alcohols in excellent yields. The saturated fatty acids were subsequently incorporated to the remaining *sn*-2 position of the resulting 3-monoacylglyceryl ethers (3-MAGEs) using EDAC coupling agent in the presence of DMAP in very high to excellent yields (85%–98%). No losses of enantiomeric composition were observed during these processes. The multiple utilities of the resulting focused library of reversed structured DAGEs are discussed including how such compounds may possibly be utilized within the pharmaceutical area.

## 1. Introduction

**Figure 1:**
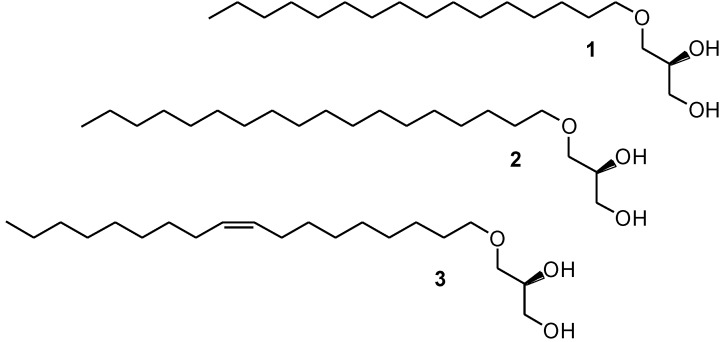
Chemical structures of chimyl (**1**), batyl (**2**) and selachyl (**3**) alcohols.

Nonpolar 1-*O*-alkyl-*sn*-glycerol based ether lipids are major constituents of the liver oils of shark, dogfish and various other species of elasmobranch fish where they occur in their diacylated form as 1-*O*-alkyl-2,3-diacyl-*sn*-glycerols [1,2,3]. They are generally known as diacylglyceryl ethers (DAGEs) and are widely found in the non-polar lipid fractions of aqueous and terrestrial animals and in various tissues in humans, usually as minor lipid components. The three most prevalent hydrocarbon chains present in the alkyl moiety of the 1-*O*-alkyl-*sn*-glycerols present in shark liver oil are C16:0, C18:0 and C18:1 *n*-9, the last one being the most abundant. They correspond to chimyl (**1**), batyl (**2**) and selachyl (**3**) alcohols, respectively, named after the cartilaginous fish species they were isolated from, *i.e.*, chimeras, rays and sharks. Their structures are revealed in Figure 1. As implied by the *sn*-terminology, their natural absolute configuration is *S*.

Shark liver oil has been used for a long time as a therapeutic and preventive agent, for example, in Iceland, Norway and Japan. The ether lipids are precursors to ether phospholipids that participate in structure and functions of the cell membranes. They also bear a strong resemblance to the well-known platelet activating factors [4] and the ether lipids have been claimed to display numerous beneficial effects on human health [1,3,5,6,7]. They include lowering of X-ray therapy induced damages, stimulation for the allergic system and immune control, speeding up the removal of heavy metals from the body as well as displaying anti-tumour and anti-metastasis activities. They have also been claimed to promote adipogenesis [8] and to play a role in regulating embryonic stem cell differentiation [9]. The ether lipids have been exploited as drug-carriers including alkylglycerol based prodrugs [10,11].

The long-chain *n*-3 polyunsaturated fatty acids (*n*-3 PUFAs) are characteristic of fish oil and marine fat. Of these important fatty acids, eicosapentaenoic acid (EPA) and docosahexaenoic acid (DHA) are by far the most prevalent [12,13]. EPA and DHA are associated with various beneficial effects on human health and prevention of various diseases that include inflammation, autoimmune diseases, rheumatoid arthritis, cardiovascular diseases, Alzheimer disease and other neurogenerative diseases, type-2 diabetes and cancer [14,15,16,17,18,19,20,21]. They act in membranes, cell signalling and regulate gene expression via receptors and as precursors to potent lipid mediators. EPA and DHA are indeed precursors to various highly potent eicosanoids and docosanoids that include prostaglandins, leukotrienes, prostacyclins and thromboxanes [16,17] as well as the more recently established highly potent resolvins, protectins and maresins that show potent anti-inflammatory and pro-resolving actions [18,19,20,21]. As such, they may be regarded as prodrugs [22]. Furthermore, they are also available as prescription drugs registered as an adjuvant therapy to treat hypertriglyceridemia both as a mixture of EPA and DHA [23,24] as well as virtually pure EPA devoid of DHA [25,26,27] in the form of ethyl esters.

Structured lipids usually refer to acylglycerol based lipids possessing selected fatty acids located at predetermined positions of the glycerol moiety [28]. Structured triacylglycerols (TAGs) possessing long-chain polyunsaturated bioactive fatty acids such as EPA and DHA at the *sn*-2 position and saturated medium chain (C6:0, C8:0 and C10:0) fatty acids at the terminal *sn*-1,3 positions have gained an increased interest of scientists as a result of their nutritional value and properties [29,30]. In nature, the fatty acids are not randomly distributed in the TAGs that are known to differ significantly in animals and plants from species to species. Classical examples of such structured TAGs include cocoa butter [31], used in chocolate manufacturing, and human milk TAGs [32] and there are multiple reports on stereospecific positioning of fatty acids in animal and plant TAGs [33,34]. Generally, in fish oil TAGs, the mid-position of the glycerol backbone is more enriched with the *n*-3 PUFAs, especially DHA, compared with the terminal positions. It is therefore of interest that in the TAG oil of marine mammals including whale oil and seal oil this is reversed such that the mid-position is to a lower extent enriched with these PUFAs than the outer positions [35]. This difference between fish oil TAGs and marine mammal TAGs has been pointed out by Ackman in relation to the fact that the Greenland Inuits consumed seal fat rather than much fish and had lower incidence of cardiovascular diseases [36,37].

**Figure 2:**
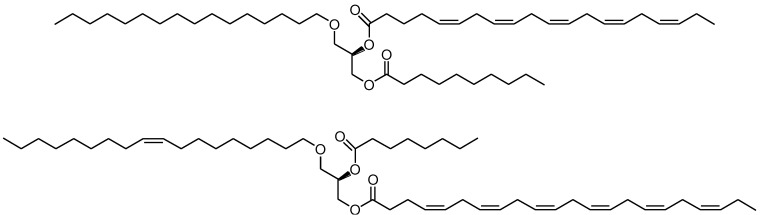
Chemical structures of a normal structured ALM (alkyl-long-medium) type DAGE (diacylglyceryl ethers) of chimyl alcohol possessing EPA at the *sn*-2 position and capric acid (C10:0) at the *sn*-3 position (top) and a reversed structured AML type DAGE of selachyl alcohol possessing DHA at the *sn*-3 position and caprylic acid (C8:0) at the *sn*-2 position (bottom).

We have reported on a highly efficient two-step chemoenzymatic synthesis of MLM (medium-long-medium) type structured TAGs possessing pure EPA and DHA at the mid position with a pure MCFA at the outer positions of the glycerol backbone starting from glycerol [38]. This work has been extended to a focused library of such structured TAGs covering all saturated even carbon number fatty acids from C2–C16:0 located at the terminal positions [38,39]. More recently, this has been further extended to the chemoenzymatic synthesis of similarly structured enantiopure chimyl, batyl and selachyl alcohols possessing pure saturated even carbon number fatty acids (C2–C16:0) located at the terminal *sn*-3 position of the glycerol backbone with pure EPA or DHA at the *sn*-2 position [40]. Such compounds may combine the claimed beneficial health effects of the *n*-3 PUFAs and the ether lipids in one and the same molecule as well as possible beneficial effects of structured lipids possessing the selected fatty acids in predetermined positions of the glycerol moiety. A comprehensive well-defined library of such single pure compounds has been prepared that enables the screening for various important chemical and biological properties including bioactivities. In the current work, the focused library has been expanded to include reversed structured DAGEs, this time possessing the pure EPA and DHA acyl groups at the terminal *sn*-3 position with the saturated fatty acyl groups located at the *sn*-2 position of the glycerol framework. Figure 2 illustrates the chemical structures of a normal ALM (alkyl-long-medium) type structured DAGE (chimyl alcohol possessing EPA and capric acid, C10:0) and a reversed structured DAGE of the AML (alkyl-medium-long) type (selachyl alcohol possessing DHA and caprylic acid, C8:0).

## 2. Results and Discussion

### 2.1. Previous Synthesis of Normal Structured ALM Type DAGE

**Figure 3:**
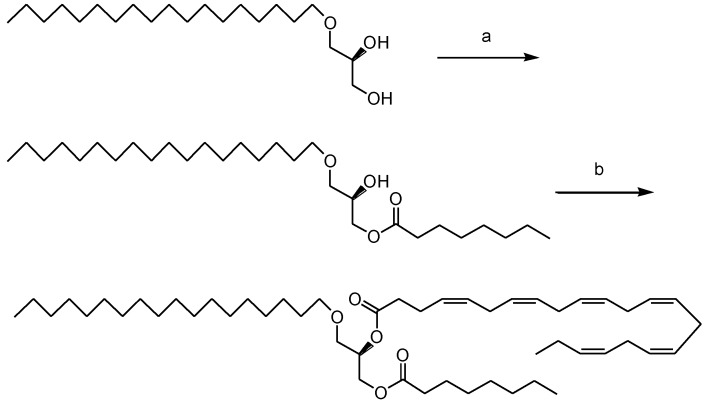
The two-step chemoenzymatic synthesis of normal structured ALM type DAGE shown for batyl alcohol possessing caprylic acid (C8:0) and DHA. Reagents and conditions: (**a**) *C. antarctica* lipase, vinyl octanoate, CH_2_Cl_2_, r.t., 3 h; (**b**) DHA, EDAC, DMAP, CH_2_Cl_2_, r.t., 12 h.

In order to possibly combine in a single molecule the beneficial effects of the MLM type structured TAGs described above, ether lipids of the 1-*O*-alkyl-*sn*-glycerol type, and the long chain *n*-3 PUFAs, a chemoenzymatic synthesis of similarly structured DAGEs possessing pure EPA or DHA at the mid-position and pure MCFA at the end-position, was designed [40]. The chemoenzymatic approach is demonstrated in Figure 3 for batyl alcohol, caprylic acid and DHA. Enantiopure chimyl, batyl and selachyl alcohols obtained from enantiopure (*R*)-solketal (2,3-isopropylidene-*sn*-glycerol) were acylated exclusively into the *sn*-3 position of the glycerol backbone by use of a highly regioselective immobilized *Candida antarctica* lipase (CAL-B from Novozymes) using the saturated fatty acids activated as vinyl esters. The use of vinyl esters secures fast and irreversible reactions under sufficiently mild conditions to eliminate detrimental acyl-migration (*vide supra*) side reactions.

EPA and DHA were subsequently introduced to the remaining *sn*-2 position of the resulting 3-monoacylglyceryl ether (3-MAGE) intermediates by aid of 1-(3-dimethylaminopropyl)-3-ethylcarbo-diimide hydrochloride (EDAC) as a chemical coupling agent in presence of dimethylaminopyridine (DMAP) serving both as a base and catalyst in dichloromethane at r.t. All products and intermediates were obtained enantiopure in excellent yields of excellent chemical and regiopurity.

### 2.2. Synthesis of Reversed Structured AML Type DAGE

**Figure 4:**
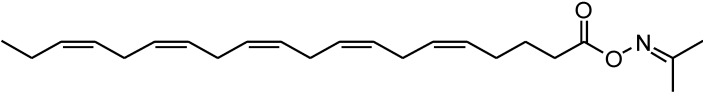
The chemical structure of the EPA acetoxime ester.

The synthesis of the intended reversed structured DAGEs was more of a challenge. That task required an activated form of EPA and DHA suitable for the enzymatic step to take place fast enough and under mild enough conditions to ensure acyl-migration free transformations. This time the vinyl esters were no longer an option since the polyunsaturated fatty acids do not tolerate the chemical conditions required for preparing such derivatives [41]. Furthermore, most of the commercially available microbial lipases do not accept EPA or DHA and their derivatives as substrates. Both obstacles were successfully overcome by use of EPA and DHA activated as oxime esters on which the immobilized *Candida antarctica* lipase (CAL-B) acted at sufficient rate under mild enough condition to eliminate any acyl-migration side-reaction such that the 3-MAGE intermediate adducts were obtained regiopure and in excellent yields (89%–93%). The details have been reported in a recent publication [41]. Figure 4 shows the chemical structure of an acetoxime ester of EPA.

**Figure 5:**
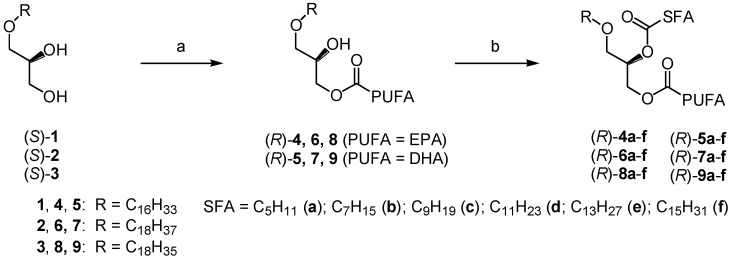
The two-step chemoenzymatic synthesis of ALM type reversed structured DAGEs (for further clarity and details, see Table 1, Table 2 and Table 3). Reagents and conditions: (**a**) *C. antarctica* lipase, PUFA as an acetoxime ester, CH_2_Cl_2_, r.t., 3.5 h; (**b**) SFA, EDAC, DMAP, CH_2_Cl_2_, r.t., 12 h. (For the sake of clarity the use of SFA (saturated fatty acid) and PUFA (polyunsaturated fatty acid) in this figure refers to the hydrocarbon chains of these molecules being saturated or polyunsaturated.)

To obtain the reversed structured DAGEs, the pure saturated fatty acids ranging from C6:0–C16:0 were introduced by chemical coupling to the *sn*-2 position of the resulting 3-MAGEs (*R*)-**4**–**9** derived from chimyl (**1**), batyl (**2**) and selachyl (**3**) alcohols possessing pure EPA and DHA at their *sn*-3 position. The two-step overall chemoenzymatic process is illustrated in Figure 5.

**Table 1:** Reversed structured DAGE products constituting a pure saturated fatty acid (SFA) and EPA (**4a**–**f**) or DHA (**5a**–**f**) for the chimyl alcohol series obtained from the corresponding 3-MAGEs (*R*)-**4** and (*R*)-**5** (see the scheme in Figure 5), their yields and specific optical rotation.

Compound	SFA	PUFA	Yield (%)	[α]_D_
(*R*)-**4a**	-C_5_H_11_	EPA	95	−8.4
(*R*)-**4b**	-C_7_H_15_	EPA	91	−8.6
(*R*)-**4c**	-C_9_H_19_	EPA	90	−8.1
(*R*)-**4d**	-C_11_H_23_	EPA	98	−7.6
(*R*)-**4e**	-C_13_H_27_	EPA	88	−8.3
(*R*)-**4f**	-C_15_H_31_	EPA	97	−7.5
(*R*)-**5a**	-C_5_H_11_	DHA	88	−8.8
(*R*)-**5b**	-C_7_H_15_	DHA	94	−8.6
(*R*)-**5c**	-C_9_H_19_	DHA	85	−8.2
(*R*)-**5d**	-C_11_H_23_	DHA	87	−8.2
(*R*)-**5e**	-C_13_H_27_	DHA	94	−8.0
(*R*)-**5f**	-C_15_H_31_	DHA	96	−8.4

**Table 2:** Reversed structured DAGE products constituting a pure saturated fatty acid (SFA) and EPA (6a-f) or DHA (7a-f) for the batyl alcohol series obtained from the corresponding 3-MAGEs (*R*)-6 and (*R*)-7 (see the scheme in Figure 5), their yields and specific optical rotation.

Compound	SFA	PUFA	Yield (%)	[α]_D_
(*R*)-**6a**	-C_5_H_11_	EPA	90	−8.3
(*R*)-**6b**	-C_7_H_15_	EPA	98	−8.0
(*R*)-**6c**	-C_9_H_19_	EPA	95	−7.9
(*R*)-**6d**	-C_11_H_23_	EPA	94	−7.5
(*R*)-**6e**	-C_13_H_27_	EPA	87	−7.4
(*R*)-**6f**	-C_15_H_31_	EPA	90	−6.6
(*R*)-**7a**	-C_5_H_11_	DHA	94	−8.5
(*R*)-**7b**	-C_7_H_15_	DHA	94	−8.1
(*R*)-**7c**	-C_9_H_19_	DHA	91	−7.9
(*R*)-**7d**	-C_11_H_23_	DHA	88	−7.7
(*R*)-**7e**	-C_13_H_27_	DHA	94	−7.3
(*R*)-**7f**	-C_15_H_31_	DHA	93	−7.2

The coupling reaction was conducted in dichloromethane at room temperature under similar conditions as previously described in the ALM type structured DAGE synthesis [40]. The saturated fatty acids were used in about 10% molar excess with approximately 1.5 molar equivalents of the EDAC coupling agent and 1 molar equivalent of DMAP as based on the starting 3-MAGE adduct. It took the reaction 12 h to proceed to completion. The reversed AML type enantiopure structured DAGE products were obtained chemically and regioisomerically pure in very high to excellent yields (85%–98%) after purification by flash chromatography on short silica gel column. Table 1, Table 2 and Table 3 show the yields obtained for these reversed structured DAGE products along with their specific optical rotation values. Table 1 shows the results for the chimyl derivatives **4a**–**f** for EPA and **5a**–**f** for DHA, Table 2 the batyl derivatives **6a**–**f** (EPA) and **7a**–**f** (DHA) and, finally, Table 3 the selachyl derivatives **8a**–**f** (EPA) and **9a**–**f** (DHA), the total of 36 such reversed structured DAGE derivatives.

**Table 3:** Reversed structured DAGE products constituting a pure saturated fatty acid (SFA) and EPA (**8a**–**f**) or DHA (**9a**–**f**) for the selachyl alcohol series obtained from the corresponding 3-MAGEs (*R*)-**8** and (*R*)-**9** (see the scheme in Figure 5), their yields and specific optical rotation.

Compound	SFA	PUFA	Yield (%)	[α]_D_
(*R*)-**8a**	-C_5_H_11_	EPA	90	−7.7
(*R*)-**8b**	-C_7_H_15_	EPA	86	−7.5
(*R*)-**8c**	-C_9_H_19_	EPA	86	−6.8
(*R*)-**8d**	-C_11_H_23_	EPA	96	−6.8
(*R*)-**8e**	-C_13_H_27_	EPA	90	−6.7
(*R*)-**8f**	-C_15_H_31_	EPA	86	−6.7
(*R*)-**9a**	-C_5_H_11_	DHA	92	−8.3
(*R*)-**9b**	-C_7_H_15_	DHA	89	−7.3
(*R*)-**9c**	-C_9_H_19_	DHA	92	−7.7
(*R*)-**9d**	-C_11_H_23_	DHA	91	−7.6
(*R*)-**9e**	-C_13_H_27_	DHA	92	−8.0
(*R*)-**9f**	-C_15_H_31_	DHA	89	−7.1

It is of interest to notice that the specific optical rotation values obtained for the reversed structured AML type DAGEs are virtually twice as high in magnitude as those reported for the normal structured ALM DAGEs possessing EPA and DHA at the *sn*-2 position with the saturated fatty acids located at the *sn*-3 position of their glycerol framework (negative signs in both cases). Although these values display a narrow range from −6.6 to −8.8, there appears to be trends towards higher rotation when going from chimyl through batyl to the selachyl derivatives, towards lower rotation values when the saturated fatty acids grow in length within each alcohol type and towards higher values for the DHA derivatives as compared to the EPA derivatives.

### 2.3. Regiocontrol by ^1^H and ^13^C NMR Spectroscopy Studies

Similar to what was reported for the chemoenzymatic synthesis of the ALM type structured DAGEs, the ^1^H NMR spectroscopy was of high utility not only to monitor the progress of the reactions and establishing the chemical purity of the intermediates and products, but also to evaluate the extent of possible acyl-migration side reaction and thus the regiopurity and the regiocontrol of these processes. The acyl-migration side reaction is a well-recognized problem when dealing with syntheses involving partially acylated carbohydrates, polyols and glycerol derivatives [38,39,40,42,43]. As before, there were no signs of acyl-migration taking place neither in the enzymatic part [41] of the current synthesis nor the coupling reaction described herein as was firmly established by the ^1^H NMR studies. The details of such NMR studies have been thoroughly described in a previous report [40].

The ^13^C NMR spectroscopy was also of use as a further back-up to the regiocontrol of the syntheses described. This is related to the carbonyl carbons of each of the three categories of fatty acids, namely the saturated fatty acids, EPA and DHA, since it so happens that each of them displays a distinct resonance peak depending upon their location at the *sn*-3 (α) or the *sn*-2 (β) positions of the glycerol moiety. The carbonyl carbons of all the saturated fatty acids C6:0–C16:0 investigated in the current work were observed to resonate at δ 173.12 ppm when located at the *sn*-2 position of the DAGEs. The corresponding resonance value for EPA located at the *sn*-3 position remained at δ 173.15 ppm and that for DHA at that position at δ 172.68 ppm. As can be noticed in the experimental section, these values were quite consistently obtained for these fatty acids in the large majority of cases although some minor deviations were certainly obtained in some cases. This compares to δ 173.41 ppm for the saturated fatty acids when located at the *sn*-3 position and δ 172.84 ppm for EPA and δ 172.37 ppm for DHA when located at the *sn*-2 position as obtained for the ALM type DAGEs in the previous study [40]. Table 4 lists the characteristic ^13^C NMR chemical shift values for these carbonyl groups as based on results from both the normal structured ALM and the reversed structured AML type structured DAGEs.

**Table 4:** The ^13^C NMR chemical shift (δ) values, characteristic of the carbonyl carbons of saturated fatty acids (C6:0–C16:0), EPA and DHA, depending on their location at the *sn*-2 (β) or *sn*-3 (α) positions of the glycerol backbone of the DAGEs.

Fatty Acid Type	δ (ppm), α C=O	δ (ppm), β C=O
C6:0–C16:0	173.41	173.12
EPA	173.15	172.84
DHA	172.68	172.37

### 2.4. Utilization of the Structured DAGE Library

The reversed structured AML type DAGE library currently described may along with the normal structured ALM type DAGE products be utilized in multiple purposes. Such compounds are extremely useful as chemical standards for various analytical purposes including those addressed in the current paper, *i.e.*, the ^1^H and ^13^C NMR spectroscopic details. They may also find use in extensive mass spectrometry studies to investigate how such compounds fractionate in the MS instrument under the conditions used. Such data will most certainly back up possible preparation of structured DAGE compounds intended for human consumption as nutraceuticals produced in more bulky and ‘greener’ processes where the use of organic solvents and reagents is restricted. Such compounds are also useful to investigate biological and physiological effects of individual fatty acids and how such effects may be linked to their positions on the glycerol framework of these compounds. In nature, the vast number of the DAGE molecular species makes such investigations practically impossible.

The structured DAGE and MAGE compounds may also find important use in various biological screenings for bioactivity and activity as potential drugs or prodrugs as well as in drug and prodrug development in the pharmaceutical area. For example, it might be of much interest to link an active drug component to the *sn*-2 or *sn*-3 positions of the DAGEs already possessing EPA or DHA for use as prodrugs. Such combinations of active drugs along with EPA and DHA are well known in the *n*-3 PUFA area where for instance statins have been used in some combinations with EPA and DHA [44]. Alkylglycerols may also find use as drug carriers [10]. There are examples of this taken a step further in terms of covalently linking the *n*-3 PUFAs to a potent drug. An interesting recent example includes the combination of DHA as a tumor targeting molecule to the powerful cytotoxic anticancer agent paclitaxel intended for tumor-targeting drug delivery [45]. There are examples of the 1-*O*-alkyl-*sn*-glycerols being used as prodrugs, where for instance phosphonoformate has been covalently linked to the *sn*-3 position of the glycerol backbone [11].

The 3-MAGE intermediates comprised of an *n*-3 PUFA located at their terminal *sn*-3 position might be conveniently acylated at their *sn*-2 position with a potent drug component to produce prodrugs that combine the benign effects of *n*-3 PUFAs, bioactive alkylglycerols and the pharmaceutical properties of the drug. Alternatively, the drug may be covalently attached to the *sn*-3 position of 1-*O*-alkyl-*sn*-glycerols with a subsequent introduction of the *n*-3 PUFA to the remaining *sn*-2 position of the resulting 3-MAGE. Such prodrugs acquire the biological properties of the *n*-3 PUFA, the ether lipid and the drug in one and the same molecule and might indeed provide improvements to the drug’s therapeutic value.

Obviously, there are numerous interesting options for using the structured DAGEs and their 3-MAGE intermediates in various drug and prodrug formulations that need further investigations. There are also extensive focused libraries of similarly structured MLM and reversely structured LML type triacylglycerols and their 1,3-diacylglycerol (1,3-DAG) intermediates that have been prepared [38,39,41] awaiting further investigations with the aim of their use within the pharmaceutical area. The synthetic methodology applied in the current and previous reports may become of high utility when indroducing potent drugs or other bioactive constituents possessing a carboxyl group to predetermined positions of the glycerol moiety of both TAGs and DAGEs. That methodology is also available for introducing radiolabeled fatty acids to predetermined positions of the glycerol moiety of both structured TAGs and DAGEs for obtaining more information on their fate in their absorption and metabolism to gain better understanding of lipid physiological effects on humans [46].

## 3. Experimental Section

### 3.1. General

^1^H and ^13^C NMR spectra were recorded on a Bruker Avance 400 spectrometer using CDCl_3_ as a solvent. Chemical shifts (δ) are reported in parts per million (ppm) and the coupling constants (*J*) in Hertz (Hz). The following abbreviations are used to decribe the multiplicity: s, singlet; d, doublet; t, triplet; dd, doublet of doublets; m, multiplet. In the assignment parts of the ^1^H and ^13^C NMR spectra, SFA refers to the saturated fatty acyl group. The number of carbon nuclei behind each ^13^C signal is indicated in parentheses after each chemical shift value, when there is more than one carbon responsible for the peak. For all ^13^C NMR peaks, one digit after decimal point is provided except for the carbonyl carbons where two digits after the decimal point are provided to support data expressed in Table 4. All infrared (IR) spectra were conducted on a Nicolet Avatar 360 FT-IR (E.S.P.) Spectrophotometer using neat liquid on a ZnSe plate. The optical activities were measured on an Autopol V from Rudolph Research Analytical, Hackettstown, NJ, USA. Melting points were determined on a Büchi 520 melting point apparatus and are uncorrected. The high-resolution mass spectra (HRMS) were acquired on a Bruker micrOTOF-Q mass spectrometer (Bruker Daltonik GmbH, Bremen, Germany) equipped with an E-spray atmospheric pressure ionization chamber (ESI) ((Bruker Daltonik GmbH, Bremen, Germany). All data analysis was done on a Bruker software ((Bruker Daltonik GmbH, Bremen, Germany).

All chemicals and solvents were used without further purification unless otherwise stated. EDAC (1-(3-dimethylaminopropyl)-3-ethylcarbodiimide hydrochloride) was obtained from Sigma-Aldrich (Steinheim, Germany). Hexanoic acid (98%, zur synthese) and decanoic acid (98%, zur synthese) were obtained from Merck (Darmstadt, Germany) and hexadecanoic acid (99%) from Fluka (Buchs, Switzerland). Octanoic acid (>99.5%), dodecanoic acid (>99.5%), tetradecanoic acid (>99.5%) and 4-dimethylaminopyridine (DMAP, 99%) were obtained from Acros Organics (Geel, Belgium). Dichloromethane and benzene were obtained HPLC grade from Sigma-Aldrich (Steinheim, Germany). Column chromatography was performed on Silica gel 60 (Silicycle, Ontario, CA, USA). Reactions were monitered by TLC on Silica gel 60 F254 (Silicycle, Ontario, CA, USA), with detection by quenching of fluorescence, rhodamine 6G in CH_3_OH and/or with phosphomolybdic acid in ethanol.

#### 3.1.1. Synthesis of 1-*O*-Hexadecyl-2-hexanoyl-3-eicosapentaenoyl-*sn*-glycerol (**4a**)

To a solution of (*R*)-1-*O*-hexadecyl-3-eicosapentaenoyl-*sn*-glycerol **4** (67 mg, 0.112 mmol) and hexanoic acid (15 mg, 0.129 mmol) in CH_2_Cl_2_ (1 mL) were added DMAP (11 mg, 0.090 mmol) and EDAC (32 mg, 0.167 mmol). The resulting solution was stirred at r.t. for 12 h and the solvent then removed under reduced pressure. The residue was then purified by short silica column chromatography (CH_2_Cl_2_) to afford product **4a** (74 mg, 0.106 mmol) as pale yellow oil, yield 95%. ${\mathrm{[\alpha ]}}_{D}^{20}$ −8.4 (*c* 0.91, benzene). ^1^H NMR (400 MHz, CDCl_3_) δ 5.43–5.28 (m, 10H, =C*H*), 5.22–5.17 (m, 1H, CH_2_C*H*CH_2_), 4.34 (dd, 1H, *J =* 11.9 Hz, *J* = 3.7 Hz, C*H*_2_OCO), 4.17 (dd, 1H, *J* = 11.9 Hz, *J* = 6.5 Hz, C*H*_2_OCO), 3.57–3.50 (2xdd, 2H, *J* = 10.6 Hz, *J* = 5.3 Hz, CHC*H*_2_O), 3.47–3.38 (2xdt, 2H, *J* = 9.3 Hz, *J* = 6.6 Hz, OC*H*_2_CH_2_), 2.88–2.76 (m, 8H, =CC*H*_2_C=), 2.32 (2xt, 4H, *J* = 7.6 Hz, *J* = 7.5 Hz, C*H*_2_COO in EPA and SFA), 2.13–2.04 (m, 4H, =CC*H*_2_CH_3_ and =CC*H*_2_CH_2_), 1.70 (quintet (br), 2H, *J* = 7.5 Hz, C*H*_2_CH_2_COO in EPA), 1.66–1.59 (m, 2H, C*H*_2_CH_2_COO in SFA), 1.57–1.50 (quintet (br), 2H, *J* = 7.0 Hz, OCH_2_C*H*_2_), 1.35–1.20 (m, 30H, C*H*_2_), 0.97 (t, 3H, *J* = 7.5 Hz, C*H*_3_ in EPA), 0.89 (t, 3H, *J* = 6.9 Hz, C*H*_3_ in SFA), 0.88 (t, 3H, *J* = 7.0 Hz, C*H*_3_ in ether) ppm (Supplementary Information, Figures S2 and S4–S7); ^13^C NMR (CDCl_3_) δ 173.15 (β, C=O in SFA), 173.12 (α, C=O in EPA), 132.0, 128.9 (2), 128.6, 128.3, 128.2 (2), 128.1, 127.9, 127.0, 71.8, 70.1, 68.9, 62.9, 34.3, 33.5, 31.9, 31.2, 29.7 (7), 29.6 (3), 29.5, 29.4, 26.5, 26.0, 25.6 (2), 25.5, 24.7, 24.6, 22.7, 22.3, 20.6, 14.3, 14.1, 13.9 ppm (Supplementary Information, Figure S3); IR (ZnSe) 3013 (s, CH), 2924 (vs, CH), 2853 (s, CH), 1741 (vs, C=O) cm^−1^; HRMS *m/z* calcd. for C_45_H_78_O_5_ (M + H^+^) 699.5922, found 699.5909.

#### 3.1.2. Synthesis of 1-*O*-Hexadecyl-2-octanoyl-3-eicosapentaenoyl-*sn*-glycerol (**4b**)

The same procedure was followed as described for **4a** using (*R*)-1-*O*-hexadecyl-3-eicosapentaenoyl-*sn*-glycerol **4** (114 mg, 0.190 mmol), octanoic acid (37 mg, 0.257 mmol), DMAP (25 mg, 0.205 mmol) and EDAC (51 mg, 0.266 mmol) in 2 mL CH_2_Cl_2_. The product **4b** (126 mg, 0.173 mmol) was afforded as pale yellow oil, yield 91%. ${\mathrm{[\alpha ]}}_{D}^{20}$ −8.6 (*c* 0.90, benzene). ^1^H NMR (400 MHz, CDCl_3_) δ 5.43–5.28 (m, 10H, =C*H*), 5.22–5.17 (m, 1H, CH_2_C*H*CH_2_), 4.34 (dd, 1H, *J* = 11.9 Hz, *J* = 3.7 Hz, C*H*_2_OCO), 4.17 (dd, 1H, *J* = 11.9 Hz, *J* = 6.5 Hz, C*H*_2_OCO), 3.57–3.50 (2xdd, 2H, *J* = 10.6 Hz, *J* = 5.3 Hz, CHC*H*_2_O), 3.47–3.37 (2xdt, 2H, *J* = 9.3 Hz, *J* = 6.6 Hz, OC*H*_2_CH_2_), 2.88–2.76 (m, 8H, =CC*H*_2_C=), 2.32 (2xt, 4H, *J* = 7.6 Hz, *J* = 7.5 Hz, C*H*_2_COO in EPA and SFA), 2.13–2.04 (m, 4H, =CC*H*_2_CH_3_ and =CC*H*_2_CH_2_), 1.69 (quintet (br), 2H, *J* = 7.5 Hz, C*H*_2_CH_2_COO in EPA), 1.66–1.58 (m, 2H, C*H*_2_CH_2_COO in SFA), 1.57–1.50 (quintet (br), 2H, *J* = 7.0 Hz, OCH_2_C*H*_2_), 1.35–1.20 (m, 34H, C*H*_2_), 0.97 (t, 3H, *J* = 7.5 Hz, C*H*_3_ in EPA), 0.88 (t, 6H, *J* = 6.9 Hz, C*H*_3_) ppm; ^13^C NMR (CDCl_3_) δ 173.15 (β, C=O in SFA), 173.12 (α, C=O in EPA), 132.0, 128.9 (2), 128.6, 128.3, 128.2 (2), 128.1, 127.9, 127.0, 71.8, 70.1, 68.9, 62.9, 34.3, 33.5, 31.9, 31.7, 29.7 (7), 29.6 (3), 29.5, 29.4, 29.0, 28.9, 26.5, 26.0, 25.6 (2), 25.5, 25.0, 24.7, 22.7, 22.6, 20.6, 14.3, 14.1 (2) ppm; IR (ZnSe) 3012 (s, CH), 2922 (vs, CH), 2853 (s, CH), 1740 (vs, C=O) cm^−1^; HRMS *m/z* calcd. for C_47_H_82_O_5_ (M + NH_4_^+^) 744.6501, found 744.6466.

#### 3.1.3. Synthesis of 1-*O*-Hexadecyl-2-decanoyl-3-eicosapentaenoyl-*sn*-glycerol (**4c**)

The same procedure was followed as described for **4a** using (*R*)-1-*O*-hexadecyl-3-eicosapentaenoyl-*sn*-glycerol **4** (126 mg, 0.210 mmol), decanoic acid (35 mg, 0.203 mmol), DMAP (30 mg, 0.245 mmol) and EDAC (68 mg, 0.355 mmol) in 1 mL CH_2_Cl_2_. The product **4c** (142 mg, 0.188 mmol) was afforded as colorless oil, yield 90%. ${\mathrm{[\alpha ]}}_{D}^{20}$ −8.1 (*c* 0.99, benzene). ^1^H NMR (400 MHz, CDCl_3_) δ 5.43–5.28 (m, 10H, =C*H*), 5.22–5.17 (m, 1H, CH_2_C*H*CH_2_), 4.34 (dd, 1H, *J* = 11.9 Hz, *J* = 3.7 Hz, C*H*_2_OCO), 4.17 (dd, 1H, *J* = 11.9 Hz, *J* = 6.5 Hz, C*H*_2_OCO), 3.57–3.50 (2xdd, 2H, *J* = 10.6 Hz, *J* = 5.3 Hz, CHC*H*_2_O), 3.47–3.38 (2xdt, 2H, *J* = 9.3 Hz, *J* = 6.6 Hz, OC*H*_2_CH_2_), 2.88–2.77 (m, 8H, =CC*H*_2_C=), 2.32 (2xt, 4H, *J* = 7.6 Hz, *J* = 7.5 Hz, C*H*_2_COO in EPA and SFA), 2.13–2.04 (m, 4H, =CC*H*_2_CH_3_ and =CC*H*_2_CH_2_), 1.69 (quinted (br), 2H, *J* = 7.5 Hz, C*H*_2_CH_2_COO in EPA), 1.65–1.58 (m, 2H, C*H*_2_CH_2_COO in SFA), 1.57–1.50 (quinted (br), 2H, *J* = 6.8 Hz, OCH_2_C*H*_2_), 1.35–1.20 (m, 38H, C*H*_2_), 0.97 (t, 3H, *J* = 7.5 Hz, C*H*_3_ in EPA), 0.88 (t, 6H, *J* = 6.8 Hz, C*H*_3_) ppm; ^13^C NMR (CDCl_3_) δ 173.15 (β, C=O in SFA), 173.12 (α, C=O in EPA), 132.0, 128.9 (2), 128.6, 128.3, 128.2 (2), 128.1, 127.9, 127.0, 71.8, 70.0, 68.9, 62.9, 34.3, 33.5, 31.9 (2), 29.7 (7), 29.6 (3), 29.5, 29.4 (2), 29.3 (2), 29.1, 26.5, 26.0, 25.6 (2), 25.5, 25.0, 24.7, 22.7 (2), 20.6, 14.3, 14.1 (2) ppm; IR (ZnSe) 3013 (s, CH), 2922 (vs, CH), 2853 (s, CH), 1741 (vs, C=O) cm^−1^; HRMS *m/z* calcd. for C_49_H_86_O_5_ (M + NH_4_^+^) 772.6814, found 772.6781.

#### 3.1.4. Synthesis of 1-*O*-Hexadecyl-2-dodecanoyl-3-eicosapentaenoyl-*sn*-glycerol (**4d**)

The same procedure was followed as for **4a** except using (*R*)-1-*O*-hexadecyl-3-eicosapentaenoyl-*sn*-glycerol **4** (60 mg, 0.100 mmol), dodecanoic acid (24 mg, 0.120 mmol), DMAP (9 mg, 0.070 mmol) and EDAC (28 mg, 0.146 mmol) in 1 mL CH_2_Cl_2_. The product **4d** (77 mg, 0.098 mmol) was afforded as colorless oil, yield 98%. ${\mathrm{[\alpha ]}}_{D}^{20}$ −7.6 (*c* 0.85, benzene). ^1^H NMR (400 MHz, CDCl_3_) δ 5.43–5.28 (m, 10H, =C*H*), 5.22–5.17 (m, 1H, CH_2_C*H*CH_2_), 4.34 (dd, 1H, *J* = 11.9 Hz, *J* = 3.7 Hz, C*H*_2_OCO), 4.17 (dd, 1H, *J* = 11.9 Hz, *J* = 6.5 Hz, C*H*_2_OCO), 3.57–3.50 (2xdd, 2H, *J* = 10.6 Hz, *J* = 5.3 Hz, CHC*H*_2_O), 3.47–3.38 (2xdt, 2H, *J* = 9.3 Hz, *J* = 6.6 Hz, OC*H*_2_CH_2_), 2.88–2.77 (m, 8H, =CC*H*_2_C=), 2.32 (2xt, 4H, *J* = 7.6 Hz, *J* = 7.5 Hz, C*H*_2_COO in EPA and SFA), 2.13–2.04 (m, 4H, =CC*H*_2_CH_3_ and =CC*H*_2_CH_2_), 1.69 (quinted (br), 2H, *J* = 7.5 Hz, C*H*_2_CH_2_COO in EPA), 1.65–1.58 (m, 2H, C*H*_2_CH_2_COO in SFA), 1.58–1.50 (quinted (br), 2H, *J* = 6.8 Hz, OCH_2_C*H*_2_), 1.35–1.20 (m, 42H, C*H*_2_), 0.97 (t, 3H, *J* = 7.5 Hz, C*H*_3_ in EPA), 0.88 (t, 6H, *J* 6.8 Hz, C*H*_3_) ppm; ^13^C NMR (CDCl_3_) δ 173.15 (β, C=O in SFA), 173.12 (α, C=O in EPA), 132.0, 128.9 (2), 128.6, 128.3, 128.2 (2), 128.1, 127.9, 127.0, 71.8, 70.0, 68.9, 62.9, 34.3, 33.5, 31.9 (2), 29.7 (8), 29.6 (4), 29.5 (2), 29.4, 29.3 (2), 29.1, 26.5, 26.0, 25.6 (2), 25.5, 25.0, 24.7, 22.7 (2), 20.6, 14.3, 14.1 (2) ppm; IR (ZnSe) 3013 (s, CH), 2923 (vs, CH), 2853 (s, CH), 1741 (vs, C=O) cm^−1^; HRMS *m/z* calcd. for C_51_H_90_O_5_ (M + H^+^) 783.6861, found 783.6857.

#### 3.1.5. Synthesis of 1-*O*-Hexadecyl-2-tetradecanoyl-3-eicosapentaenoyl-*sn*-glycerol (**4e**)

The same procedure was followed as described for **4a** using (*R*)-1-*O*-hexadecyl-3-eicosapentaenoyl-*sn*-glycerol **4** (50 mg, 0.083 mmol), tetradecanoic acid (23 mg, 0.101 mmol), DMAP (7 mg, 0.057 mmol) and EDAC (23 mg, 0.112 mmol) in 1 mL CH_2_Cl_2_. The product **4e** (59 mg, 0.073 mmol) was afforded as colorless oil, yield 88%. ${\mathrm{[\alpha ]}}_{D}^{20}$ −8.3 (*c* 0.80, benzene). ^1^H NMR (400 MHz, CDCl_3_) δ 5.43–5.28 (m, 10H, =C*H*), 5.22–5.17 (m, 1H, CH_2_C*H*CH_2_), 4.34 (dd, 1H, *J* = 11.9 Hz, *J* = 3.7 Hz, C*H*_2_OCO), 4.17 (dd, 1H, *J* = 11.9 Hz, *J* = 6.5 Hz, C*H*_2_OCO), 3.57–3.50 (2xdd, 2H, *J* = 10.6 Hz, *J* = 5.3 Hz, CHC*H*_2_O), 3.47–3.37 (2xdt, 2H, *J* = 9.3 Hz, *J* = 6.6 Hz, OC*H*_2_CH_2_), 2.88–2.77 (m, 8H, =CC*H*_2_C=), 2.32 (2xt, 4H, *J* = 7.6 Hz, *J* = 7.5 Hz, C*H*_2_COO in EPA and SFA), 2.13–2.04 (m, 4H, =CC*H*_2_CH_3_ and =CC*H*_2_CH_2_), 1.69 (quinted (br), 2H, *J* = 7.5 Hz, C*H*_2_CH_2_COO in EPA), 1.65–1.58 (m, 2H, C*H*_2_CH_2_COO in SFA), 1.58–1.50 (quinted (br), 2H, *J* = 6.8 Hz, OCH_2_C*H*_2_), 1.35–1.20 (m, 46H, C*H*_2_), 0.97 (t, 3H, *J* = 7.5 Hz, C*H*_3_ in EPA), 0.88 (t, 6H, *J* = 6.8 Hz, C*H*_3_) ppm; ^13^C NMR (CDCl_3_) δ 173.15 (β, C=O in SFA), 173.12 (α, C=O in EPA), 132.0, 128.9 (2), 128.6, 128.3, 128.2 (2), 128.1, 127.9, 127.0, 71.8, 70.0, 68.9, 62.9, 34.3, 33.5, 31.9 (2), 29.7 (9), 29.6 (5), 29.5 (2), 29.4 (2), 29.3, 29.1, 26.5, 26.0, 25.6 (2), 25.5, 25.0, 24.7, 22.7 (2), 20.6, 14.3, 14.1 (2) ppm; IR(ZnSe) 3013 (s, CH), 2923 (vs, CH), 2853 (s, CH), 1742 (vs, C=O) cm^−1^; HRMS *m/z* calcd. for C_53_H_94_O_5_ (M + H^+^) 828.7440, found 828.7439.

#### 3.1.6. Synthesis of 1-*O*-Hexadecyl-2-hexadecanoyl-3-eicosapentaenoyl-*sn*-glycerol (**4f**)

The same procedure was followed as described for **4a** using (*R*)-1-*O*-hexadecyl-3-eicosapentaenoyl-*sn*-glycerol **4** (56 mg, 0.093 mmol), hexadecanoic acid (28 mg, 0.109 mmol), DMAP (14 mg, 0.115 mmol) and EDAC (26 mg, 0.136 mmol) in 1 mL CH_2_Cl_2_. The product **4f** (76 mg, 0.091 mmol) was afforded as colorless oil, yield 97%. ${\mathrm{[\alpha ]}}_{D}^{20}$ −7.5 (*c* 1.2, benzene). ^1^H NMR (400 MHz, CDCl_3_) δ 5.43–5.28 (m, 10H, =C*H*), 5.22–5.17 (m, 1H, CH_2_C*H*CH_2_), 4.34 (dd, 1H, *J* = 11.9 Hz, *J* = 3.7 Hz, C*H*_2_OCO), 4.17 (dd, 1H, *J* = 11.9 Hz, *J* = 6.5 Hz, C*H*_2_OCO), 3.57–3.50 (2xdd, 2H, *J* = 10.6 Hz, *J* = 5.3 Hz, CHC*H*_2_O), 3.47–3.38 (2xdt, 2H, *J* = 9.3 Hz, *J* = 6.6 Hz, OC*H*_2_CH_2_), 2.88–2.76 (m, 8H, =CC*H*_2_C=), 2.32 (2xt, 4H, *J* = 7.6 Hz, *J* = 7.5 Hz, C*H*_2_COO in EPA and SFA), 2.13–2.04 (m, 4H, =CC*H*_2_CH_3_ and =CC*H*_2_CH_2_), 1.69 (quinted (br), 2H, *J* = 7.5 Hz, C*H*_2_CH_2_COO in EPA), 1.65–1.58 (m, 2H, C*H*_2_CH_2_COO in SFA), 1.58–1.50 (quinted (br), 2H, *J* = 6.8 Hz, OCH_2_C*H*_2_), 1.35–1.20 (m, 50H, C*H*_2_), 0.97 (t, 3H, *J* = 7.5 Hz, C*H*_3_ in EPA), 0.88 (t, 6H, *J* = 6.8 Hz, C*H*_3_) ppm; ^13^C (CDCl_3_) δ 173.15 (β, C=O in SFA), 173.12 (α, C=O in EPA), 132.0, 128.9 (2), 128.6, 128.3, 128.2 (2), 128.1, 127.9, 127.0, 71.8, 70.0, 68.9, 62.9, 34.3, 33.5, 31.9 (2), 29.7 (11), 29.6 (5), 29.5 (2), 29.4 (2), 29.3, 29.1, 26.5, 26.0, 25.6 (2), 25.5, 25.0, 24.7, 22.7 (2), 20.6, 14.3, 14.1 (2) ppm; IR (ZnSe) 3013 (s, CH), 2922 (vs, CH), 2853 (s, CH), 1742 (vs, C=O) cm^−1^: HRMS *m/z* calcd. for C_55_H_98_O_5_ (M + NH_4_^+^) 856.7753, found 856.7751.

#### 3.1.7. Synthesis of 1-*O*-Hexadecyl-2-hexanoyl-3-docosahexaenoyl-*sn*-glycerol (**5a**)

The same procedure was followed as described for **4a** using (*R*)-1-*O*-hexadecyl-3-docosahexaenoyl-*sn*-glycerol **5** (32 mg, 0.053 mmol), hexanoic acid (8 mg, 0.069 mmol), DMAP (8 mg, 0.069 mmol) and EDAC (16 mg, 0.083 mmol) in 1 mL CH_2_Cl_2_. The product **5a** (34 mg, 0.047 mmol) was afforded as colorless oil, yield 88%. ${\mathrm{[\alpha ]}}_{D}^{20}$ −8.8 (*c* 0.95, benzene). ^1^H NMR (400 MHz, CDCl_3_) δ 5.43–5.28 (m, 12H, =C*H*), 5.22–5.17 (m, 1H, CH_2_C*H*CH_2_), 4.35 (dd, 1H, *J* = 11.9 Hz, *J* = 3.7 Hz, C*H*_2_OCO), 4.18 (dd, 1H, *J* = 11.9 Hz, *J* = 6.5 Hz, C*H*_2_OCO), 3.57–3.50 (2xdd, 2H, *J* = 10.6 Hz, *J* = 5.4 Hz, CHC*H*_2_O), 3.48–3.38 (2xdt, 2H, *J* = 9.3 Hz, *J* = 6.7 Hz, OC*H*_2_CH_2_), 2.88–2.80 (m, 10H, =CC*H*_2_C=), 2.41–2.36 (m, 4H, C*H*_2_C*H*_2_COO in DHA), 2.32 (t, 2H, *J* = 7.5 Hz, C*H*_2_COO in SFA), 2.11–2.04 (m, 2H, =CC*H*_2_CH_3_), 1.66–1.57 (m, 2H, C*H*_2_CH_2_COO in SFA), 1.57–1.50 (m, 2H, OCH_2_C*H*_2_), 1.35–1.21 (m, 30H, C*H*_2_), 0.97 (t, 3H, *J* = 7.5 Hz, C*H*_3_ in DHA), 0.89 (t, 3H, *J* = 6.8 Hz, C*H*_3_ in SFA), 0.88 (t, 3H, *J* = 7.0 Hz, C*H*_3_ in ether); ^13^C NMR (CDCl_3_) δ 173.17 (β, C=O in SFA), 172.72 (α, C=O in DHA), 132.1, 129.4, 128.6, 128.3 (3), 128.2, 128.1 (2), 127.9, 127.8, 127.0, 71.8, 70.1, 69.0, 63.0, 34.3, 34.0, 32.0, 31.3, 29.7 (9), 29.6, 29.5, 29.4, 26.1, 25.7 (2), 25.6 (2), 24.7, 22.7 (2), 22.4, 20.6, 14.3, 14.2, 14.0 ppm; IR (ZnSe) 3013 (s, CH), 2923 (vs, CH), 2853 (s, CH), 1741 (vs, C=O) cm^−1^; HRMS *m/z* calcd. for C_47_H_80_O_5_ (M + H^+^) 725.6079, found 725.6085.

#### 3.1.8. Synthesis of 1-*O*-Hexadecyl-2-octanoyl-3-docosahexaenoyl-*sn*-glycerol (**5b**)

The same procedure was followed as desribed for **4a** using (*R*)-1-*O*-hexadecyl-3-docosahexaenoyl-*sn*-glycerol **5** (65 mg, 0.103 mmol), octanoic acid (21 mg, 0.146 mmol), DMAP (9 mg, 0.072 mmol) and EDAC (30 mg, 0.156 mmol) in 1 mL CH_2_Cl_2_. The product **5b** (73 mg, 0.097 mmol) was afforded as colorless oil, yield 94%. ${\mathrm{[\alpha ]}}_{D}^{20}$ −8.6 (*c* 1.0, benzene). ^1^H NMR (400 MHz, CDCl_3_) δ 5.43–5.28 (m, 12H, =C*H*), 5.22–5.17 (m, 1H, CH_2_C*H*CH_2_), 4.35 (dd, 1H, *J* = 11.9 Hz, *J* = 3.7 Hz, C*H*_2_OCO), 4.18 (dd, 1H, *J* = 11.9 Hz, *J* = 6.5 Hz, C*H*_2_OCO), 3.57–3.50 (2xdd, 2H, *J* = 10.6 Hz, *J* 5.4 Hz, CHC*H*_2_O), 3.49–3.37 (2xdt, 2H, *J* = 9.3, *J* = 6.7 Hz, OC*H*_2_CH_2_), 2.88–2.80 (m, 10H, =CC*H*_2_C=), 2.41–2.35 (m, 4H, C*H*_2_C*H*_2_COO in DHA), 2.32 (t, 2H, *J* = 7.5 Hz, C*H*_2_COO in SFA), 2.11–2.04 (m, 2H, =CC*H*_2_CH_3_), 1.65-1.58 (m, 2H, C*H*_2_CH_2_COO in SFA), 1.58–1.50 (m, 2H, OCH_2_C*H*_2_), 1.35–1.22 (m, 34H, C*H*_2_), 0.97 (t, 3H, *J* = 7.5 Hz, C*H*_3_ in DHA), 0.88 (t, 6H, *J* = 6.8 Hz, C*H*_3_) ppm (Supplementary Information, Figures S8 and S10–S13); ^13^C NMR (CDCl_3_) δ 173.18 (β, C=O in SFA), 172.74 (α, C=O in DHA), 132.1, 129.4, 128.6, 128.3 (3), 128.1 (3), 127.9, 127.8, 127.1, 71.8, 70.1, 69.0, 63.0, 34.4, 34.0, 32.0, 31.7, 29.7 (8), 29.6, 29.5, 29.4, 29.1, 29.0, 26.1, 25.7 (3), 25.6 (2), 25.0, 22.7 (3), 20.6, 14.3, 14.2, 14.1 ppm (Supplementary Information, Figure S9); IR (ZnSe) 3013 (s, CH), 2923 (vs, CH), 2853 (s, CH), 1741 (vs, C=O) cm^−1^; HRMS *m/z* calcd. for C_49_H_84_O_5_ (M + H^+^) 753.6392, found 753.6374.

#### 3.1.9. Synthesis of 1-*O*-Hexadecyl-2-decanoyl-3-docosahexaenoyl-*sn*-glycerol (**5c**)

The same procedure was followed as described for **4a** using (*R*)-1-*O*-hexadecyl-3-docosahexaenoyl-*sn*-glycerol **5** (156 mg, 0.249 mmol), decanoic acid (64 mg, 0.371 mmol), DMAP (35 mg, 0.286 mmol) and EDAC (71 mg, 0.370 mmol) in 1 mL CH_2_Cl_2_. The product **5c** (166 mg, 0.212 mmol) was afforded as colorless oil, yield 85%. ${\mathrm{[\alpha ]}}_{D}^{20}$ −8.2 (*c* 0.97, benzene). ^1^H NMR (400 MHz, CDCl_3_) δ 5.43–5.27 (m, 12H, =C*H*), 5.22–5.17 (m, 1H, CH_2_C*H*CH_2_), 4.35 (dd, 1H, *J* = 11.9 Hz, *J* = 3.7 Hz, C*H*_2_OCO), 4.17 (dd, 1H, *J* = 11.9 Hz, *J* = 6.5 Hz, C*H*_2_OCO), 3.57–3.50 (2xdd, 2H, *J* = 10.6 Hz, *J* = 5.3 Hz, CHC*H*_2_O), 3.47–3.37 (2xdt, 2H, *J* = 9.3 Hz, *J* = 6.6 Hz, OC*H*_2_CH_2_), 2.88–2.80 (m, 10H, =CC*H*_2_C=), 2.41–2.35 (m, 4H, C*H*_2_C*H*_2_COO in DHA), 2.32 (t, 2H, *J* = 7.5 Hz, C*H*_2_COO in SFA), 2.11–2.04 (m, 2H, =CC*H*_2_CH_3_), 1.65–1.58 (m, 2H, C*H*_2_CH_2_COO in SFA), 1.58–1.50 (m, 2H, OCH_2_C*H*_2_), 1.35–1.20 (m, 38H, C*H*_2_), 0.97 (t, 3H, *J* = 7.5 Hz, C*H*_3_ in DHA), 0.88 (t, 6H, *J* = 6.8 Hz, C*H*_3_) ppm; ^13^C NMR (CDCl_3_) δ 173.13 (β, C=O in SFA), 172.68 (α, C=O in DHA), 132.0, 129.3, 128.5, 128.3, 128.2 (2), 128.1, 128.0 (2), 127.8 (2), 127.0, 71.7, 70.0, 68.9, 63.0, 34.3, 34.0, 31.9 (2), 29.7 (7), 29.6 (2), 29.5 (2), 29.4 (2), 29.3 (2), 29.1, 26.0, 25.6 (3), 25.5, 25.0, 22.7 (2), 22.6, 20.5, 14.3, 14.1 (2) ppm; IR (ZnSe) 3013 (s, CH), 2922 (vs, CH), 2853 (s, CH), 1742 (vs, C=O) cm^−1^. HRMS *m/z* calcd. for C_51_H_88_O_5_ (M + NH_4_^+^) 798.6970, found 798.6958.

#### 3.1.10. Synthesis of 1-*O*-Hexadecyl-2-dodecanoyl-3-docosahexaenoyl-*sn*-glycerol (**5d**)

The same procedure was followed as described for **4a** using (*R*)-1-*O*-hexadecyl-3-docosahexaenoyl-*sn*-glycerol **5** (60 mg, 0.095 mmol), dodecanoic acid (23 mg, 0.115 mmol), DMAP (15 mg, 0.123 mmol) and EDAC (27 mg, 0.141 mmol) in 1 mL CH_2_Cl_2_. The product **5d** (67 mg, 0.082 mmol) was afforded as colorless oil, yield 87%. ${\mathrm{[\alpha ]}}_{D}^{20}$ −8.2 (*c* 1.0, benzene). ^1^H NMR (400 MHz, CDCl_3_) δ 5.43–5.28 (m, 12H, =C*H*), 5.22–5.17 (m, 1H, CH_2_C*H*CH_2_), 4.35 (dd, 1H, *J* = 11.9 Hz, *J* = 3.7 Hz, C*H*_2_OCO), 4.17 (dd, 1H, *J* = 11.9 Hz, *J* = 6.5 Hz, C*H*_2_OCO), 3.57–3.50 (2xdd, 2H, *J* = 10.6 Hz, *J* = 5.4 Hz, CHC*H*_2_O), 3.46–3.37 (2xdt, 2H, *J* = 9.3 Hz, *J* = 6.6 Hz, OC*H*_2_CH_2_), 2.88–2.80 (m, 10H, =CC*H*_2_C=), 2.41–2.35 (m, 4H, C*H*_2_C*H*_2_COO in DHA), 2.32 (t, 2H, *J* = 7.5 Hz, C*H*_2_COO in SFA), 2.11–2.04 (m, 2H, =CC*H*_2_CH_3_), 1.65–1.56 (m, 2H, C*H*_2_CH_2_COO in SFA), 1.56–1.50 (m, 2H, OCH_2_C*H*_2_), 1.35–1.21 (m, 42H, C*H*_2_), 0.97 (t, 3H, *J* = 7.5 Hz, C*H*_3_ in DHA), 0.88 (t, 6H, *J* = 6.8 Hz, C*H*_3_) ppm; ^13^C NMR (CDCl_3_) δ 173.13 (β, C=O in SFA), 172.68 (α, C=O in DHA), 132.0, 129.4, 128.6, 128.3 (2), 128.2, 128.1 (2), 128.0, 127.9, 127.8, 127.0, 71.8, 70.1, 69.0, 63.0, 34.4, 34.0, 31.9 (2), 29.7 (7), 29.6 (5), 29.5 (2), 29.4, 29.3 (2), 29.1, 26.0, 25.6 (3), 25.5, 25.0, 22.7 (3), 20.6, 14.3, 14.1 (2) ppm; IR (ZnSe) 3014 (s, CH), 2922 (vs, CH), 2853 (s, CH), 1742 (vs, C=O) cm^−1^; HRMS *m/z* calcd. for C_53_H_92_O_5_ (M + H^+^) 809.7018, found 809.7016.

#### 3.1.11. Synthesis of 1-*O*-Hexadecyl-2-tetradecanoyl-3-docosahexaenoyl-*sn*-glycerol (**5e**)

The same procedure was followed as described for **4a** using (*R*)-1-*O*-hexadecyl-3-docosahexaenoyl-*sn*-glycerol **5** (148 mg, 0.236 mmol), tetradecanoic acid (60 mg, 0.263 mmol), DMAP (31 mg, 0.254 mmol) and EDAC (67 mg, 0.349 mmol) in 2 mL CH_2_Cl_2_. The product **5e** (185 mg, 0.221 mmol) was afforded as colorless oil, yield 94%. ${\mathrm{[\alpha ]}}_{D}^{20}$ −8.0 (*c* 1.0, benzene). ^1^H NMR (400 MHz, CDCl_3_) δ 5.43–5.28 (m, 12H, =C*H*), 5.22–5.17 (m, 1H, CH_2_C*H*CH_2_), 4.35 (dd, 1H, *J* = 11.9 Hz, *J* = 3.7 Hz, C*H*_2_OCO), 4.17 (dd, 1H, *J* = 11.9 Hz, *J* = 6.5 Hz, C*H*_2_OCO), 3.57–3.50 (2xdd, 2H, *J* = 10.6 Hz, *J* 5.3 Hz, CHC*H*_2_O), 3.46–3.37 (2xdt, 2H, *J* = 9.3 Hz, *J* = 6.6 Hz, OC*H*_2_CH_2_), 2.88–2.80 (m, 10H, =CC*H*_2_C=), 2.41-2.35 (m, 4H, C*H*_2_C*H*_2_COO in DHA), 2.32 (t, 2H, *J* = 7.5 Hz, C*H*_2_COO in SFA), 2.11–2.04 (m, 2H, =CC*H*_2_CH_3_), 1.65–1.58 (m, 2H, C*H*_2_CH_2_COO in SFA), 1.58–1.50 (m, 2H, OCH_2_C*H*_2_), 1.35–1.20 (m, 46H, C*H*_2_), 0.97 (t, 3H, *J* = 7.5 Hz, C*H*_3_ in DHA), 0.88 (t, 6H, *J* = 6.8 Hz, C*H*_3_) ppm; ^13^C NMR (CDCl_3_) δ 173.15 (β, C=O in SFA), 172.70 (α, C=O in DHA), 132.0, 129.3, 128.5, 128.3, 128.2 (2), 128.1 (2), 128.0, 127.8 (2), 127.0, 71.7, 70.0, 68.9, 63.0, 34.3, 34.0, 31.9 (2), 29.7 (11), 29.6 (2), 29.5 (3), 29.4 (2), 29.3, 29.1, 26.0, 25.6 (3), 25.5, 25.0, 22.7 (2), 22.6, 20.6, 14.3, 14.1 (2) ppm; IR (ZnSe) 3013 (s, CH), 2922 (vs, CH), 2853 (s, CH), 1741 (vs, C=O) cm^−1^; HRMS *m/z* calcd. for C_55_H_96_O_5_ (M + H^+^) 837.7331, found 837.7315.

#### 3.1.12. Synthesis of 1-*O*-Hexadecyl-2-hexadecanoyl-3-docosahexaenoyl-*sn*-glycerol (**5f**)

The same procedure was followed as described for **4a** using (*R*)-1-*O*-hexadecyl-3-docosahexaenoyl-*sn*-glycerol **5** (103 mg, 0.164 mmol), hexadecanoic acid (49 mg, 0.191 mmol), DMAP (21 mg, 0.172 mmol) and EDAC (47 mg, 0.245 mmol) in 1 mL CH_2_Cl_2_. The product **5f** (139 mg, 0.161 mmol) was afforded as colorless oil, yield 96%. ${\mathrm{[\alpha ]}}_{D}^{20}$ −8.4 (*c* 0.89, benzene). ^1^H NMR (400 MHz, CDCl_3_) δ 5.43–5.28 (m, 12H, =C*H*), 5.22–5.17 (m, 1H, CH_2_C*H*CH_2_), 4.35 (dd, 1H, *J* = 11.9 Hz, *J* = 3.7 Hz, C*H*_2_OCO), 4.17 (dd, 1H, *J* = 11.9 Hz, *J* = 6.5 Hz, C*H*_2_OCO), 3.57–3.50 (2xdd, 2H, *J* = 10.6 Hz, *J* = 5.3 Hz, CHC*H*_2_O), 3.46–3.37 (2xdt, 2H, *J* = 9.3 Hz, *J* = 6.6 Hz, OC*H*_2_CH_2_), 2.88–2.80 (m, 10H, =CC*H*_2_C=), 2.41–2.35 (m, 4H, C*H*_2_C*H*_2_COO in DHA), 2.32 (t, 2H, *J* = 7.5 Hz, C*H*_2_COO in SFA), 2.11–2.04 (m, 2H, =CC*H*_2_CH_3_), 1.65–1.58 (m, 2H, C*H*_2_CH_2_COO in SFA), 1.58–1.50 (m, 2H, OCH_2_C*H*_2_), 1.35–1.21 (m, 50H, C*H*_2_), 0.97 (t, 3H, *J* = 7.5 Hz, C*H*_3_ in DHA), 0.88 (t, 6H, *J* = 6.8 Hz, C*H*_3_) ppm; ^13^C NMR (CDCl_3_) δ 173.13 (β, C=O in SFA), 172.68 (α, C=O in DHA), 132.0, 129.3, 128.6, 128.3 (2), 128.2, 128.1 (2), 128.0, 127.9, 127.8, 127.0, 71.7, 70.0, 69.0, 63.0, 34.4, 34.0, 31.9 (2), 29.7 (12), 29.6 (4), 29.5 (2), 29.4 (2), 29.3, 29.1, 26.0, 25.6 (3), 25.5, 25.0, 22.7 (3), 20.6, 14.3, 14.1 (2) ppm; IR (ZnSe) 3013 (s, CH), 2921 (vs, CH), 2852 (s, CH), 1742 (vs, C=O) cm^−1^; HRMS *m/z* calcd. for C_57_H_100_O_5_ (M + NH_4_^+^) 882.7909, found 882.7888.

#### 3.1.13. Synthesis of 1-*O*-Octadecyl-2-hexanoyl-3-eicosapentaenoyl-*sn*-glycerol (**6a**)

The same procedure was followed as described for **4a** using (*R*)-1-*O*-octadecyl-3-eicosapentaenoyl-*sn*-glycerol **6** (115 mg, 0.183 mmol), hexanoic acid (21 mg, 0.181 mmol), DMAP (23 mg, 0.188 mmol) and EDAC (50 mg, 0.261 mmol) in 1 mL CH_2_Cl_2_. The product **6a** (119 mg, 0.164 mmol) was afforded as colorless oil, yield 90%. ${\mathrm{[\alpha ]}}_{D}^{20}$ −8.3 (*c* 1.0, benzene). ^1^H NMR (400 MHz, CDCl_3_) δ 5.43–5.28 (m, 10H, =C*H*), 5.22–5.17 (m, 1H, CH_2_C*H*CH_2_), 4.34 (dd, 1H, *J* = 11.9 Hz, *J* = 3.7 Hz, C*H*_2_OCO), 4.17 (dd, 1H, *J* = 11.9, *J* 6.5 Hz, C*H*_2_OCO), 3.57–3.50 (2xdd, 2H, *J* = 10.6 Hz, *J* = 5.3 Hz, CHC*H*_2_O), 3.47–3.38 (2xdt, 2H, *J* = 9.3 Hz, *J* = 6.6 Hz, OC*H*_2_CH_2_), 2.88–2.79 (m, 8H, =CC*H*_2_C=), 2.32 (2xt, 4H, *J* = 7.6 Hz, *J* = 7.5 Hz, C*H*_2_COO in EPA and SFA), 2.13–2.04 (m, 4H, =CC*H*_2_CH_3_ and =CC*H*_2_CH_2_), 1.70 (quinted (br), 2H, *J* = 7.5 Hz, C*H*_2_CH_2_COO in EPA), 1.66–1.59 (m, 2H, C*H*_2_CH_2_COO in SFA), 1.57–1.50 (quinted (br), 2H, *J* = 6.8 Hz, OCH_2_C*H*_2_), 1.35–1.25 (m, 34H, C*H*_2_), 0.97 (t, 3H, *J* = 7.5 Hz, C*H*_3_ in EPA), 0.89 (t, 3H, *J* = 6.9 Hz, C*H*_3_ in SFA), 0.88 (t, 3H, *J* = 7.0 Hz, C*H*_3_ in ether) ppm; ^13^C NMR (CDCl_3_) δ 173.15 (β, C=O in SFA), 173.12 (α, C=O in EPA), 132.0, 128.9 (2), 128.6, 128.3, 128.2 (2), 128.1, 127.9, 127.0, 71.8, 70.1, 68.9, 62.9, 34.3, 33.5, 31.9, 31.2, 29.7 (8), 29.6 (3), 29.5, 29.4, 26.5, 26.0, 25.6 (3), 25.5, 24.7, 24.6, 22.7, 22.3, 20.6, 14.3, 14.1, 13.9 ppm; IR (ZnSe) 3013 (s, CH), 2921 (vs, CH), 2851 (s, CH), 1741 (vs, C=O) cm^−1^; HRMS *m/z* calcd. for C_47_H_82_O_5_ (M + NH_4_^+^) 744.6501, found 744.6481.

#### 3.1.14. Synthesis of 1-*O*-Octadecyl-2-octanoyl-3-eicosapentaenoyl-*sn*-glycerol (**6b**)

The same procedure was followed as described for **4a** using (*R*)-1-*O*-octadecyl-3-eicosapentaenoyl-*sn*-glycerol **6** (65 mg, 0.103 mmol), octanoic acid (20 mg, 0.139 mmol), DMAP (13 mg, 0.107 mmol) and EDAC (33 mg, 0.172 mmol) in 1 mL CH_2_Cl_2_. The product **6b** (76 mg, 0.101 mmol) was afforded as pale yellow oil, yield 98%. ${\mathrm{[\alpha ]}}_{D}^{20}$ −8.0 (*c* 0.83, benzene). ^1^H NMR (400 MHz, CDCl_3_) δ 5.43–5.28 (m, 10H, =C*H*), 5.22–5.17 (m, 1H, CH_2_C*H*CH_2_), 4.34 (dd, 1H, *J* = 11.9 Hz, *J* = 3.7 Hz, C*H*_2_OCO), 4.17 (dd, 1H, *J* = 11.9 Hz, *J* = 6.5 Hz, C*H*_2_OCO), 3.57–3.50 (2xdd, 2H, *J* = 10.6 Hz, *J* 5.3 Hz, CHC*H*_2_O), 3.47–3.38 (2xdt, 2H, *J* = 9.3 Hz, *J* = 6.6 Hz, OC*H*_2_CH_2_), 2.88–2.77 (m, 8H, =CC*H*_2_C=), 2.32 (2xt, 4H, *J* = 7.5 Hz, *J* = 7.5 Hz, C*H*_2_COO in EPA and SFA), 2.13–2.04 (m, 4H, =CC*H*_2_CH_3_ and =CC*H*_2_CH_2_), 1.69 (quintet (br), 2H, *J* = 7.5 Hz, C*H*_2_CH_2_COO in EPA), 1.66–1.58 (m, 2H, C*H*_2_CH_2_COO in SFA), 1.57–1.50 (quintet (br), 2H, *J* = 6.8 Hz, OCH_2_C*H*_2_), 1.35–1.20 (m, 38H, C*H*_2_), 0.97 (t, 3H, *J* = 7.5 Hz, C*H*_3_ in EPA), 0.88 (t, 6H, *J* = 6.9 Hz, C*H*_3_) ppm; ^13^C NMR (CDCl_3_) δ 173.15 (β, C=O in SFA), 173.12 (α, C=O in EPA), 132.0, 128.9 (2), 128.6, 128.3, 128.2 (2), 128.1, 127.9, 127.0, 71.8, 70.0, 68.9, 62.9, 34.3, 33.5, 31.9, 31.7, 29.7 (9), 29.6 (2), 29.5, 29.4, 29.0, 28.9, 26.5, 26.0, 25.6 (2), 25.5, 25.0, 24.6, 24.7, 22.7, 22.6, 20.6, 14.3, 14.1 (2) ppm; IR (ZnSe) 3013 (s, CH), 2923 (vs, CH), 2853 (s, CH), 1741 (vs, C=O) cm^−1^; HRMS *m/z* calcd. for C_49_H_86_O_5_ (M + NH_4_^+^) 772.6800, found 772.6790.

#### 3.1.15. Synthesis of 1-*O*-Octadecyl-2-decanoyl-3-eicosapentaenoyl-*sn*-glycerol (**6c**)

The same procedure was followed as described for **4a** using (*R*)-1-*O*-octadecyl-3-eicosapentaenoyl-*sn*-glycerol **6** (82 mg, 0.130 mmol), decanoic acid (26 mg, 0.151 mmol), DMAP (20 mg, 0.163 mmol) and EDAC (41 mg, 0.214 mmol) in 1 mL CH_2_Cl_2_. The product **6c** (97 mg, 0.124 mmol) was afforded as pale yellow oil, yield 95%. ${\mathrm{[\alpha ]}}_{D}^{20}$ −7.9 (*c* 0.82, benzene). ^1^H NMR (400 MHz, CDCl_3_) δ 5.43–5.28 (m, 10H, =C*H*), 5.22–5.17 (m, 1H, CH_2_C*H*CH_2_), 4.34 (dd, 1H, *J* = 11.9 Hz, *J* = 3.7 Hz, C*H*_2_OCO), 4.17 (dd, 1H, *J* = 11.9 Hz, *J* = 6.5 Hz, C*H*_2_OCO), 3.57–3.50 (2xdd, 2H, *J* = 10.7 Hz, *J* = 5.4 Hz, CHC*H*_2_O), 3.47–3.38 (2xdt, 2H, *J* = 9.3 Hz, *J* = 6.7 Hz, OC*H*_2_CH_2_), 2.86–2.79 (m, 8H, =CC*H*_2_C=), 2.32 (2xt, 4H, *J* = 7.6, Hz *J* = 7.5 Hz, C*H*_2_COO in EPA and SFA), 2.13–2.04 (m, 4H, =CC*H*_2_CH_3_ and =CC*H*_2_CH_2_), 1.69 (quintet (br), 2H, *J* = 7.5 Hz, C*H*_2_CH_2_COO in EPA), 1.65–1.58 (m, 2H, C*H*_2_CH_2_COO in SFA), 1.58–1.50 (m, 2H, OCH_2_C*H*_2_), 1.38–1.21 (m, 42H, C*H*_2_), 0.97 (t, 3H, *J* = 7.5 Hz, C*H*_3_ in EPA), 0.88 (t, 6H, *J* = 6.8 Hz, C*H*_3_) ppm (Supplementary Information, Figure S14); ^13^C NMR (CDCl_3_) δ 173.14 (β, C=O in SFA), 173.12 (α, C=O in EPA), 132.0, 128.9 (2), 128.6, 128.3, 128.2 (2), 128.1, 127.9, 127.0, 71.8, 70.1, 69.0, 62.9, 34.4, 33.5, 31.9 (2), 29.7 (11), 29.6 (2), 29.5, 29.4, 29.3 (2), 29.1, 26.5, 26.0, 25.6 (2), 25.5, 25.0, 24.7, 22.7 (2), 20.6, 14.3, 14.1 (2) ppm (Supplementary Information, Figure S15); IR (ZnSe) 3013 (s, CH), 2921 (vs, CH), 2852 (s, CH), 1741 (vs, C=O) cm^−1^; HRMS *m/z* calcd. for C_51_H_90_O_5_ (M + NH_4_^+^) 800.7127, found 800.7131.

#### 3.1.16. Synthesis of 1-*O*-Octadecyl-2-dodecanoyl-3-eicosapentaenoyl-*sn*-glycerol (**6d**)

The same procedure was followed as described for **4a** using (*R*)-1-*O*-octadecyl-3-eicosapentaenoyl-*sn*-glycerol **6** (98 mg, 0.156 mmol), dodecanoic acid (30 mg, 0.150 mmol), DMAP (20 mg, 0.164 mmol) and EDAC (49 mg, 0.256 mmol) in 1 mL CH_2_Cl_2_. The product **6d** (115 mg, 0.142 mmol) was afforded as pale yellow oil, yield 94%. ${\mathrm{[\alpha ]}}_{D}^{20}$ −7.5 (*c* 1.0, benzene). ^1^H NMR (400 MHz, CDCl_3_) δ 5.43–5.28 (m, 10H, =C*H*), 5.22–5.17 (m, 1H, CH_2_C*H*CH_2_), 4.34 (dd, 1H, *J* = 11.9 Hz, *J* = 3.7 Hz, C*H*_2_OCO), 4.17 (dd, 1H, *J* = 11.9 Hz, *J* = 6.5 Hz, C*H*_2_OCO), 3.57–3.50 (2xdd, 2H, *J* = 10.6 Hz, *J* = 5.3 Hz, CHC*H*_2_O), 3.47–3.38 (2xdt, 2H, *J* = 9.3 Hz, *J* = 6.6 Hz, OC*H*_2_CH_2_), 2.88–2.77 (m, 8H, =CC*H*_2_C=), 2.32 (2xt, 4H, *J* = 7.7 Hz, *J* = 7.5 Hz, C*H*_2_COO in EPA and SFA), 2.13–2.04 (m, 4H, =CC*H*_2_CH_3_ and =CC*H*_2_CH_2_), 1.69 (quintet (br), 2H, *J* = 7.5 Hz, C*H*_2_CH_2_COO in EPA), 1.65–1.58 (m, 2H, C*H*_2_CH_2_COO in SFA), 1.58–1.50 (m, 2H, OCH_2_C*H*_2_), 1.35–1.20 (m, 46H, C*H*_2_), 0.97 (t, 3H, *J* = 7.5 Hz, C*H*_3_ in EPA), 0.88 (t, 6H, *J* = 6.8 Hz, C*H*_3_) ppm; ^13^C NMR (CDCl_3_) δ 173.20 (β, C=O in SFA), 173.18 (α, C=O in EPA), 132.0, 128.9 (2), 128.6, 128.3, 128.2 (2), 128.1, 127.9, 127.0, 71.8, 70.0, 68.9, 62.9, 34.3, 33.5, 31.9 (2), 29.7 (9), 29.6 (5), 29.5 (2), 29.4, 29.3 (2), 29.1, 26.5, 26.0, 25.6 (2), 25.5, 25.0, 24.7, 22.7 (2), 20.6, 14.3, 14.1 (2) ppm; IR (ZnSe) 3013 (s, CH), 2922 (vs, CH), 2853 (s, CH), 1741 (vs, C=O) cm^−1^; HRMS *m/z* calcd. for C_53_H_94_O_5_ (M + NH_4_^+^) 828.7440, found 828.7437.

#### 3.1.17. Synthesis of 1-*O*-Octadecyl-2-tetradecanoyl-3-eicosapentaenoyl-*sn*-glycerol (**6e**)

The same procedure was followed as described for **4a** using (*R*)-1-*O*-octadecyl-3-eicosapentaenoyl-*sn*-glycerol **6** (58 mg, 0.093 mmol), tetradecanoic acid (24 mg, 0.105 mmol), DMAP (9 mg, 0.073 mmol) and EDAC (26 mg, 0.136 mmol) in 1 mL CH_2_Cl_2_. The product **6e** (68 mg, 0.081 mmol) was afforded as pale yellow oil, yield 87%. ${\mathrm{[\alpha ]}}_{D}^{20}$ −7.4 (*c* 0.95, benzene). ^1^H NMR (400 MHz, CDCl_3_) δ 5.43–5.28 (m, 10H, =C*H*), 5.22–5.17 (m, 1H, CH_2_C*H*CH_2_), 4.34 (dd, 1H, *J* = 11.9 Hz, *J* = 3.7 Hz, C*H*_2_OCO), 4.17 (dd, 1H, *J* = 11.9 Hz, *J* = 6.5 Hz, C*H*_2_OCO), 3.57–3.50 (2xdd, 2H, *J* = 10.6 Hz, *J* = 5.3 Hz, CHC*H*_2_O), 3.47–3.38 (2xdt, 2H, *J* = 9.3 Hz, *J* = 6.6 Hz, OC*H*_2_CH_2_), 2.88–2.79 (m, 8H, =CC*H*_2_C=), 2.32 (2xt, 4H, *J* = 7.6 Hz, *J* = 7.5 Hz, C*H*_2_COO in EPA and SFA), 2.13–2.04 (m, 4H, =CC*H*_2_CH_3_ and =CC*H*_2_CH_2_), 1.69 (quintet (br), 2H, *J* = 7.5 Hz, C*H*_2_CH_2_COO in EPA), 1.65–1.58 (m, 2H, C*H*_2_CH_2_COO in SFA), 1.57–1.50 (m, 2H, OCH_2_C*H*_2_), 1.35–1.20 (m, 50H, C*H*_2_), 0.97 (t, 3H, *J* = 7.5 Hz, C*H*_3_ in EPA), 0.88 (t, 6H, *J* = 6.8 Hz, C*H*_3_) ppm; ^13^C NMR (CDCl_3_) δ 173.15 (β, C=O in SFA), 173.12 (α, C=O in EPA), 132.0, 128.9 (2), 128.6, 128.3, 128.2 (2), 128.1, 127.9, 127.0, 71.8, 70.0, 69.0, 62.9, 34.3, 33.5, 31.9 (2), 29.7 (14), 29.6 (2), 29.5, 29.4 (2), 29.3 (2), 29.1, 26.5, 26.0, 25.6 (2), 25.5, 25.0, 24.7, 22.7 (2), 20.6, 14.3, 14.1 (2) ppm; IR (ZnSe) 3012 (s, CH), 2922 (vs, CH), 2853 (s, CH), 1741 (vs, C=O) cm^−1^; HRMS *m/z* calcd. for C_55_H_98_O_5_ (M + H^+^) 839.7487, found 839.7486.

#### 3.1.18. Synthesis of 1-*O*-Octadecyl-2-hexadecanoyl-3-eicosapentaenoyl-*sn*-glycerol (**6f**)

The same procedure was followed as described for **4a** using (*R*)-1-*O*-octadecyl-3-eicosapentaenoyl-*sn*-glycerol **6** (73 mg, 0.116 mmol), hexadecanoic acid (31 mg, 0.121 mmol), DMAP (18 mg, 0.147 mmol) and EDAC (39 mg, 0.203 mmol) in 1 mL CH_2_Cl_2_. The product **6f** (90 mg, 0.104 mmol) was afforded as colorless oil, yield 90%. ${\mathrm{[\alpha ]}}_{D}^{20}$ −6.6 (*c* 0.93, benzene). ^1^H NMR (400 MHz, CDCl_3_) δ 5.44–5.28 (m, 10H, =C*H*), 5.22–5.17 (m, 1H, CH_2_C*H*CH_2_), 4.34 (dd, 1H, *J* = 11.9 Hz, *J* = 3.7 Hz, C*H*_2_OCO), 4.17 (dd, 1H, *J* = 11.9 Hz, *J* = 6.5 Hz, C*H*_2_OCO), 3.57–3.50 (2xdd, 2H, *J* = 10.6 Hz, *J* = 5.3 Hz, CHC*H*_2_O), 3.47–3.38 (2xdt, 2H, *J* = 9.3 Hz, *J* = 6.6 Hz, OC*H*_2_CH_2_), 2.89–2.79 (m, 8H, =CC*H*_2_C=), 2.32 (2xt, 4H, *J* = 7.6 Hz, *J* = 7.5 Hz, C*H*_2_COO in EPA and SFA), 2.13–2.04 (m, 4H, =CC*H*_2_CH_3_ and =CC*H*_2_CH_2_), 1.69 (quintet (br), 2H, *J* = 7.5 Hz, C*H*_2_CH_2_COO in EPA), 1.65–1.57 (m, 2H, C*H*_2_CH_2_COO in SFA), 1.57–1.50 (m, 2H, OCH_2_C*H*_2_), 1.35–1.20 (m, 54H, C*H*_2_), 0.97 (t, 3H, *J* = 7.5 Hz, C*H*_3_ in EPA), 0.88 (t, 6H, *J* = 6.8 Hz, C*H*_3_) ppm; ^13^C NMR (CDCl_3_) δ 173.16 (β, C=O in SFA), 173.13 (α, C=O in EPA), 132.0, 128.9 (2), 128.6, 128.3, 128.2 (2), 128.1, 127.9, 127.0, 71.8, 70.1, 69.0, 62.9, 34.4, 33.5, 31.9 (2), 29.7 (17), 29.6, 29.5 (2), 29.4 (2), 29.3, 29.1, 26.5, 26.0, 25.6 (2), 25.5, 25.0, 24.7, 22.7 (2), 20.6, 14.3, 14.1 (2) ppm; IR (ZnSe) 3013 (s, CH), 2922 (vs, CH), 2852 (s, CH), 1742 (vs, C=O) cm^−1^; HRMS *m/z* calcd. for C_57_H_102_O_5_ (M + H^+^) 867.7800, found 867.7774.

#### 3.1.19. Synthesis of1-*O*-Octadecyl-2-hexanoyl-3-docosahexaenoyl-*sn*-glycerol (**7a**)

The same procedure was followed as described for **4a** using (*R*)-1-*O*-octadecyl-3-docosahexaenoyl-*sn*-glycerol **7** (68 mg, 0.104 mmol), hexanoic acid (17 mg, 0.146 mmol), DMAP (10 mg, 0.082 mmol) and EDAC (29 mg, 0.151 mmol) in 1 mL CH_2_Cl_2_. The product **7a** (71 mg, 0.098 mmol) was afforded as colorless oil, yield 94%. ${\mathrm{[\alpha ]}}_{D}^{20}$ −8.5 (*c* 0.95, benzene). ^1^H NMR (400 MHz, CDCl_3_) δ 5.43–5.28 (m, 12H, =C*H*), 5.22–5.17 (m, 1H, CH_2_C*H*CH_2_), 4.35 (dd, 1H, *J* = 11.9 Hz, *J* = 3.7 Hz, C*H*_2_OCO), 4.18 (dd, 1H, *J* = 11.9 Hz, *J* = 6.5 Hz, C*H*_2_OCO), 3.57–3.50 (2xdd, 2H, *J* = 10.6 Hz, *J* = 5.4 Hz, CHC*H*_2_O), 3.47–3.38 (2xdt, 2H, *J* = 9.3 Hz, *J* = 6.7 Hz, OC*H*_2_CH_2_), 2.88–2.80 (m, 10H, =CC*H*_2_C=), 2.41–2.35 (m, 4H, C*H*_2_C*H*_2_COO in DHA), 2.32 (t, 2H, *J* = 7.5 Hz, C*H*_2_COO in SFA), 2.11–2.04 (m, 2H, =CC*H*_2_CH_3_), 1.66–1.59 (m, 2H, C*H*_2_CH_2_COO in SFA), 1.59–1.50 (m, 2H, OCH_2_C*H*_2_), 1.35–1.21 (m, 34H, C*H*_2_), 0.97 (t, 3H, *J* = 7.5 Hz, C*H*_3_ in DHA), 0.89 (t, 3H, *J* = 6.8 Hz, C*H*_3_ in SFA), 0.88 (t, 3H, *J* = 7.0 Hz, C*H*_3_ in ether) ppm; ^13^C NMR (CDCl_3_) δ 173.10 (β, C=O in SFA), 172.66 (α, C=O in DHA), 132.0, 129.3, 128.6, 128.3 (2), 128.2, 128.1 (2), 128.0, 127.9, 127.8, 127.0, 71.8, 70.1, 69.0, 63.0, 34.3, 34.0, 31.9, 31.2, 29.7 (9), 29.6 (3), 29.5, 29.4, 26.0, 25.6 (3), 25.5, 24.6, 22.7 (2), 22.3, 20.6, 14.3, 14.1, 13.9 ppm; IR (ZnSe) 3013 (s, CH), 2923 (vs, CH), 2853 (s, CH), 1741 (vs, C=O) cm^−1^; HRMS *m/z* calcd. for C_49_H_84_O_5_ (M + H^+^) 753.6392, found 753.6379.

#### 3.1.20. Synthesis of 1-*O*-Octadecyl-2-octanoyl-3-docosahexaenoyl-*sn*-glycerol (**7b**)

The same procedure was followed as described for **4a** using (*R*)-1-*O*-octadecyl-3-docosahexaenoyl-*sn*-glycerol **7** (66 mg, 0.100 mmol), octanoic acid (18 mg, 0.125 mmol), DMAP (17 mg, 0.139 mmol) and EDAC (30 mg, 0.156 mmol) in 1 mL CH_2_Cl_2_. The product **7b** (73 mg, 0.093 mmol) was afforded as pale yellow oil, yield 94%. ${\mathrm{[\alpha ]}}_{D}^{20}$ −8.1 (*c* 0.94, benzene). ^1^H NMR (400 MHz, CDCl_3_ Hz, *J* = 3.7 Hz, C*H*_2_OCO), 4.17 (dd, 1H, *J* = 11.9 Hz, *J* = 6.5 Hz, C*H*_2_OCO), 3.57–3.50 (2xdd, 2H, *J* = 10.7 Hz, *J* = 5.5 Hz, CHC*H*_2_O), 3.47–3.38 (2xdt, 2H, *J* = 9.3 Hz, *J* = 6.7 Hz, OC*H*_2_CH_2_), 2.88–2.80 (m, 10H, =CC*H*_2_C=), 2.41–2.35 (m, 4H, C*H*_2_C*H*_2_COO in DHA), 2.32 (t, 2H, *J* = 7.5 Hz, C*H*_2_COO in SFA), 2.11–2.04 (m, 2H, =CC*H*_2_CH_3_), 1.65–1.58 (m, 2H, C*H*_2_CH_2_COO in SFA), 1.58–1.50 (m, 2H, OCH_2_C*H*_2_), 1.37–1.20 (m, 38H, C*H*_2_), 0.97 (t, 3H, *J* = 7.5 Hz, C*H*_3_ in DHA), 0.88 (t, 6H, *J* = 6.8 Hz, C*H*_3_) ppm; ^13^C NMR (CDCl_3_) δ 173.11 (β, C=O in SFA), 172.66 (α, C=O in DHA), 132.0, 129.3, 128.6, 128.3 (2), 128.2, 128.1 (2), 128.0, 127.9, 127.8, 127.0, 71.8, 70.0, 69.0, 63.0, 34.3, 34.0, 31.9, 31.7, 29.7 (9), 29.6 (2), 29.5, 29.4, 29.0, 28.9, 26.0, 25.6 (3), 25.5, 25.0, 22.7 (2), 22.6, 20.6, 14.3, 14.1 (2) ppm; IR (ZnSe) 3013 (s, CH), 2923 (vs, CH), 2853 (s, CH), 1742 (vs, C=O) cm^−1^; HRMS *m/z* calcd. for C_51_H_88_O_5_ (M + NH_4_^+^) 798.6970, found 798.6964.

#### 3.1.21. Synthesis of 1-*O*-Octadecyl-2-decanoyl-3-docosahexaenoyl-*sn*-glycerol (**7c**)

The same procedure was followed as described for **4a** using (*R*)-1-*O*-octadecyl-3-docosahexaenoyl-*sn*-glycerol **7** (157 mg, 0.240 mmol), decanoic acid (50 mg, 0.290 mmol), DMAP (31 mg, 0.254 mmol) and EDAC (77 mg, 0.402 mmol) in 1 mL CH_2_Cl_2_. The product **7c** (176 mg, 0.218 mmol) was afforded as colorless oil, yield 91%. ${\mathrm{[\alpha ]}}_{D}^{20}$ −7.9 (*c* 1.0, benzene). ^1^H NMR (400 MHz, CDCl_3_) δ 5.43–5.28 (m, 12H, =C*H*), 5.22–5.17 (m, 1H, CH_2_C*H*CH_2_), 4.35 (dd, 1H, *J* = 11.9 Hz, *J* = 3.7 Hz, C*H*_2_OCO), 4.17 (dd, 1H, *J* = 11.9 Hz, *J* = 6.5 Hz, C*H*_2_OCO), 3.57–3.50 (2xdd, 2H, *J* = 10.6 Hz, *J* = 5.4 Hz, CHC*H*_2_O), 3.47–3.38 (2xdt, 2H, *J* = 9.3 Hz, *J* = 6.7 Hz, OC*H*_2_CH_2_), 2.91–2.80 (m, 10H, =CC*H*_2_C=), 2.42–2.37 (m, 4H, C*H*_2_C*H*_2_COO in DHA), 2.32 (t, 2H, *J* = 7.5 Hz, C*H*_2_COO in SFA), 2.11–2.04 (m, 2H, *J* = 7.4 Hz, =CC*H*_2_CH_3_), 1.65–-1.58 (m, 2H, C*H*_2_CH_2_COO in SFA), 1.58–1.50 (m, 2H, OCH_2_C*H*_2_), 1.39–1.21 (m, 42H, C*H*_2_), 0.97 (t, 3H, *J* = 7.5 Hz, C*H*_3_ in DHA), 0.88 (t, 6H, *J* = 6.8 Hz, C*H*_3_) ppm; ^13^C NMR (CDCl_3_) δ 173.11 (β, C=O in SFA), 172.66 (α, C=O in DHA), 132.0, 129.3, 128.6, 128.3 (2), 128.2, 128.1 (2), 128.0, 127.9, 127.8, 127.0, 71.8, 70.0, 69.0, 63.0, 34.3, 34.0, 31.9 (2), 29.7 (11), 29.6 (2), 29.5, 29.4 (2), 29.3 (2), 29.1, 26.0, 25.6 (3), 25.5, 25.0, 22.7 (2), 20.6, 14.3, 14.1 (2) ppm; IR (ZnSe) 3014 (s, CH), 2922 (vs, CH), 2853 (s, CH), 1742 (vs, C=O) cm^−1^; HRMS *m/z* calcd. for C_53_H_92_O_5_ (M + H^+^) 809.7018, found 809.7011.

#### 3.1.22. Synthesis of 1-*O*-Octadecyl-2-dodecanoyl-3-docosahexaenoyl-*sn*-glycerol (**7d**)

The same procedure was followed as described for **4a** using (*R*)-1-*O*-octadecyl-3-docosahexaenoyl-*sn*-glycerol **7** (100 mg, 0.153 mmol), dodecanoic acid (33 mg, 0.165 mmol), DMAP (22 mg, 0.180 mmol) and EDAC (50 mg, 0.261 mmol) in 1 mL CH_2_Cl_2_. The product **7d** (112 mg, 0.134 mmol) was afforded as colorless oil, yield 88%. ${\mathrm{[\alpha ]}}_{D}^{20}$ −7.7 (*c* 1.0, benzene). ^1^H NMR (400 MHz, CDCl_3_) δ 5.43–5.28 (m, 12H, =C*H*), 5.22-5.17 (m, 1H, CH_2_C*H*CH_2_), 4.35 (dd, 1H, *J* = 11.9 Hz, *J* = 3.7 Hz, C*H*_2_OCO), 4.17 (dd, 1H, *J* = 11.9 Hz, *J* = 6.5 Hz, C*H*_2_OCO), 3.57–3.50 (2xdd, 2H, *J* = 10.6 Hz, *J* = 5.3 Hz, CHC*H*_2_O), 3.47–3.37 (2xdt, 2H, *J* = 9.3 Hz, *J* = 6.6 Hz, OC*H*_2_CH_2_), 2.88–2.80 (m, 10H, =CC*H*_2_C=), 2.41–2.35 (m, 4H, C*H*_2_C*H*_2_COO in DHA), 2.32 (t, 2H, *J* = 7.5 Hz, C*H*_2_COO in SFA), 2.11–2.04 (quintet, 2H, *J* = 7.5 Hz, =CC*H*_2_CH_3_), 1.65–1.57 (m, 2H, C*H*_2_CH_2_COO in SFA), 1.57–1.50 (m, 2H, OCH_2_C*H*_2_), 1.35–1.20 (m, 46H, C*H*_2_), 0.97 (t, 3H, *J* =.5 Hz, C*H*_3_ in DHA), 0.88 (t, 6H, *J* = 6.8 Hz, C*H*_3_) ppm (Supplementary Information, Figure S16); ^13^C NMR (CDCl_3_) δ 173.12 (β, C=O in SFA), 172.67 (α, C=O in DHA), 132.0, 129.3, 128.6, 128.3 (2), 128.2, 128.1 (2), 128.0, 127.9, 127.8, 127.0, 71.8, 70.0, 68.9, 63.0, 34.3, 34.0, 31.9 (2), 29.7 (10), 29.6 (5), 29.5 (2), 29.4, 29.3 (2), 29.1, 26.0, 25.6 (3), 25.5, 25.0, 22.7 (2), 20.6, 14.3, 14.1 (2) ppm (Supplementary Information, Figure S17); IR (ZnSe) 3014 (s, CH), 2922 (vs, CH), 2853 (s, CH), 1742 (vs, C=O) cm^−1^; HRMS *m/z* calcd. for C_55_H_96_O_5_ (M + NH_4_^+^) 854.7596, found 854.7607.

#### 3.1.23. Synthesis of 1-*O*-Octadecyl-2-tetradecanoyl-3-docosahexaenoyl-*sn*-glycerol (**7e**)

The same procedure was followed as described for **4a** using (*R*)-1-*O*-octadecyl-3-docosahexaenoyl-*sn*-glycerol **7** (59 mg, 0.092 mmol), tetradecanoic acid (23 mg, 0.101 mmol), DMAP (10 mg, 0.082 mmol) and EDAC (26 mg, 0.136 mmol) in 1 mL CH_2_Cl_2_. The product **7e** (73 mg, 0.084 mmol) was afforded as pale yellow oil, yield 94%. ${\mathrm{[\alpha ]}}_{D}^{20}$ −7.3 (*c* 0.91, benzene). ^1^H NMR (400 MHz, CDCl_3_) δ 5.43–5.28 (m, 12H, =C*H*), 5.22–5.17 (m, 1H, CH_2_C*H*CH_2_), 4.35 (dd, 1H, *J* = 11.9 Hz, *J* = 3.7 Hz, C*H*_2_OCO), 4.17 (dd, 1H, *J* = 11.9 Hz, *J* = 6.5 Hz, C*H*_2_OCO), 3.57–3.50 (2xdd, 2H, *J* = 10.6 Hz, *J* = 5.3 Hz, CHC*H*_2_O), 3.47–3.38 (2xdt, 2H, *J* = 9.3 Hz, *J* = 6.6 Hz, OC*H*_2_CH_2_), 2.88–2.80 (m, 10H, =CC*H*_2_C=), 2.41–2.35 (m, 4H, C*H*_2_C*H*_2_COO in DHA), 2.32 (t, 2H, *J* = 7.5 Hz, C*H*_2_COO in SFA), 2.11–2.04 (quintet, 2H, *J* = 7.5 Hz, =C*H*_2_CH_3_), 1.65–1.57 (m, 2H, C*H*_2_CH_2_COO in SFA), 1.57–1.50 (m, 2H, OCH_2_C*H*_2_), 1.35–1.20 (m, 50H, C*H*_2_), 0.97 (t, 3H, *J* = 7.5 Hz, C*H*_3_ in DHA), 0.88 (t, 6H, *J* = 6.8 Hz, C*H*_3_) ppm; ^13^C NMR (CDCl_3_) δ 173.12 (β, C=O in SFA), 172.67 (α, C=O in DHA), 132.0, 129.3, 128.6, 128.3 (2), 128.2, 128.1 (2), 128.0, 127.9, 127.8, 127.0, 71.8, 70.0, 68.9, 63.0, 34.3, 34.0, 31.9 (2), 29.7 (15), 29.6 (2), 29.5 (2), 29.4 (2), 29.3, 29.1, 26.0, 25.6 (3), 25.5, 25.0, 22.7 (2), 20.6, 14.3, 14.1 (2) ppm; IR (ZnSe) 3014 (s, CH), 2923 (vs, CH), 2853 (s, CH), 1743 (vs, C=O) cm^−1^; HRMS *m/z* calcd. for C_57_H_100_O_5_ (M + H^+^) 865.7644, found 865.7625.

#### 3.1.24. Synthesis of 1-*O*-Octadecyl-2-hexadecanoyl-3-docosahexaenoyl-*sn*-glycerol (**7f**)

The same procedure was followed as described for **4a** using (*R*)-1-*O*-octadecyl-3-docosahexaenoyl-*sn*-glycerol **7** (57 mg, 0.087 mmol), hexadecanoic acid (25 mg, 0.097 mmol), DMAP (12 mg, 0.098 mmol) and EDAC (26 mg, 0.136 mmol) in 1 mL CH_2_Cl_2_. The product **7f** (72 mg, 0.081 mmol) was afforded as pale yellow solid, yield 93%. Mp 29–31 °C. ${\mathrm{[\alpha ]}}_{D}^{20}$ −7.2 (*c* 0.79, benzene). ^1^H NMR (400 MHz, CDCl_3_) δ 5.43–5.28 (m, 12H, =C*H*), 5.22–5.17 (m, 1H, CH_2_C*H*CH_2_), 4.35 (dd, 1H, *J* = 11.9 Hz, *J* = 3.7 Hz, C*H*_2_OCO), 4.17 (dd, 1H, *J* = 11.9 Hz, *J* = 6.5 Hz, C*H*_2_OCO), 3.57–3.50 (2xdd, 2H, *J* = 10.6 Hz, *J* = 5.3 Hz, CHC*H*_2_O), 3.47–3.38 (2xdt, 2H, *J* = 9.3 Hz, *J* = 6.6 Hz, OC*H*_2_CH_2_), 2.88–2.80 (m, 10H, =CC*H*_2_C=), 2.41–2.35 (m, 4H, C*H*_2_C*H*_2_COO in DHA), 2.32 (t, 2H, *J* = 7.5 Hz, C*H*_2_COO in SFA), 2.11–2.04 (quintet, 2H, *J* = 7.5 Hz, =CC*H*_2_CH_3_), 1.65–1.58 (m, 2H, C*H*_2_CH_2_COO in SFA), 1.58–1.50 (m, 2H, OCH_2_C*H*_2_), 1.35–1.20 (m, 54H, C*H*_2_), 0.97 (t, 3H, *J* = 7.5 Hz, C*H*_3_ in DHA), 0.88 (t, 6H, *J* = 6.8 Hz, C*H*_3_) ppm; ^13^C NMR (CDCl_3_) δ 173.12 (β, C=O in SFA), 172.67 (α, C=O in DHA), 132.0, 129.3, 128.6, 128.3 (2), 128.2, 128.1 (2), 128.0, 127.9, 127.8, 127.0, 71.8, 70.0, 68.9, 63.0, 34.3, 34.0, 31.9 (2), 29.7 (17), 29.6, 29.5 (2), 29.4 (2), 29.3, 29.1, 26.0, 25.6 (3), 25.5, 25.0, 22.7 (3), 20.6, 14.3, 14.1 (2) ppm; IR (ZnSe) 3014 (s, CH), 2922 (vs, CH), 2853 (s, CH), 1743 (vs, C=O) cm^−1^; HRMS *m/z* calcd. for C_59_H_104_O_5_ (M + NH_4_^+^) 910.8222, found 910.8220.

#### 3.1.25. Synthesis of 1-*O*-(*Z*)-Octadec-9-enyl-2-hexanoyl-3-eicosapentaenoyl-*sn*-glycerol (**8a**)

The same procedure was followed as described for **4a** using (*R*)-1-*O*-(*Z*)-octadec-9-enyl-3-eicosapentaenoyl-*sn*-glycerol **8** (98 mg, 0.156 mmol), hexanoic acid (20 mg, 0.172 mmol), DMAP (18 mg, 0.147 mmol) and EDAC (50 mg, 0.261 mmol) in 1 mL CH_2_Cl_2_. The product **8a** (102 mg, 0.141 mmol) was afforded as pale yellow oil, yield 90%. ${\mathrm{[\alpha ]}}_{D}^{20}$ −7.7 (*c* 1.1, benzene). ^1^H NMR (400 MHz, CDCl_3_) δ 5.43–5.28 (m, 12H, =C*H*), 5.22–5.17 (m, 1H, CH_2_C*H*CH_2_), 4.34 (dd, 1H, *J* = 11.9 Hz, *J* = 3.7 Hz, C*H*_2_OCO), 4.17 (dd, 1H, *J* = 11.9 Hz, *J* = 6.5 Hz, C*H*_2_OCO), 3.57–3.50 (2xdd, 2H, *J* = 10.6, Hz *J* = 5.3 Hz, CHC*H*_2_O), 3.47–3.38 (2xdt, 2H, *J* = 9.3 Hz, *J* = 6.6 Hz, OC*H*_2_CH_2_), 2.88–2.79 (m, 8H, =CC*H*_2_C=), 2.32 (2xt, 4H, *J* = 7.6 Hz, *J* = 7.5 Hz, C*H*_2_COO in EPA and SFA), 2.13–2.06 (m, 4H, =CC*H*_2_CH_3_ and =CC*H*_2_CH_2_), 2.04–1.99 (m, 4H, =CC*H*_2 _in selachyl), 1.70 (quintet (br), 2H, *J* = 7.5 Hz, C*H*_2_CH_2_COO in EPA), 1.66–1.59 (m, 2H, C*H*_2_CH_2_COO in SFA), 1.59–1.50 (qunitet (br), 2H, *J* = 6.8 Hz, OCH_2_C*H*_2_), 1.37–1.22 (m, 26H, C*H*_2_), 0.97 (t, 3H, *J* = 7.5 Hz, C*H*_3_ in EPA), 0.89 (t, 3H, *J* = 6.9 Hz, C*H*_3_ in SFA), 0.88 (t, 3H, *J* = 7.0 Hz, C*H*_3_ in ether) ppm; ^13^C NMR (CDCl_3_) δ 173.15 (β, C=O in SFA), 173.11 (α, C=O in EPA), 132.0, 129.9, 129.8, 128.9 (2), 128.6, 128.3, 128.2 (2), 128.1, 127.9, 127.0, 71.7, 70.0, 69.0, 62.9, 34.3, 33.5, 31.9, 31.2, 29.8 (2), 29.7, 29.6, 29.5, 29.4, 29.3 (3), 27.2 (2), 26.5, 26.0, 25.6 (3), 25.5, 24.7, 24.6, 22.7, 22.3, 20.6, 14.3, 14.1, 13.9 ppm; IR (ZnSe) 3012 (s, CH), 2923 (vs, CH), 2854 (s, CH), 1741 (vs, C=O) cm^−1^; HRMS *m/z* calcd. for C_47_H_80_O_5_ (M + NH_4_^+^) 742.6344, found 742.6336.

#### 3.1.26. Synthesis of 1-*O*-(*Z*)-Octadec-9-enyl-2-octanoyl-3-eicosapentaenoyl-*sn*-glycerol (**8b**)

The same procedure was followed as described for **4a** using (*R*)-1-*O*-(*Z*)-octadec-9-enyl-3-eicosapentaenoyl-*sn*-glycerol **8** (60 mg, 0.096 mmol), octanoic acid (15 mg, 0.104 mmol), DMAP (11 mg, 0.090 mmol) and EDAC (26 mg, 0.136 mmol) in 1 mL CH_2_Cl_2_. The product **8b** (62 mg, 0.082 mmol) was afforded as pale yellow oil, yield 86%. ${\mathrm{[\alpha ]}}_{D}^{20}$ −7.5 (*c* 0.88, benzene). ^1^H NMR (400 MHz, CDCl_3_) δ 5.43–5.28 (m, 12H, =C*H*), 5.22–5.17 (m, 1H, CH_2_C*H*CH_2_), 4.34 (dd, 1H, *J* = 11.9 Hz, *J* = 3.7 Hz, C*H*_2_OCO), 4.17 (dd, 1H, *J* = 11.9 Hz, *J* = 6.5 Hz, C*H*_2_OCO), 3.57–3.50 (2xdd, 2H, *J* = 10.6 Hz, *J* = 5.3 Hz, CHC*H*_2_O), 3.47–3.38 (2xdt, 2H, *J* = 9.3 Hz, *J* = 6.6 Hz, OC*H*_2_CH_2_), 2.88–2.77 (m, 8H, =CC*H*_2_C=), 2.32 (2xt, 4H, *J* = 7.6 Hz, *J* = 7.5 Hz, C*H*_2_COO in EPA and SFA), 2.13–2.04 (m, 4H, =CC*H*_2_CH_3_ and =CC*H*_2_CH_2_), 2.04–1.99 (m, 4H, =CC*H*_2 _in selachyl), 1.69 (quintet (br), 2H, *J* = 7.5 Hz, C*H*_2_CH_2_COO in EPA), 1.63–1.58 (m, 2H, C*H*_2_CH_2_COO in SFA), 1.58–1.50 (quintet (br), 2H, *J* = 6.8 Hz, OCH_2_C*H*_2_), 1.39–1.22 (m, 30H, C*H*_2_), 0.97 (t, 3H, *J* = 7.5 Hz, C*H*_3_ in EPA), 0.88 (t, 6H, *J* = 6.9 Hz, C*H*_3_) ppm; ^13^C NMR (CDCl_3_) δ 173.15 (β, C=O in SFA), 173.11 (α, C=O in EPA), 132.0, 129.9, 129.8, 128.9 (2), 128.6, 128.3, 128.2 (2), 128.1, 127.9, 127.0, 71.7, 70.0, 69.0, 62.9, 34.3, 33.5, 31.9, 31.7, 29.8 (2), 29.7, 29.6, 29.5 (2), 29.4, 29.3 (3), 29.0, 28.9, 27.2 (2), 26.5, 26.0, 25.6 (2), 25.5, 25.0, 24.7, 22.7, 22.6, 20.6, 14.3, 14.1 (2) ppm; IR (ZnSe) 3012 (s, CH), 2924 (vs, CH), 2854 (s, CH), 1741 (vs, C=O) cm^−1^; HRMS *m/z* calcd. for C_49_H_84_O_5_ (M + NH_4_^+^) 770.6657, found 770.6657.

#### 3.1.27. Synthesis of 1-*O*-(*Z*)-Octadec-9-enyl-2-decanoyl-3-eicosapentaenoyl-*sn*-glycerol (**8c**)

The same procedure was followed as described for **4a** using (*R*)-1-*O*-(*Z*)-octadec-9-enyl-3-eicosapentaenoyl-*sn*-glycerol **8** (60 mg, 0.096 mmol), decanoic acid (18 mg, 0.104 mmol), DMAP (8 mg, 0.067 mmol) and EDAC (27 mg, 0.141 mmol) in 1 mL CH_2_Cl_2_. The product **8c** (64 mg, 0.082 mmol) was afforded as pale yellow oil, yield 86%. ${\mathrm{[\alpha ]}}_{D}^{20}$ −6.8 (*c* 0.95, benzene). ^1^H NMR (400 MHz, CDCl_3_) δ 5.43–5.28 (m, 12H, =C*H*), 5.22–5.17 (m, 1H, CH_2_C*H*CH_2_), 4.34 (dd, 1H, *J* = 11.9 Hz, *J* = 3.7 Hz, C*H*_2_OCO), 4.17 (dd, 1H, *J* = 11.9 Hz, *J* = 6.5 Hz, C*H*_2_OCO), 3.57–3.50 (2xdd, 2H, *J* = 10.6 Hz, *J* = 5.3 Hz, CHC*H*_2_O), 3.47–3.38 (2xdt, 2H, *J* = 9.3 Hz, *J* = 6.6 Hz, OC*H*_2_CH_2_), 2.88–2.77 (m, 8H, =CC*H*_2_C=), 2.32 (2xt, 4H, *J* = 7.6 Hz, *J* = 7.5 Hz, C*H*_2_COO in EPA and SFA), 2.13–2.04 (m, 4H, =CC*H*_2_CH_3_ and =CC*H*_2_CH_2_), 2.04–1.99 (m, 4H, =CC*H*_2 _in selachyl), 1.69 (quintet (br), 2H, *J* = 7.5 Hz, C*H*_2_CH_2_COO in EPA), 1.65–1.58 (m, 2H, C*H*_2_CH_2_COO in SFA), 1.58–1.50 (quintet (br), 2H, *J* = 6.8 Hz, OCH_2_C*H*_2_), 1.39–1.22 (m, 34H, C*H*_2_), 0.97 (t, 3H, *J* = 7.5 Hz, C*H*_3_ in EPA), 0.88 (t, 6H, *J* = 6.7 Hz, C*H*_3_) ppm; ^13^C NMR (CDCl_3_) δ 173.15 (β, C=O in SFA), 173.12 (α, C=O in EPA), 132.0, 129.9, 129.8, 128.9 (2), 128.6, 128.3, 128.2 (2), 128.1, 127.9, 127.0, 71.7, 70.0, 69.0, 62.9, 34.3, 33.5, 31.9 (2), 29.8 (2), 29.7, 29.6, 29.5 (2), 29.4 (2), 29.3 (5), 29.1, 27.2 (2), 26.5, 26.0, 25.6 (2), 25.5, 24.9, 24.7, 22.7 (2), 20.6, 14.3, 14.1 (2) ppm; IR (ZnSe) 3012 (s, CH), 2923 (vs, CH), 2854 (s, CH), 1742 (vs, C=O) cm^−1^; HRMS *m/z* calcd. for C_51_H_88_O_5_ (M + NH_4_^+^) 798.6970, found 798.6940.

#### 3.1.28. Synthesis of 1-*O*-(*Z*)-Octadec-9-enyl-2-dodecanoyl-3-eicosapentaenoyl-*sn*-glycerol (**8d**)

The same procedure was followed as described for **4a** using (*R*)-1-*O*-(*Z*)-octadec-9-enyl-3-eicosapentaenoyl-*sn*-glycerol **8** (60 mg, 0.096 mmol), dodecanoic acid (21 mg, 0.105 mmol), DMAP (13 mg, 0.107 mmol) and EDAC (27 mg, 0.141 mmol) in 1 mL CH_2_Cl_2_. The product **8d** (74 mg, 0.091 mmol) was afforded as pale yellow oil, yield 96%. ${\mathrm{[\alpha ]}}_{D}^{20}$ −6.8 (*c* 0.87, benzene). ^1^H NMR (400 MHz, CDCl_3_) δ 5.43–5.28 (m, 12H, =C*H*), 5.22–5.17 (m, 1H, CH_2_C*H*CH_2_), 4.34 (dd, 1H, *J* = 11.9 Hz, *J* = 3.7 Hz, C*H*_2_OCO), 4.17 (dd, 1H, *J* = 11.9 Hz, *J* = 6.5 Hz, C*H*_2_OCO), 3.57–3.50 (2xdd, 2H, *J* = 10.6 Hz, *J* = 5.3 Hz, CHC*H*_2_O), 3.47–3.38 (2xdt, 2H, *J* = 9.3 Hz, *J* = 6.6 Hz, OC*H*_2_CH_2_), 2.87–2.77 (m, 8H, =CC*H*_2_C=), 2.32 (2xt, 4H, *J* = 7.6 Hz, *J* = 7.5 Hz, C*H*_2_COO in EPA and SFA), 2.13–2.04 (m, 4H, =CC*H*_2_CH_3_ and =CC*H*_2_CH_2_), 2.04–1.99 (m, 4H, =CC*H*_2 _in selachyl), 1.69 (quinted (br), 2H, *J* = 7.5 Hz, C*H*_2_CH_2_COO in EPA), 1.65–1.58 (m, 2H, C*H*_2_CH_2_COO in SFA), 1.58–1.50 (quintet (br), 2H, *J* = 6.8 Hz, OCH_2_C*H*_2_), 1.39–1.22 (m, 38H, C*H*_2_), 0.97 (t, 3H, *J* = 7.5 Hz, C*H*_3_ in EPA), 0.88 (t, 6H, *J* = 6.7 Hz, C*H*_3_) ppm; ^13^C NMR (CDCl_3_) δ 173.15 (β, C=O in SFA), 173.12 (α, C=O in EPA), 132.0, 129.9, 129.8, 128.9 (2), 128.6, 128.3, 128.2 (2), 128.1, 127.9, 127.0, 71.7, 70.0, 69.0, 62.9, 34.3, 33.5, 31.9 (2), 29.8 (2), 29.7, 29.6 (3), 29.5 (3), 29.4, 29.3 (5), 29.1, 27.2 (2), 26.5, 26.0, 25.6 (2), 25.5, 25.0, 24.7, 22.7 (2), 20.6, 14.3, 14.1 (2) ppm; IR (ZnSe) 3012 (s, CH), 2923 (vs, CH), 2853 (s, CH), 1742 (vs, C=O) cm^−1^; HRMS *m/z* calcd. for C_53_H_92_O_5_ (M + H^+^) 809.7018, found 809.7008.

#### 3.1.29. Synthesis of 1-*O*-(*Z*)-Octadec-9-enyl-2-tetradecanoyl-3-eicosapentaenoyl-*sn*-glycerol (**8e**)

The same procedure was followed as described for **4a** using (*R*)-1-*O*-(*Z*)-octadec-9-enyl-3-eicosapentaenoyl-*sn*-glycerol **8** (60 mg, 0.096 mmol), tetradecanoic acid (23 mg, 0.101 mmol), DMAP (10 mg, 0.082 mmol) and EDAC (29 mg, 0.150 mmol) in 1 mL CH_2_Cl_2_. The product **8e** (72 mg, 0.086 mmol) was afforded as pale yellow oil, yield 90%. ${\mathrm{[\alpha ]}}_{D}^{20}$ −6.7 (*c* 0.90, benzene). ^1^H NMR (400 MHz, CDCl_3_) δ 5.43–5.29 (m, 12H, =C*H*), 5.22–5.17 (m, 1H, CH_2_C*H*CH_2_), 4.34 (dd, 1H, *J* = 11.9 Hz, *J* = 3.7 Hz, C*H*_2_OCO), 4.17 (dd, 1H, *J* = 11.9 Hz, *J* = 6.5 Hz, C*H*_2_OCO), 3.57–3.50 (2xdd, 2H, *J* = 10.6 Hz, *J* = 5.3 Hz, CHC*H*_2_O), 3.47–3.38 (2xdt, 2H, *J* = 9.3 Hz, *J* = 6.6 Hz, OC*H*_2_CH_2_), 2.88–2.77 (m, 8H, =CC*H*_2_C=), 2.31 (2xt, 4H, *J* = 7.6 Hz, *J* = 7.5 Hz, C*H*_2_COO in EPA and SFA), 2.13–2.04 (m, 4H, =CC*H*_2_CH_3_ and =CC*H*_2_CH_2_), 2.04–1.99 (m, 4H, =CC*H*_2 _in selachyl), 1.69 (quintet (br), 2H, *J* = 7.5 Hz, C*H*_2_CH_2_COO in EPA), 1.65–1.58 (m, 2H, C*H*_2_CH_2_COO in SFA), 1.58–1.50 (quintet (br), 2H, *J* = 6.8 Hz, OCH_2_C*H*_2_), 1.39–1.22 (m, 42H, C*H*_2_), 0.97 (t, 3H, *J* = 7.5 Hz, C*H*_3_ in EPA), 0.88 (t, 6H, *J* = 6.8 Hz, C*H*_3_) ppm (Supplementary Information, Figure S18); ^13^C NMR (CDCl_3_) δ 173.15 (β, C=O in SFA), 173.12 (α, C=O in EPA), 132.0, 129.9, 129.8, 128.9 (2), 128.6, 128.3, 128.2 (2), 128.1, 127.9, 127.0, 71.7, 70.0, 69.0, 62.9, 34.3, 33.5, 31.9 (2), 29.8 (2), 29.7 (3), 29.6 (2), 29.5 (3), 29.4 (2), 29.3 (5), 29.1, 27.2 (2), 26.5, 26.0, 25.6 (2), 25.5, 25.0, 24.7, 22.7 (2), 20.6, 14.3, 14.1 (2) ppm (Supplementary Information, Figure S19); IR (ZnSe) 3012 (s, CH), 2922 (vs, CH), 2853 (s, CH), 1742 (vs, C=O) cm^−1^; HRMS *m/z* calcd. for C_55_H_96_O_5_ (M + NH_4_^+^) 854.7596, found 854.7611.

#### 3.1.30. Synthesis of 1-*O*-(*Z*)-Octadec-9-enyl-2-hexadecanoyl-3-eicosapentaenoyl-*sn*-glycerol (**8f**)

The same procedure was followed as described for **4a** using (*R*)-1-*O*-(*Z*)-octadec-9-enyl-3-eicosapentaenoyl-*sn*-glycerol **8** (70 mg, 0.112 mmol), hexadecanoic acid (31 mg, 0.121 mmol), DMAP (13 mg, 0.107 mmol) and EDAC (33 mg, 0.172 mmol) in 1 mL CH_2_Cl_2_. The product **8f** (83 mg, 0.095 mmol) was afforded as pale yellow oil, yield 86%. ${\mathrm{[\alpha ]}}_{D}^{20}$ −6.7 (*c* 0.86, benzene). ^1^H NMR (400 MHz, CDCl_3_) δ 5.43–5.28 (m, 12H, =C*H*), 5.22–5.17 (m, 1H, CH_2_C*H*CH_2_), 4.34 (dd, 1H, *J* = 11.9 Hz, *J* = 3.7 Hz, C*H*_2_OCO), 4.17 (dd, 1H, *J* = 11.9 Hz, *J* = 6.5 Hz, C*H*_2_OCO), 3.57–3.50 (2xdd, 2H, *J* = 10.6 Hz, *J* = 5.3 Hz, CHC*H*_2_O), 3.47–3.37 (2xdt, 2H, *J* = 9.3 Hz, *J* = 6.6 Hz, OC*H*_2_CH_2_), 2.88–2.77 (m, 8H, =CC*H*_2_C=), 2.31 (2xt, 4H, *J* = 7.6 Hz, *J* = 7.5 Hz, C*H*_2_COO in EPA and SFA), 2.13–2.04 (m, 4H, =CC*H*_2_CH_3_ and =CC*H*_2_CH_2_), 2.04–1.99 (m, 4H, =CC*H*_2 _in selachyl), 1.69 (quintet (br), 2H, *J* = 7.5 Hz, C*H*_2_CH_2_COO in EPA), 1.65–1.58 (m, 2H, C*H*_2_CH_2_COO in SFA), 1.58–1.50 (quintet (br), 2H, *J* = 6.8 Hz, OCH_2_C*H*_2_), 1.38–1.20 (m, 46H, C*H*_2_), 0.97 (t, 3H, *J* = 7.5 Hz, C*H*_3_ in EPA), 0.88 (t, 6H, *J* = 6.8 Hz, C*H*_3_) ppm; ^13^C NMR (CDCl_3_) δ 173.15 (β, C=O in SFA), 173.12 (α, C=O in EPA), 132.0, 129.9, 129.8, 128.9 (2), 128.6, 128.3, 128.2 (2), 128.1, 127.9, 127.0, 71.7, 70.0, 69.0, 62.9, 34.3, 33.5, 31.9 (2), 29.8 (2), 29.7 (6), 29.6 (2), 29.5 (3), 29.4 (2), 29.3 (4), 29.1, 27.2 (2), 26.5, 26.0, 25.6 (2), 25.5, 25.0, 24.7, 22.7 (2), 20.6, 14.3, 14.1 (2) ppm; IR (ZnSe) 3012 (s, CH), 2922 (vs, CH), 2853 (s, CH), 1742 (vs, C=O) cm^−1^; HRMS *m/z* calcd. for C_57_H_100_O_5_ (M + NH_4_^+^) 882.7909, found 882.7901.

#### 3.1.31. Synthesis of 1-*O*-(*Z*)-Octadec-9-enyl-2-hexanoyl-3-docosahexaenoyl-*sn*-glycerol (**9a**)

The same procedure was followed as described for **4a** using (*R*)-1-*O*-(Z)-octadec-9-enyl-3-docosahexaenoyl-*sn*-glycerol **9** (99 mg, 0.152 mmol), hexanoic acid (20 mg, 0.172 mmol), DMAP (20 mg, 0.164 mmol) and EDAC (41 mg, 0.214 mmol) in 1 mL CH_2_Cl_2_. The product **9a** (105 mg, 0.140 mmol) was afforded as colorless oil, yield 92%. ${\mathrm{[\alpha ]}}_{D}^{20}$ −8.3 (*c* 0.83, benzene). ^1^H NMR (400 MHz, CDCl_3_) δ 5.43–5.28 (m, 14H, =C*H*), 5.22–5.17 (m, 1H, CH_2_C*H*CH_2_), 4.35 (dd, 1H, *J* = 11.9 Hz, *J* = 3.7 Hz, C*H*_2_OCO), 4.17 (dd, 1H, *J* = 11.9 Hz, *J* = 6.5 Hz, C*H*_2_OCO), 3.57–3.50 (2xdd, 2H, *J* = 10.6, Hz *J* = 5.3 Hz, CHC*H*_2_O), 3.47–3.37 (2xdt, 2H, *J* = 9.3 Hz, *J* = 6.6 Hz, OC*H*_2_CH_2_), 2.88–2.79 (m, 10H, =CC*H*_2_C=), 2.39–2.35 (m, 4H, C*H*_2_C*H*_2_COO in DHA), 2.32 (t, 2H, *J* = 7.5 Hz, C*H*_2_COO in SFA), 2.11–2.04 (m, 2H, =CC*H*_2_CH_3_), 2.03–1.98 (m, 4H, =CC*H*_2 _in selachyl), 1.66–1.60 (m, 2H, C*H*_2_CH_2_COO in SFA), 1.60–1.50 (m, 2H, OCH_2_C*H*_2_), 1.34–1.23 (m, 26H, C*H*_2_), 0.97 (t, 3H, *J* = 7.5 Hz, C*H*_3_ in DHA), 0.89 (t, 3H, *J* = 6.9 Hz, C*H*_3_ in SFA), 0.88 (t, 3H, *J* = 7.0 Hz, C*H*_3_ in ether) ppm; ^13^C NMR (CDCl_3_) δ 173.12 (β, C=O in SFA), 172.68 (α, C=O in DHA), 132.0, 129.9, 129.8, 129.3, 128.6, 128.3 (2), 128.2, 128.1 (2), 128.0, 127.9, 127.8, 127.0, 71.8, 70.0, 69.0, 63.0, 34.3, 34.0, 31.9, 31.2, 30.9, 29.8 (2), 29.6, 29.5 (2), 29.4, 29.3 (3), 27.2 (2), 26.0, 25.6 (3), 25.5, 24.6, 24.6, 22.7, 22.3, 20.6, 14.3, 14.1, 13.9 ppm; IR (ZnSe) 3012 (s, CH), 2924 (vs, CH), 2854 (s, CH), 1741 (vs, C=O) cm^−1^; HRMS *m/z* calcd. for C_49_H_82_O_5_ (M + NH_4_^+^) 768.6501, found 768.6513.

#### 3.1.32. Synthesis of 1-*O*-(*Z*)-Octadec-9-enyl-2-octanoyl-3-docosahexaenoyl-*sn*-glycerol (**9b**)

The same procedure was followed as described for **4a** using (*R*)-1-*O*-(Z)-octadec-9-enyl-3-docosahexaenoyl-*sn*-glycerol **9** (92 mg, 0.141 mmol), octanoic acid (26 mg, 0.180 mmol), DMAP (17 mg, 0.139 mmol) and EDAC (39 mg, 0.203 mmol) in 1 mL CH_2_Cl_2_. The product **9b** (98 mg, 0.126 mmol) was afforded as colorless oil, yield 89%. ${\mathrm{[\alpha ]}}_{D}^{20}$ −7.3 (*c* 0.84, benzene). ^1^H NMR (400 MHz, CDCl_3_) δ 5.43–5.28 (m, 14H, =C*H*), 5.22–5.17 (m, 1H, CH_2_C*H*CH_2_), 4.35 (dd, 1H, *J* = 11.9 Hz, *J* = 3.7 Hz, C*H*_2_OCO), 4.17 (dd, 1H, *J* = 11.9 Hz, *J* = 6.5 Hz, C*H*_2_OCO), 3.57–3.50 (2xdd, 2H, *J* = 10.6 Hz, *J* = 5.3 Hz, CHC*H*_2_O), 3.47–3.37 (2xdt, 2H, *J* = 9.3 Hz, *J* = 6.6 Hz, OC*H*_2_CH_2_), 2.88–2.80 (m, 10H, =CC*H*_2_C=), 2.39–2.35 (m, 4H, C*H*_2_C*H*_2_COO in DHA), 2.32 (t, 2H, *J* = 7.5 Hz, C*H*_2_COO in SFA), 2.11–2.03 (m, 2H, =CC*H*_2_CH_3_), 2.03–1.98 (m, 4H, =CC*H*_2 _in selachyl), 1.65–1.58 (m, 2H, C*H*_2_CH_2_COO in SFA), 1.58–1.50 (m, 2H, OCH_2_C*H*_2_), 1.38–1.21 (m, 30H, C*H*_2_), 0.97 (t, 3H, *J* = 7.5 Hz, C*H*_3_ in DHA), 0.88 (t, 6H, *J* = 6.7 Hz, C*H*_3_ ppm; ^13^C NMR (CDCl_3_) δ 173.12 (β, C=O in SFA), 172.68 (α, C=O in DHA), 132.0, 129.9, 129.8, 129.3, 128.6, 128.3 (2), 128.2, 128.1 (2), 128.0, 127.9, 127.8, 127.0, 71.8, 70.0, 69.0, 63.0, 34.3, 34.0, 31.9, 31.7, 30.9, 29.8 (2), 29.6, 29.5 (2), 29.4, 29.3 (3), 29.0, 28.9, 27.2 (2), 26.0, 25.6 (3), 25.5, 25.0, 22.7 (2), 22.6, 20.6, 14.3, 14.1 (2) ppm; IR (ZnSe) 3013 (s, CH), 2924 (vs, CH), 2854 (s, CH), 1742 (vs, C=O) cm^−1^; HRMS *m/z* calcd. for C_51_H_86_O_5_ (M + H^+^) 779.6548, found 779.6550.

#### 3.1.33. Synthesis of 1-*O*-(*Z*)-Octadec-9-enyl-2-decanoyl-3-docosahexaenoyl-*sn*-glycerol (**9c**)

The same procedure was followed as described for **4a** using (*R*)-1-*O*-(Z)-octadec-9-enyl-3-docosahexaenoyl-*sn*-glycerol **9** (106 mg, 0.162 mmol), decanoic acid (32 mg, 0.186 mmol), DMAP (23 mg, 0.188 mmol) and EDAC (47 mg, 0.245 mmol) in 1 mL CH_2_Cl_2_. The product **9c** (120 mg, 0.149 mmol) was afforded as colorless oil, yield 92%. ${\mathrm{[\alpha ]}}_{D}^{20}$ −7.7 (*c* 0.88, benzene). ^1^H NMR (400 MHz, CDCl_3_) δ 5.43–5.28 (m, 14H, =C*H*), 5.22–5.17 (m, 1H, CH_2_C*H*CH_2_), 4.35 (dd, 1H, *J* = 11.9 Hz, *J* = 3.7 Hz, C*H*_2_OCO), 4.17 (dd, 1H, *J* = 11.9 Hz, *J* = 6.5 Hz, C*H*_2_OCO), 3.57–3.50 (2xdd, 2H, *J* = 10.6 Hz, *J* = 5.3 Hz, CHC*H*_2_O), 3.47–3.37 (2xdt, 2H, *J* = 9.3 Hz, *J* = 6.6 Hz, OC*H*_2_CH_2_), 2.88–2.80 (m, 10H, =CC*H*_2_C=), 2.41–2.35 (m, 4H, C*H*_2_C*H*_2_COO in DHA), 2.32 (t, 2H, *J* = 7.5 Hz, C*H*_2_COO in SFA), 2.11–2.03 (m, 2H, =CC*H*_2_CH_3_), 2.03–1.98 (m, 4H, =CC*H*_2 _in selachyl), 1.65–1.58 (m, 2H, C*H*_2_CH_2_COO in SFA), 1.58–1.50 (m, 2H, OCH_2_C*H*_2_), 1.39–1.21 (m, 34H, C*H*_2_), 0.97 (t, 3H, *J* = 7.5 Hz, C*H*_3_ in DHA), 0.88 (t, 6H, *J* = 6.8 Hz, C*H*_3_) ppm; ^13^C NMR (CDCl_3_) δ 173.12 (β, C=O in SFA), 172.68 (α, C=O in DHA), 132.0, 129.9, 129.8, 129.3, 128.6, 128.3 (2), 128.2, 128.1 (2), 128.0, 127.9, 127.8, 127.0, 71.8, 70.0, 69.0, 63.0, 34.3, 34.0, 31.9 (2), 30.9, 29.8 (2), 29.6, 29.5 (2), 29.4 (2), 29.3 (5), 29.1, 27.2 (2), 26.0, 25.6 (3), 25.5, 25.0, 22.7 (3), 20.6, 14.3, 14.1 (2) ppm; IR (ZnSe) 3013 (s, CH), 2923 (vs, CH), 2854 (s, CH), 1742 (vs, C=O) cm^−1^; HRMS *m/z* calcd. for C_53_H_90_O_5_ (M + NH_4_^+^) 824.7127, found 824.7128.

#### 3.1.34. Synthesis of 1-*O*-(*Z*)-Octadec-9-enyl-2-dodecanoyl-3-docosahexaenoyl-*sn*-glycerol (**9d**)

The same procedure was followed as described for **4a** using (*R*)-1-*O*-(Z)-octadec-9-enyl-3-docosahexaenoyl-*sn*-glycerol **9** (55 mg, 0.084 mmol), dodecanoic acid (18 mg, 0.090 mmol), DMAP (11 mg, 0.090 mmol) and EDAC (23 mg, 0.120 mmol) in 1 mL CH_2_Cl_2_. The product **9d** (64 mg, 0.077 mmol) was afforded as pale yellow oil, yield 91%. ${\mathrm{[\alpha ]}}_{D}^{20}$ −7.6 (*c* 1.0, benzene). ^1^H NMR (400 MHz, CDCl_3_) δ 5.43–5.28 (m, 14H, =C*H*), 5.22–5.17 (m, 1H, CH_2_C*H*CH_2_), 4.35 (dd, 1H, *J* = 11.9 Hz, *J* = 3.7 Hz, C*H*_2_OCO), 4.17 (dd, 1H, *J* = 11.9 Hz, *J* = 6.5 Hz, C*H*_2_OCO), 3.57–3.50 (2xdd, 2H, *J* = 10.6 Hz, *J* = 5.3 Hz, CHC*H*_2_O), 3.47–3.37 (2xdt, 2H, *J* = 9.3 Hz, *J* = 6.6 Hz, OC*H*_2_CH_2_), 2.88–2.79 (m, 10H, =CC*H*_2_C=), 2.39–2.35 (m, 4H, C*H*_2_C*H*_2_COO in DHA), 2.32 (t, 2H, *J* = 7.5 Hz, C*H*_2_COO in SFA), 2.11–2.03 (m, 2H, CC*H*_2_CH_3_), 2.03–1.98 (m, 4H, =CC*H*_2 _in selachyl), 1.65–1.57 (m, 2H, C*H*_2_CH_2_COO in SFA), 1.57–1.50 (m, 2H, OCH_2_C*H*_2_), 1.38–1.22 (m, 36H, C*H*_2_), 0.97 (t, 3H, *J* = 7.5 Hz, C*H*_3_ in DHA), 0.88 (t, 6H, *J* = 6.8 Hz, C*H*_3_) ppm; ^13^C NMR (CDCl_3_) δ 173.13 (β, C=O in SFA), 172.68 (α, C=O in DHA), 132.0, 129.9, 129.8, 129.3, 128.6, 128.3 (2), 128.2, 128.1 (2), 128.0, 127.9, 127.8, 127.0, 71.8, 70.0, 69.0, 63.0, 34.3, 34.0, 31.9 (2), 30.9, 29.8 (2), 29.6 (3), 29.5 (3), 29.4, 29.3 (5), 29.1, 27.2 (2), 26.0, 25.6 (3), 25.5, 25.0, 22.7 (3), 20.6, 14.3, 14.1 (2) ppm; IR (ZnSe) 3013 (s, CH), 2923 (vs, CH), 2853 (s, CH), 1742 (vs, C=O) cm^−1^; HRMS *m/z* calcd. for C_55_H_94_O_5_ (M + H^+^) 835.7174, found 835.7161.

#### 3.1.35. Synthesis of 1-*O*-(*Z*)-Octadec-9-enyl-2-tetradecanoyl-3-docosahexaenoyl-*sn*-glycerol (**9e**)

The same procedure was followed as described for **4a** using (*R*)-1-*O*-(Z)-octadec-9-enyl-3-docosahexaenoyl-*sn*-glycerol **9** (55 mg, 0.084 mmol), tetradecanoic acid (20 mg, 0.088 mmol), DMAP (7 mg, 0.059 mmol) and EDAC (27 mg, 0.141 mmol) in 1 mL CH_2_Cl_2_. The product **9e** (67 mg, 0.078 mmol) was afforded as pale yellow oil, yield 92%. ${\mathrm{[\alpha ]}}_{D}^{20}$ −8.0 (*c* 0.96, benzene). ^1^H NMR (400 MHz, CDCl_3_): δ 5.43–5.28 (m, 14H, =C*H*), 5.22–5.17 (m, 1H, CH_2_C*H*CH_2_), 4.35 (dd, 1H, *J* = 11.9 Hz, *J* = 3.7 Hz, C*H*_2_OCO), 4.17 (dd, 1H, *J* = 11.9 Hz, *J* = 6.5 Hz, C*H*_2_OCO), 3.57–3.50 (2xdd, 2H, *J* = 10.6 Hz, *J* = 5.3 Hz, CHC*H*_2_O), 3.47–3.37 (2xdt, 2H, *J* = 9.3 Hz, *J* = 6.6 Hz, OC*H*_2_CH_2_), 2.88–2.80 (m, 10H, =CC*H*_2_C=), 2.39–2.35 (m, 4H, C*H*_2_C*H*_2_COO in DHA), 2.32 (t, 2H, *J* = 7.5 Hz, C*H*_2_COO in SFA), 2.11–2.03 (m, 2H, =CC*H*_2_CH_3_), 2.03–1.98 (m, 4H, =CC*H*_2 _in selachyl), 1.65–1.56 (m, 2H, C*H*_2_CH_2_COO in SFA), 1.56–1.50 (m, 2H, OCH_2_C*H*_2_), 1.38–1.20 (m, 40H, C*H*_2_), 0.97 (t, 3H, *J* = 7.5 Hz, C*H*_3_ in DHA), 0.88 (t, 6H, *J* = 6.8 Hz, C*H*_3_) ppm; ^13^C NMR (CDCl_3_) δ 173.13 (β, C=O in SFA), 172.68 (α, C=O in DHA), 132.0, 129.9, 129.8, 129.3, 128.6, 128.3 (2), 128.2, 128.1 (2), 128.0, 127.9, 127.8, 127.0, 71.8, 70.0, 69.0, 63.0, 34.3, 34.0, 31.9 (2), 29.8 (2), 29.7 (4), 29.6 (2), 29.5 (3), 29.4 (2), 29.3 (4), 29.1, 27.2 (2), 26.0, 25.6 (3), 25.5, 25.0, 22.7 (3), 20.6, 14.3, 14.1 (2) ppm; IR (ZnSe) 3013 (s, CH), 2923 (vs, CH), 2853 (s, CH), 1742 (vs, C=O) cm^−1^; HRMS *m/z* calcd. for C_57_H_98_O_5_ (M + H^+^) 863.7487, found 863.7490.

#### 3.1.36. Synthesis of 1-*O*-(*Z*)-Octadec-9-enyl-2-hexadecanoyl-3-docosahexaenoyl-*sn*-glycerol (**9f**)

The same procedure was followed as described for **4a** using (*R*)-1-*O*-(Z)-octadec-9-enyl-3-docosahexaenoyl-*sn*-glycerol **9** (61 mg, 0.093 mmol), hexadecanoic acid (27 mg, 0.105 mmol), DMAP (10 mg, 0.082 mmol) and EDAC (25 mg, 0.130 mmol) in 1 mL CH_2_Cl_2_. The product **9f** (74 mg, 0.083 mmol) was afforded as pale yellow oil, yield 89%. ${\mathrm{[\alpha ]}}_{D}^{20}$ −7.1 (*c* 0.89, benzene). ^1^H NMR (400 MHz, CDCl_3_) δ 5.43–5.28 (m, 14H, =C*H*), 5.22–5.17 (m, 1H, CH_2_C*H*CH_2_), 4.35 (dd, 1H, *J* = 11.9 Hz, *J* = 3.7 Hz, C*H*_2_OCO), 4.17 (dd, 1H, *J* = 11.9 Hz, *J* = 6.5 Hz, C*H*_2_OCO), 3.57–3.50 (2xdd, 2H, *J* = 10.6 Hz, *J* = 5.3 Hz, CHC*H*_2_O), 3.47–3.37 (2xdt, 2H, *J* = 9.3 Hz, *J* = 6.6 Hz, OC*H*_2_CH_2_), 2.88–2.80 (m, 10H, =CC*H*_2_C=), 2.41–2.35 (m, 4H, C*H*_2_C*H*_2_COO in DHA), 2.32 (t, 2H, *J* = 7.5 Hz, C*H*_2_COO in SFA), 2.11–2.03 (m, 2H, =CC*H*_2_CH_3_), 2.03–1.99 (m, 4H, =CC*H*_2 _in selachyl), 1.65–1.58 (m, 2H, C*H*_2_CH_2_COO in SFA), 1.58–1.50 (m, 2H, OCH_2_C*H*_2_), 1.40–1.20 (m, 44H, C*H*_2_), 0.97 (t, 3H, *J* = 7.5 Hz, C*H*_3_ in DHA), 0.88 (t, 6H, *J* = 6.8 Hz, C*H*_3_) ppm (Supplementary Information, Figure S20); ^13^C NMR (CDCl_3_) δ 173.12 (β, C=O in SFA), 172.68 (α, C=O in DHA), 132.0, 129.9, 129.8, 129.3, 128.6, 128.3 (2), 128.2, 128.1 (2), 128.0, 127.9, 127.8, 127.0, 71.8, 70.0, 69.0, 63.0, 34.3, 34.0, 31.9 (2), 30.9, 29.8 (2), 29.7 (4), 29.6 (2), 29.5 (3), 29.4 (2), 29.3 (4), 29.1, 27.2 (2), 26.0, 25.6 (4), 25.5, 25.0, 22.7 (3), 20.6, 14.3, 14.1 (2) ppm (Supplementary Information, Figure S21); IR (ZnSe) 3013 (s, CH), 2923 (vs, CH), 2853 (s, CH), 1743 (vs, C=O) cm^−1^; HRMS *m/z* calcd. for C_59_H_102_O_5_ (M + NH_4_^+^) 908.8066, found 908.8088.

## 4. Conclusions

A focused library of 36 enantiopure reversed structured AML type DAGEs has been prepared highly efficiently by a two-step chemoenzymatic process starting from enantiopure chimyl, batyl and selachyl alcohols. The DAGEs constitute a pure EPA or DHA at the *sn*-3 position of their glycerol backbone and a pure saturated even carbon number fatty acid (C6:0–C16:0) at the *sn*-2 position. In a previous work, EPA and DHA activated as acetoxime esters were introduced exclusively to the terminal *sn*-3 position of the 1-*O*-alkyl-*sn*-glycerols to accomplish the 3-MAGE intermediates enantio- and regiopure in excellent yields of high chemical purity. No detrimental acyl-migration side reaction was observed to take place as a result of the mild conditions offered by the lipase. In the current work, the saturated fatty acids were introduced to the remaining *sn*-2 position in very high to excellent yields by aid of EDAC as a chemical coupling agent in the presence of DMAP serving as a base and catalyst during which no deterioration of the excellent regiocontrol was observed. This focused library adds to a similar library of 48 enantiopure normal structured ALM type DAGEs possessing EPA or DHA at the *sn*-2 position and saturated even number fatty acids (C2–C16:0) located at the *sn*-3 position of the glycerol moiety. The total number of ether lipid constituents amounts to 84 DAGE and 42 intermediate 3-MAGE compounds with the entire ether lipid library counting the total of 126 compounds.

This combined library of ether lipids offers multiple possibilities of applications as chemical standards for various analytical purposes, fine chemicals, offering lots of possibilities in screening for various bioactivities where bioactive *n*-3 PUFAs and ether lipids have been combined in a single molecule. They may also find use as drug carriers, prodrugs and even potent drugs. There are also lots of possibilities involved in attaching a potent drug component to the *sn*-2 of *sn*-3 positions of the glycerol framework in combination with EPA or DHA within the same molecule. The utility of such interesting prodrugs needs further investigation but it is anticipated that the therapeutic value of the active drug may be increased.

## Supplementary Files

Supplementary File 1
