# Supplementary material for: Synthesis of Enantiopure Reversed Structured Ether Lipids of the 1-O-Alkyl-sn-2,3-diacylglycerol Type
Source: Mar Drugs. 2015 Jan 7;13(1):173–201. doi: 10.3390/md13010173 (PMC4306931; doi:10.3390/md13010173)
Supplement: Supplementary File 1 [file marinedrugs-13-00173-s001.pdf]

Supplementary Information

Contents	Page Numbers
^{1}H NMR (400 MHz, $CDCl_3$) of compound **4a**	**S2**
^{13}C NMR (100 MHz, $CDCl_3$) of compound **4a**	**S3**
Expanded ^{1}H NMR of compound **4a**	**S4–S7**
^{1}H NMR (400 MHz, $CDCl_3$) of compound **5b**	**S8**
^{13}C NMR (100 MHz, $CDCl_3$) of compound **5b**	**S9**
Expanded ^{1}H NMR of compound **5b**	**S10–S13**
^{1}H NMR (400 MHz, $CDCl_3$) of compound **6c**	**S14**
^{13}C NMR (100 MHz, $CDCl_3$) of compound **6c**	**S15**
^{1}H NMR (400 MHz, $CDCl_3$) of compound **7d**	**S16**
^{13}C NMR (100 MHz, $CDCl_3$) of compound **7d**	**S17**
^{1}H NMR (400 MHz, $CDCl_3$) of compound **8e**	**S18**
^{13}C NMR (100 MHz, $CDCl_3$) of compound **8e**	**S19**
^{1}H NMR (400 MHz, $CDCl_3$) of compound **9f**	**S20**
^{13}C NMR (100 MHz, $CDCl_3$) of compound **9f**	**S21**

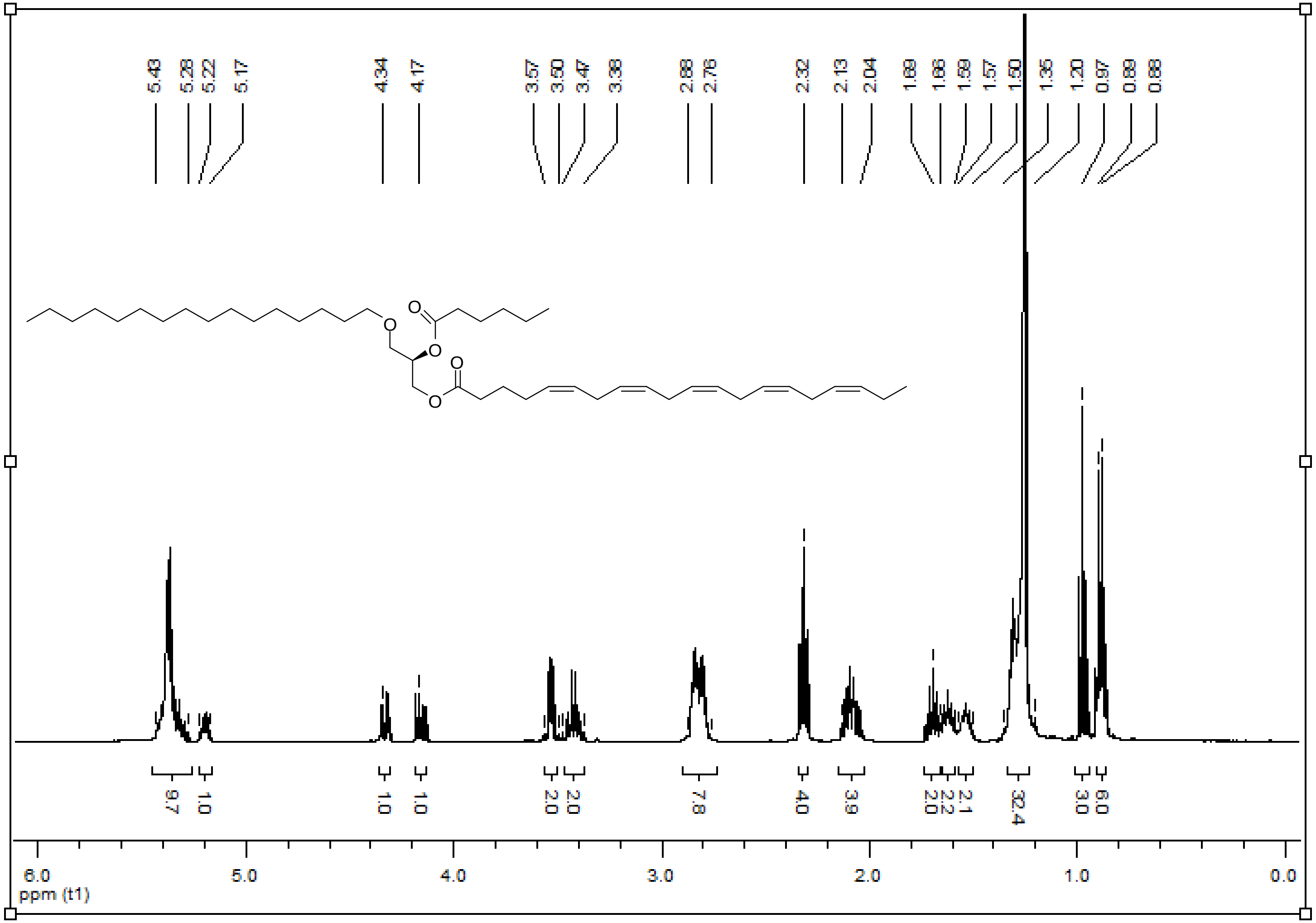

Figure S1. ^{1}H-NMR ($CDCl_3$, 400 MHz) of compound **4a**.

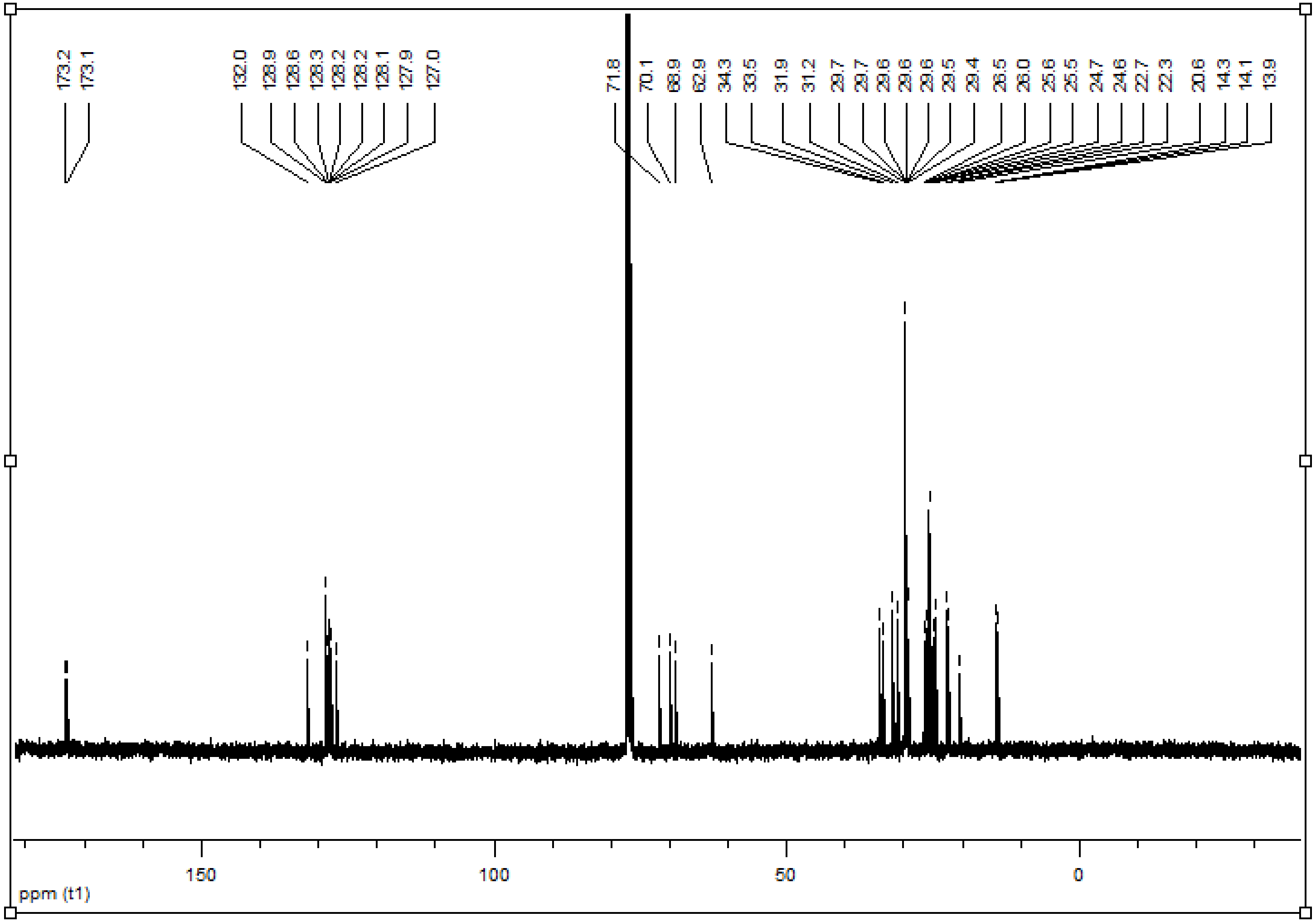

Figure S2. ^{13}C-NMR ($CDCl_3$, 100 MHz) of compound **4a**.

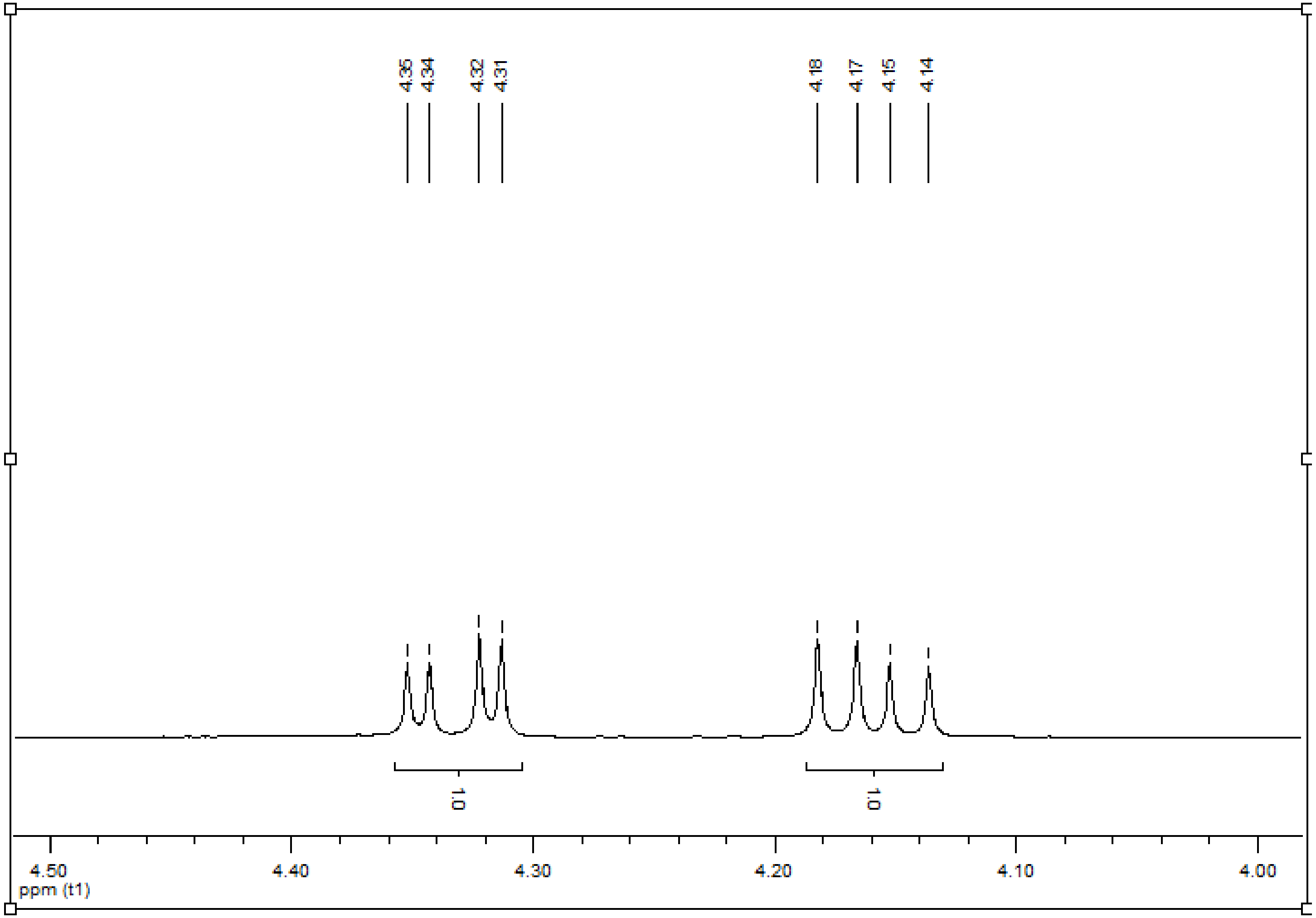

Figure S3. *Cont.*

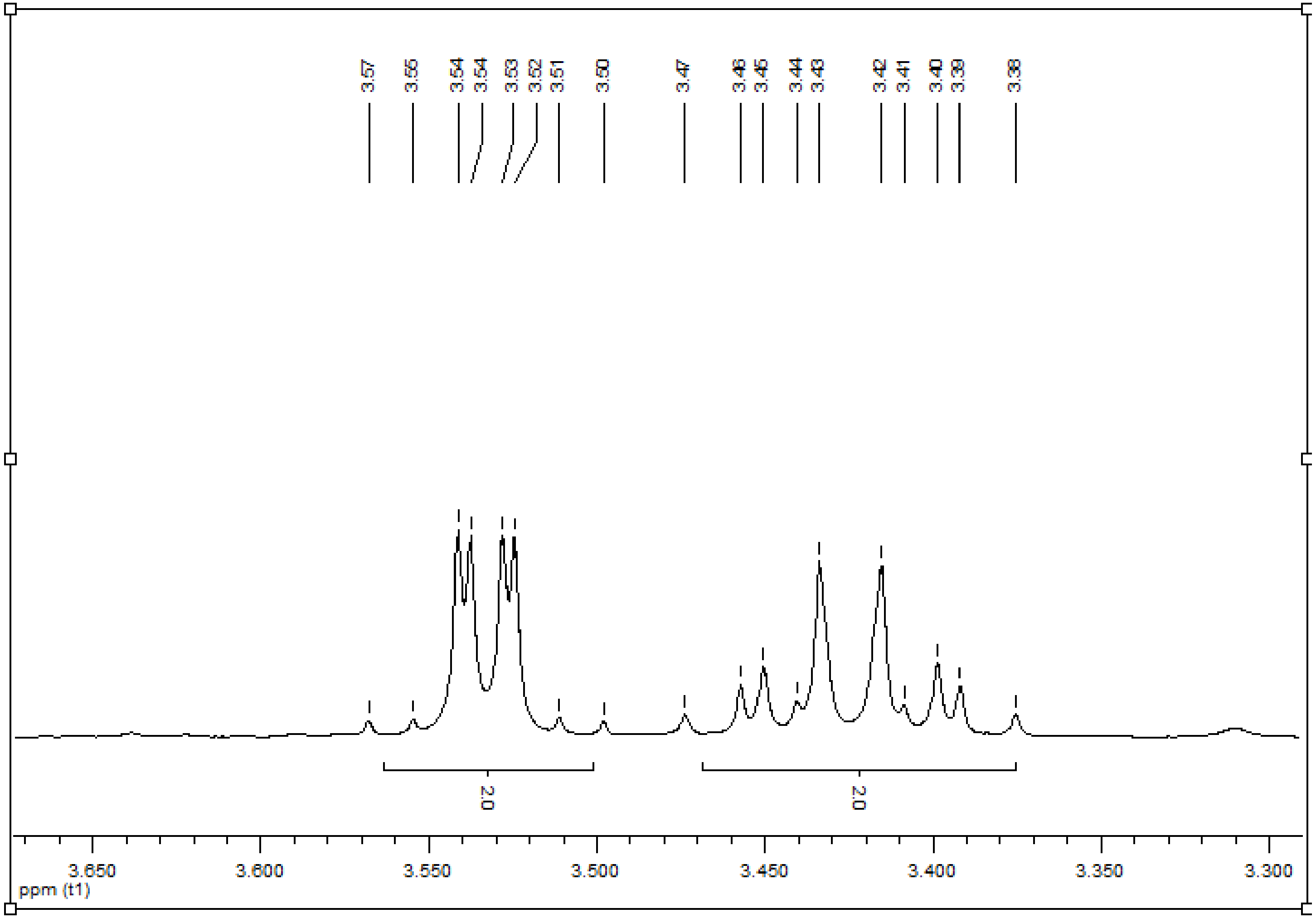

Figure S3. *Cont.*

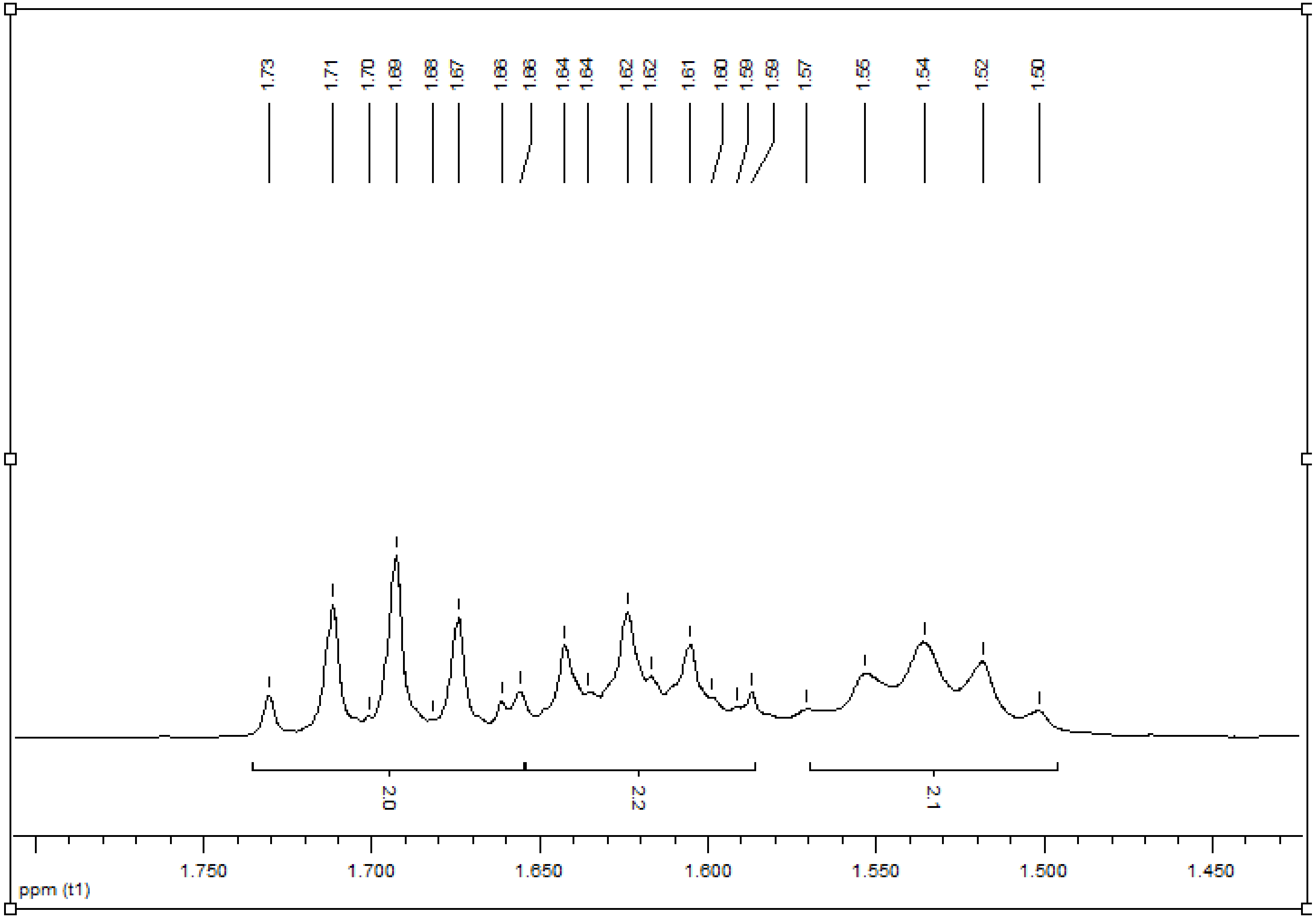

Figure S3. *Cont.*

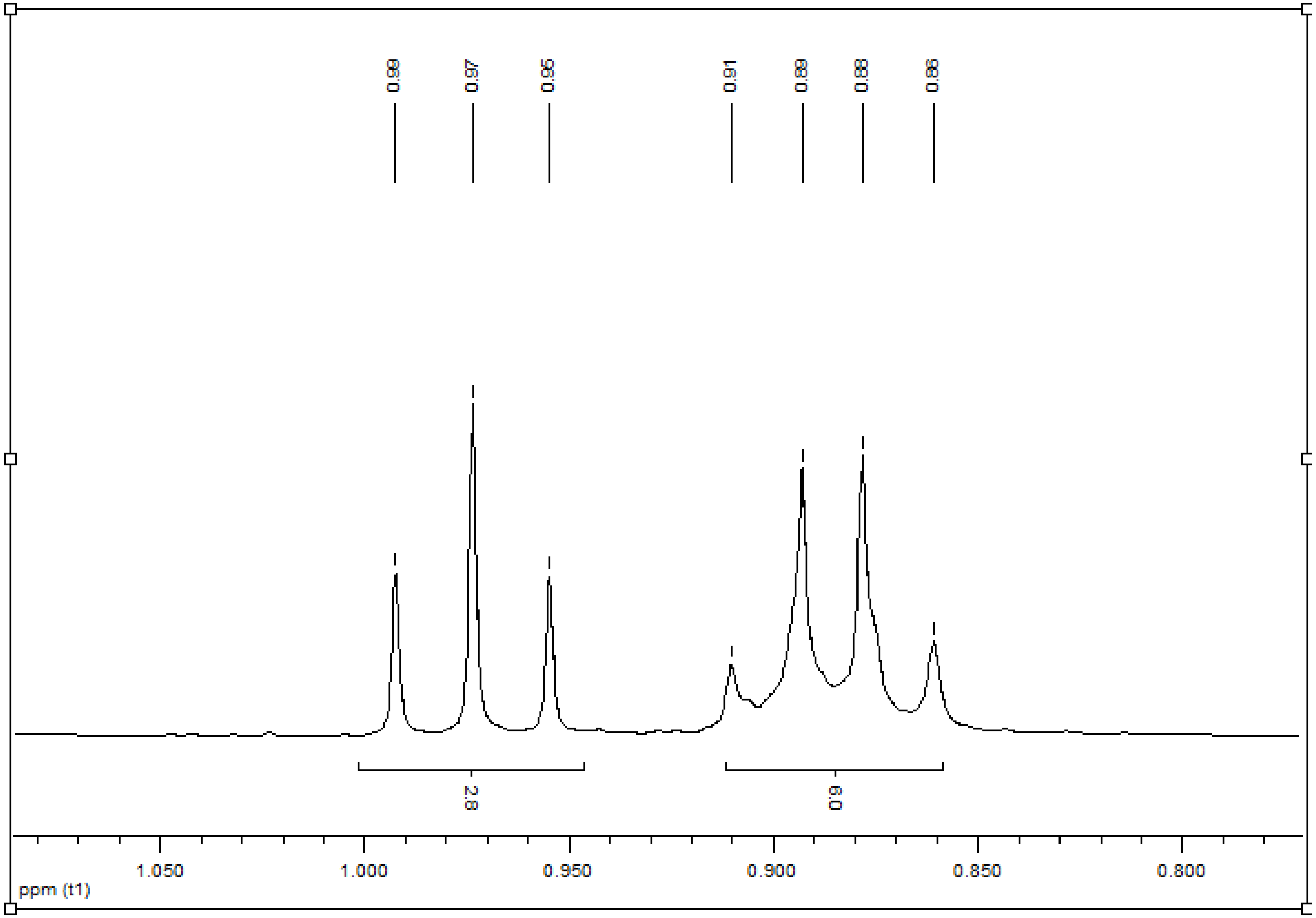

Figure S3. Expanded ^{1}H-NMR of compound **4a**.

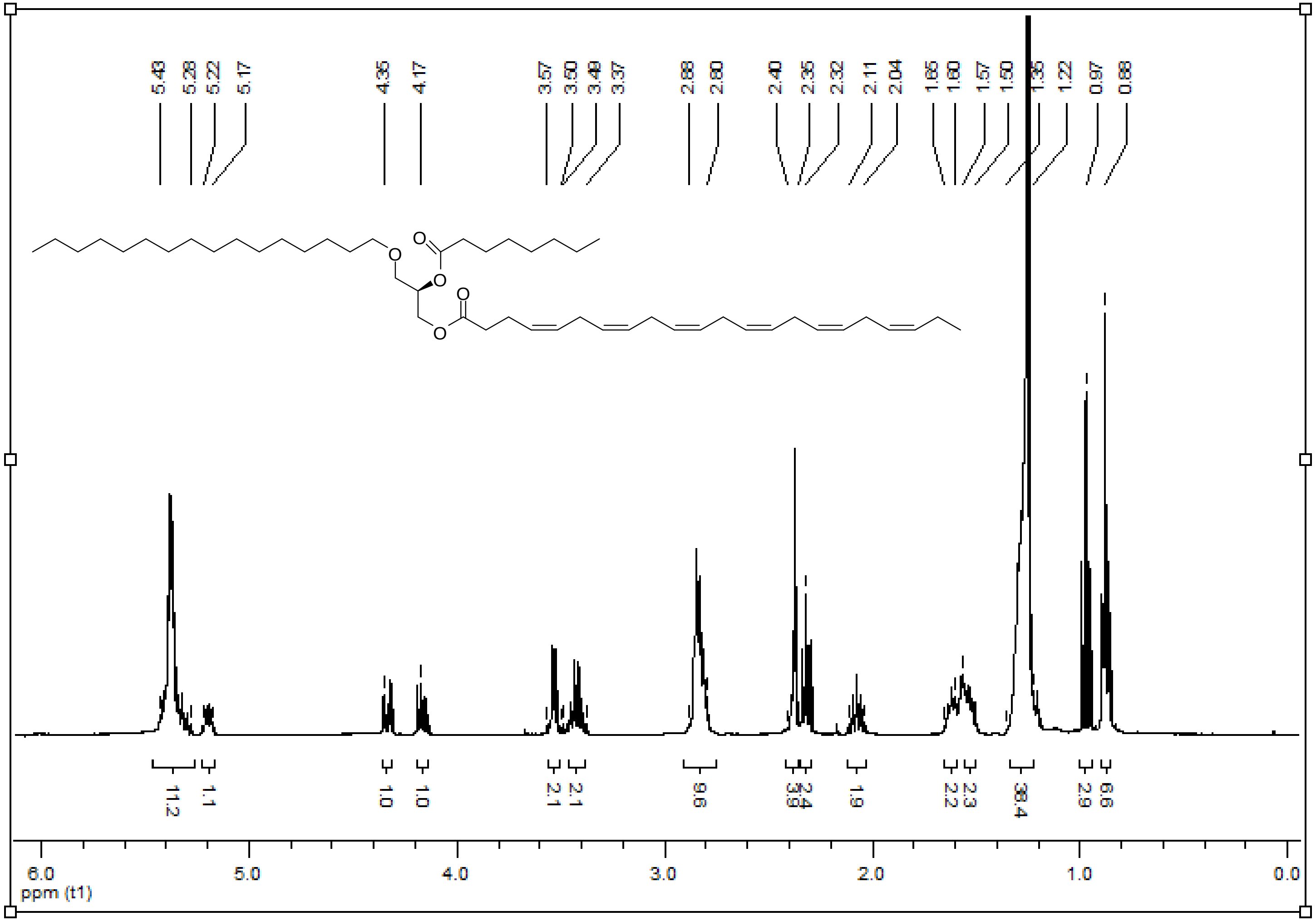

Figure S4. ^{1}H-NMR ($CDCl_3$, 400 MHz) of compound **5b**.

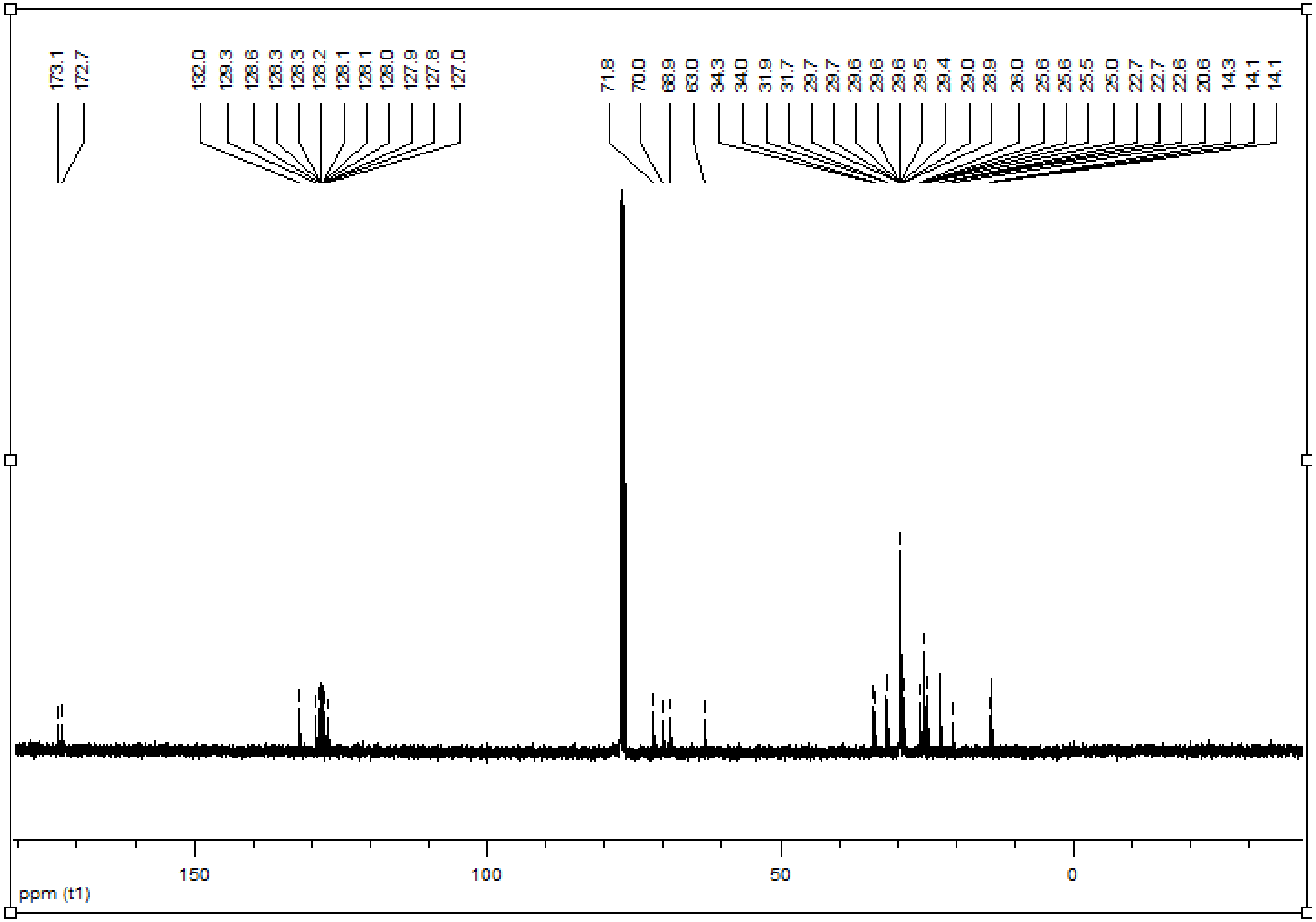

Figure S5. ^{13}C-NMR ($CDCl_3$, 100 MHz) of compound **5b**.

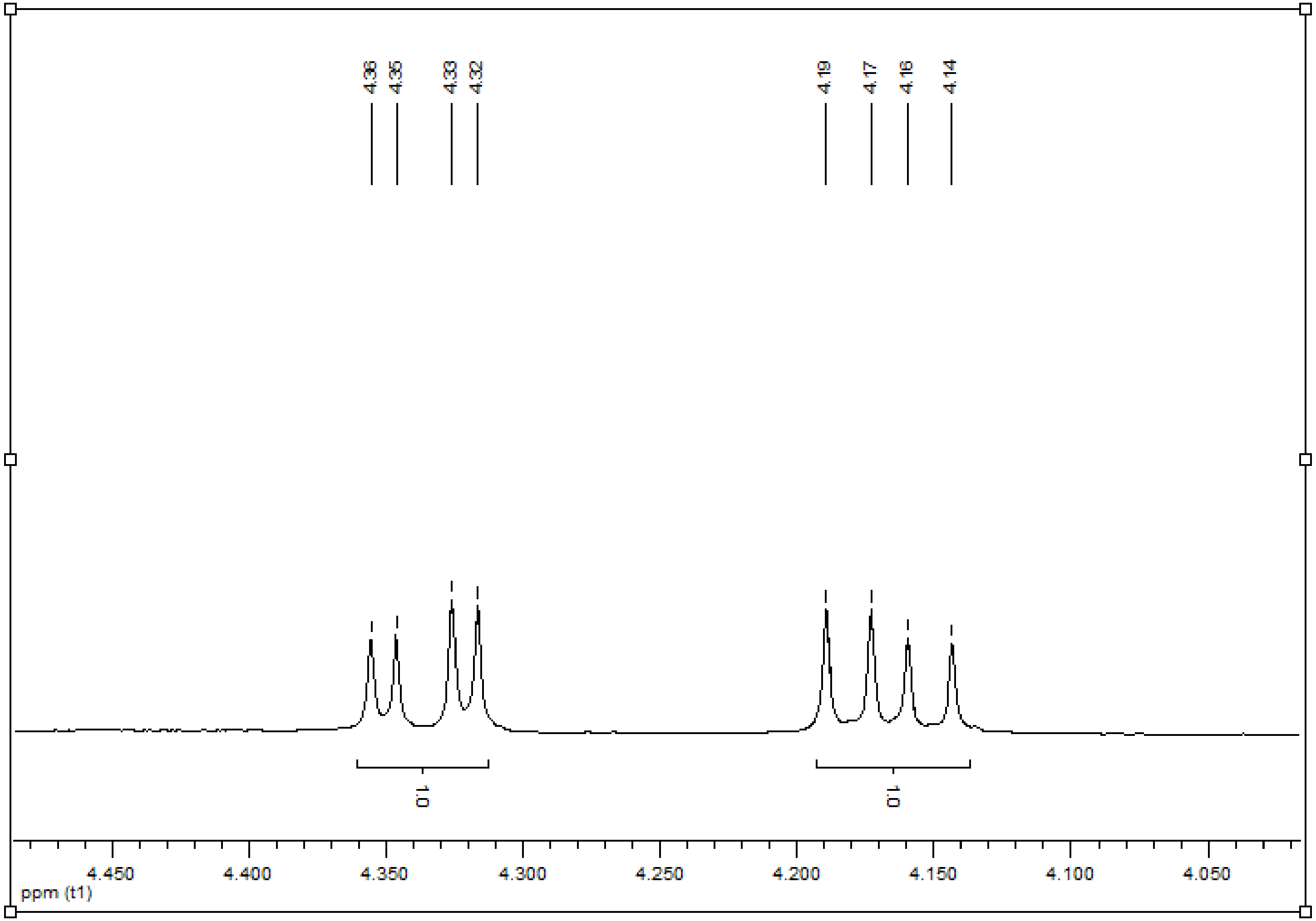

Figure S6. *Cont.*

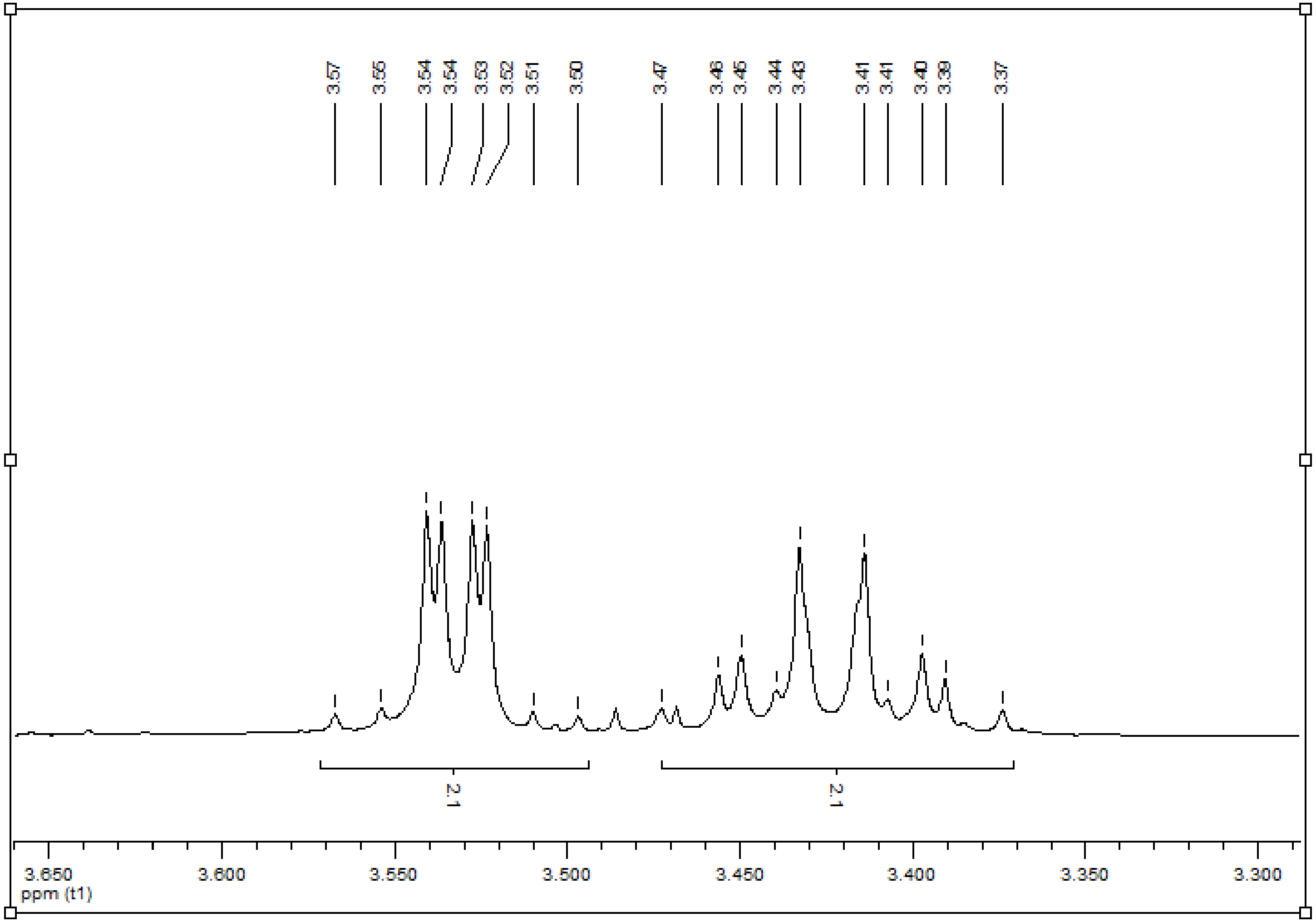

Figure S6. *Cont.*

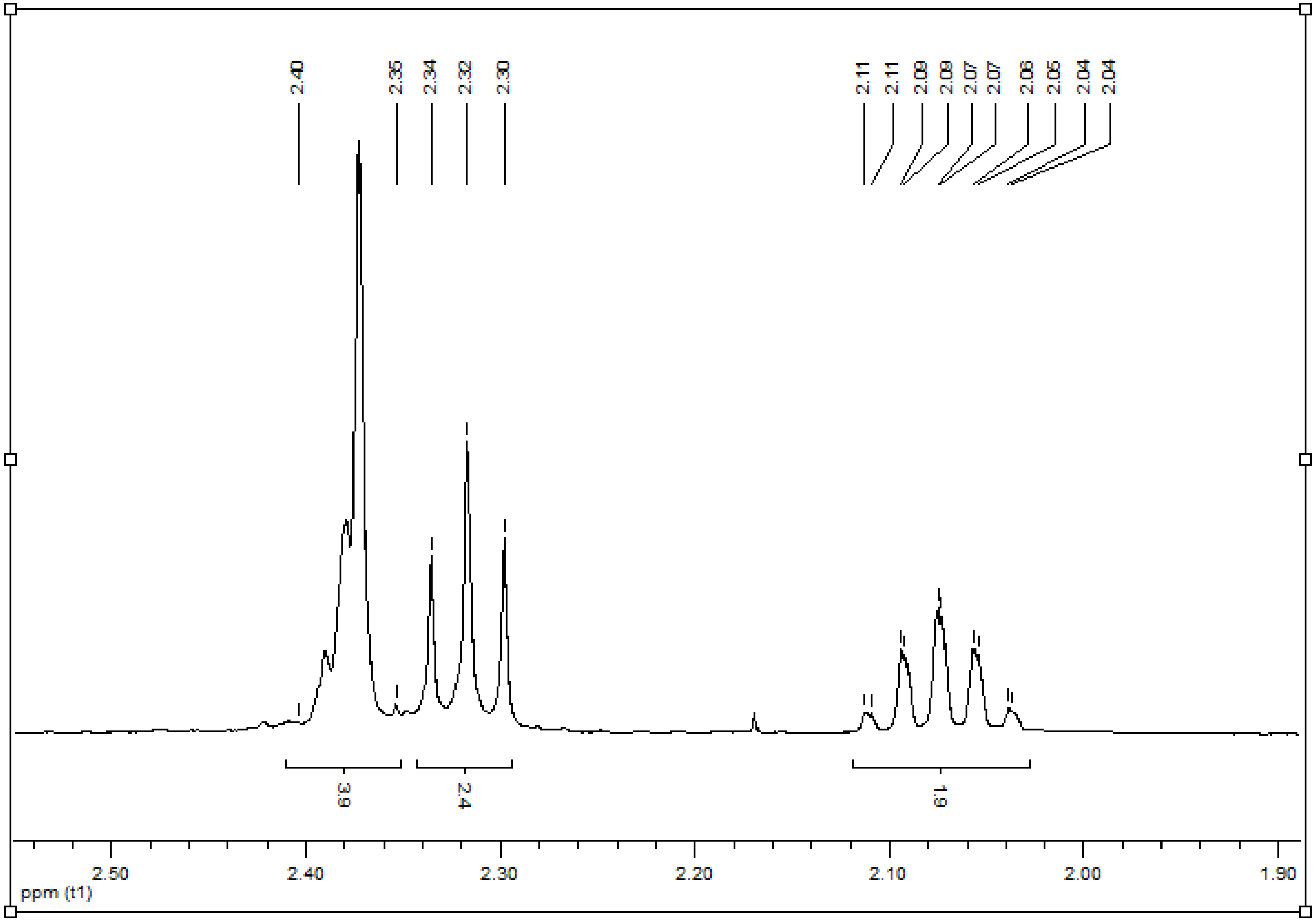

Figure S6. *Cont.*

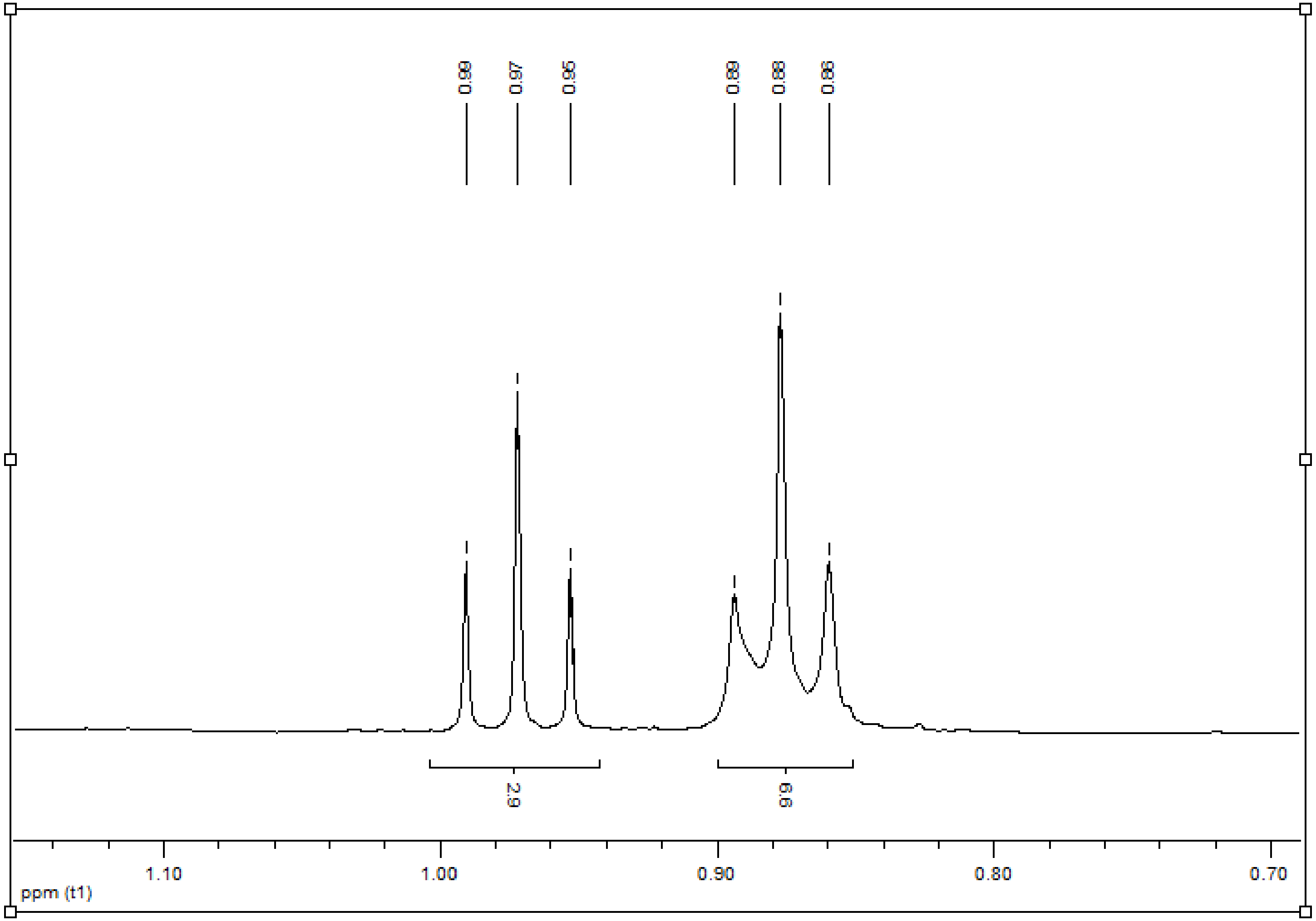

Figure S6. Expanded ^{1}H-NMR of compound **5b**.

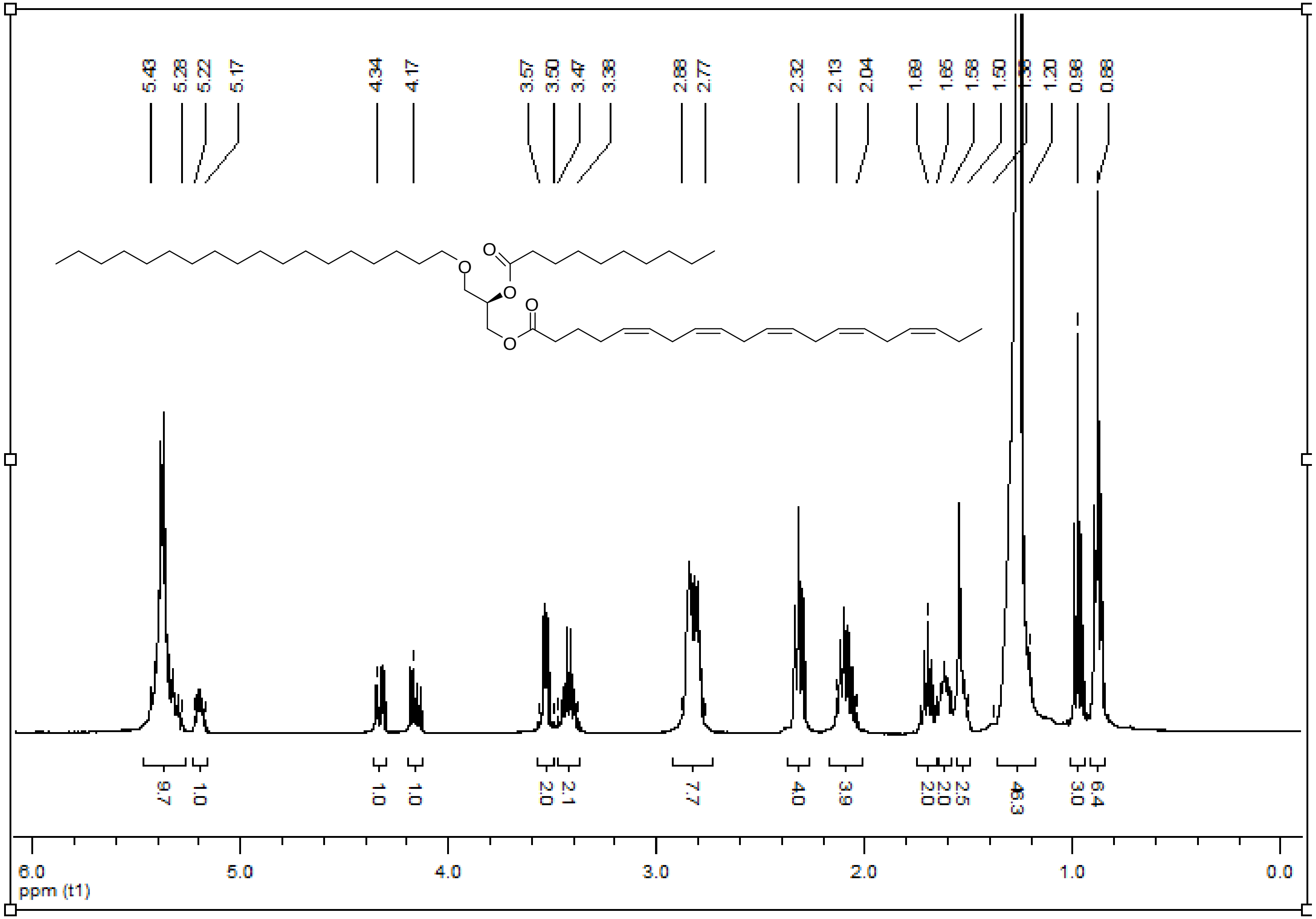

Figure S7. ^{1}H-NMR ($CDCl_3$, 400 MHz) of compound **6c**.

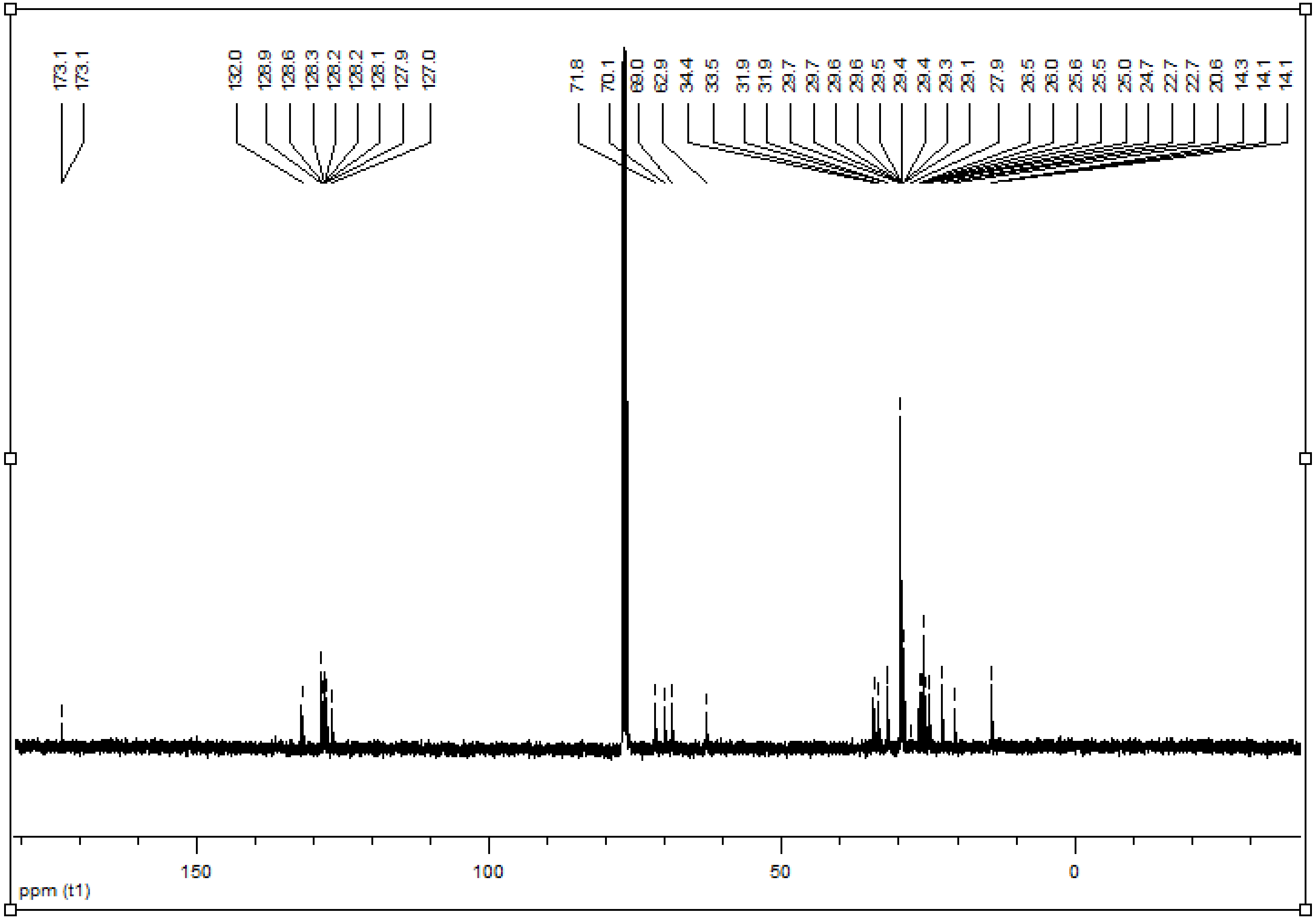

Figure S8. ^{13}C-NMR ($CDCl_3$, 100 MHz) of compound **6c**.

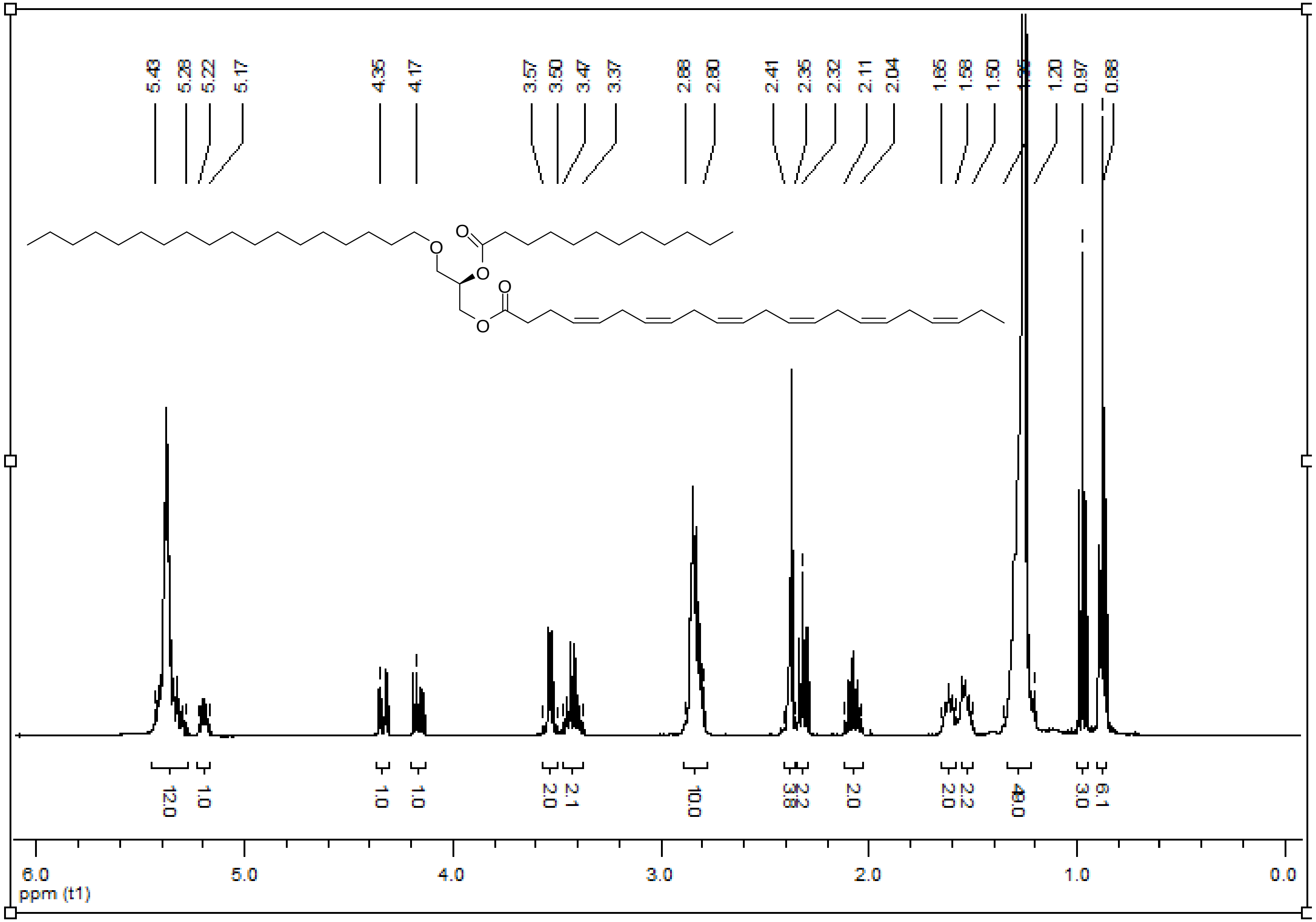

Figure S9. ^{1}H-NMR ($CDCl_3$, 400 MHz) of compound **7d**.

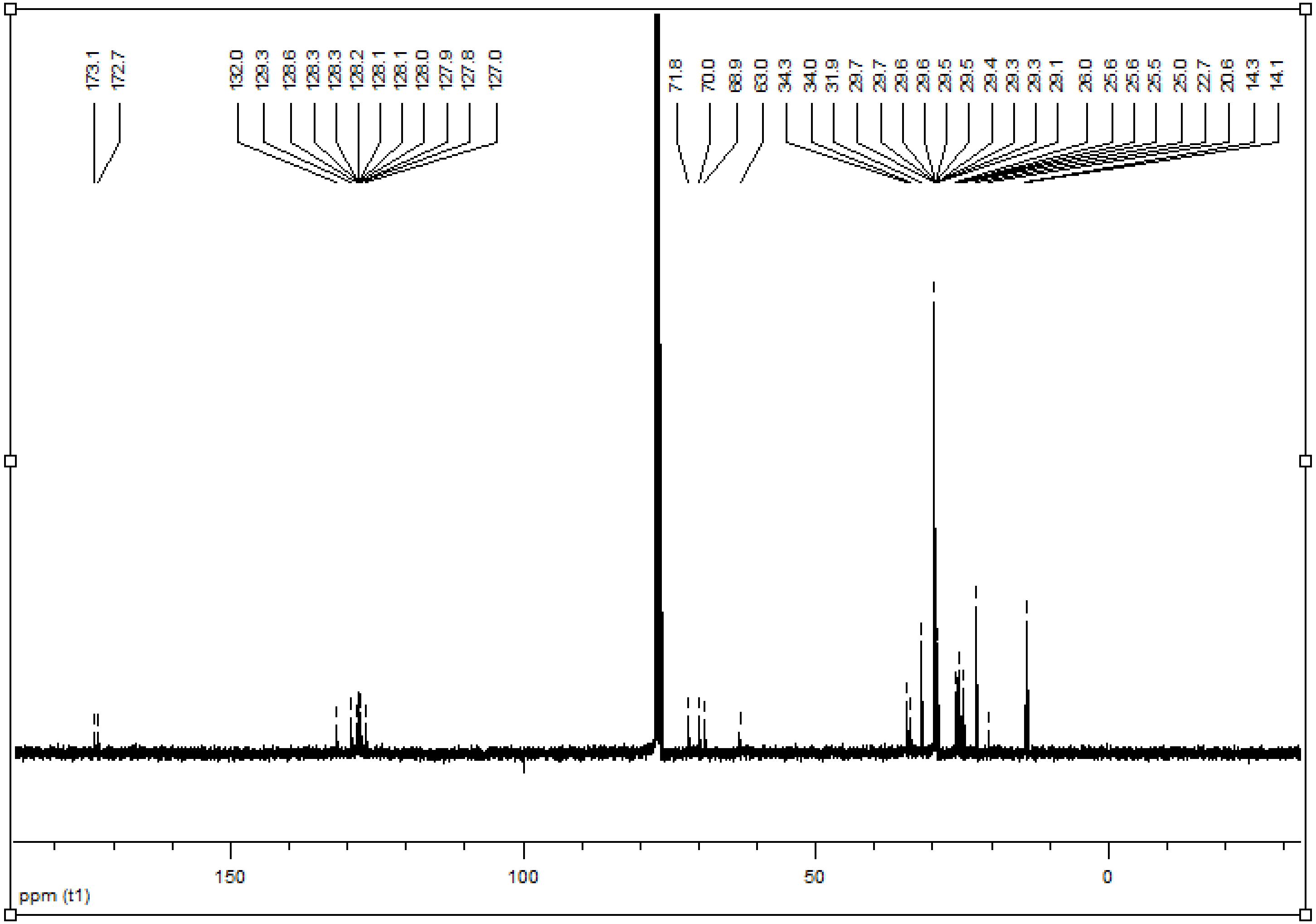

Figure S10. ^{13}C-NMR ($CDCl_3$, 100 MHz) of compound **7d**.

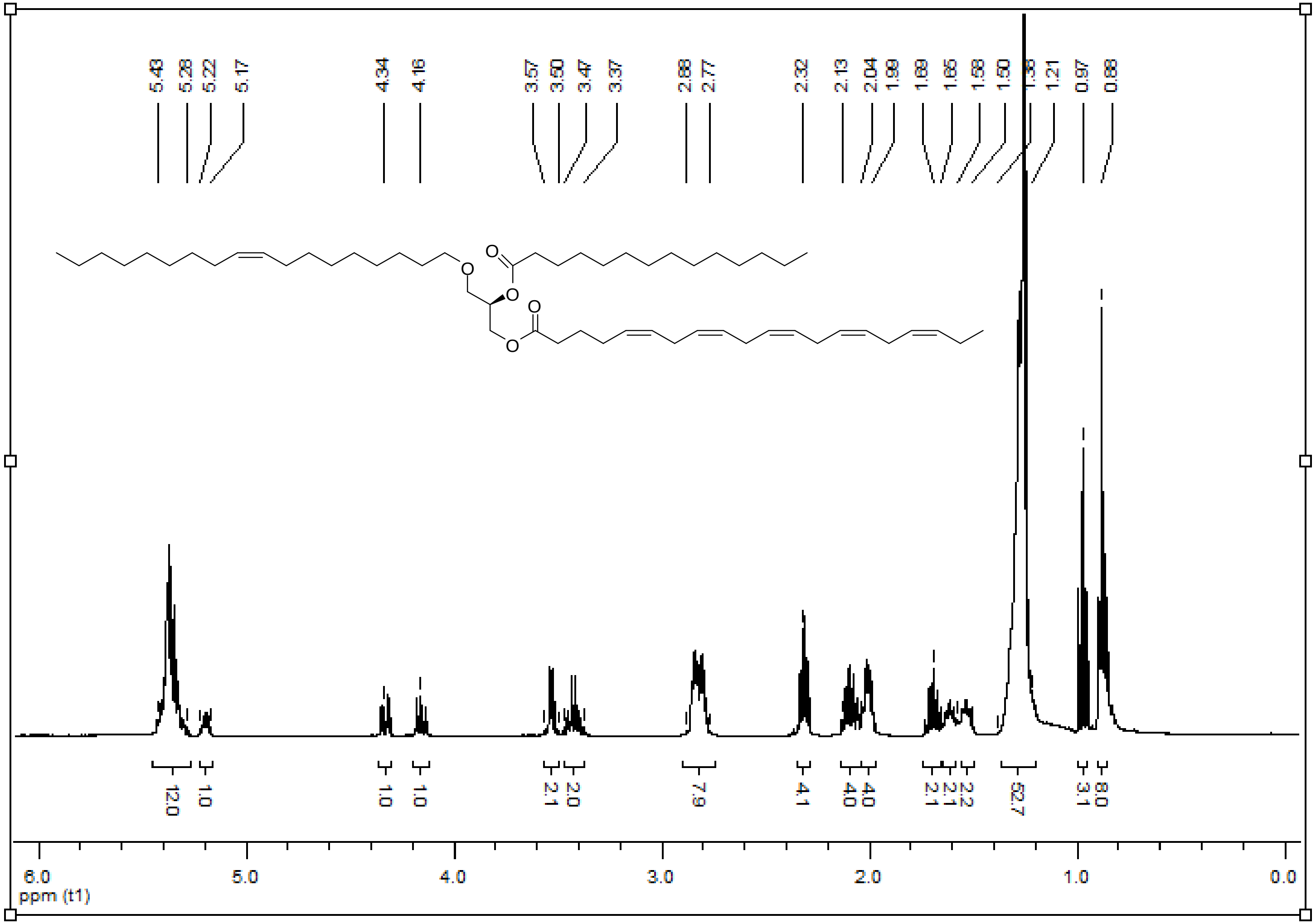

Figure S11. ^{1}H-NMR ($CDCl_3$, 400 MHz) of compound **8e**.

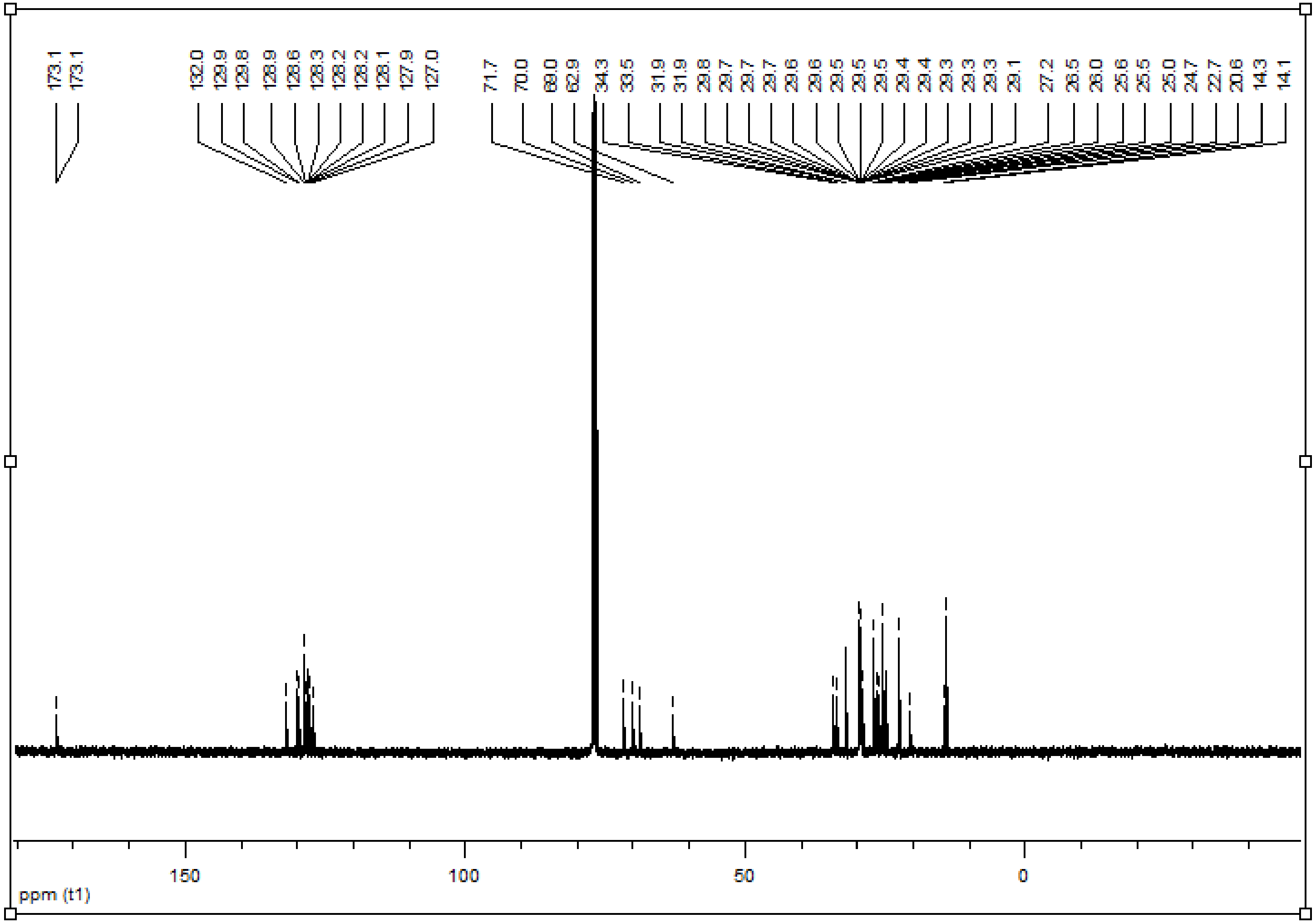

Figure S12. ^{13}C-NMR ($CDCl_3$, 100 MHz) of compound **8e**.

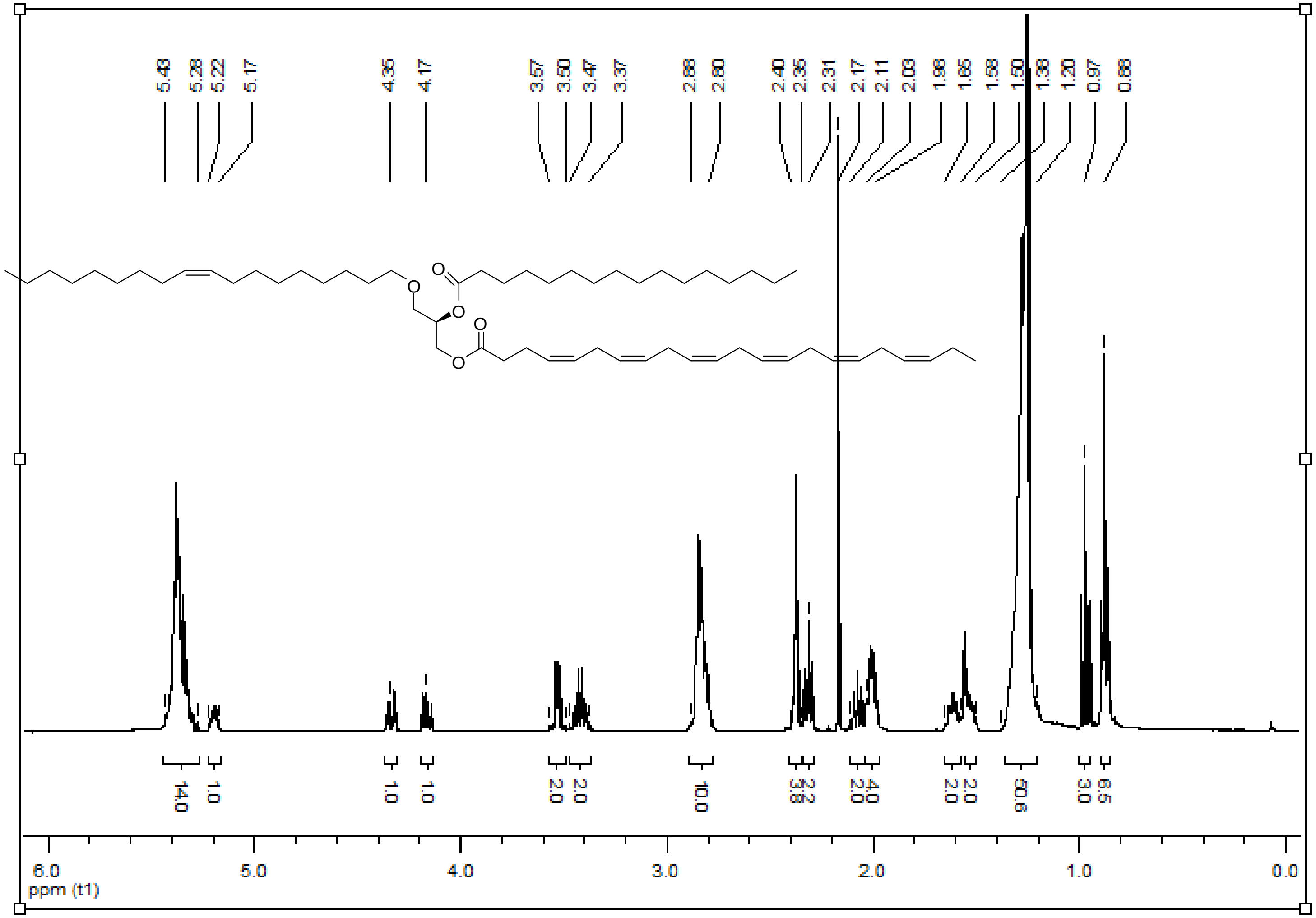

Figure S13. ^{1}H-NMR ($CDCl_3$, 400 MHz) of compound **9f**.

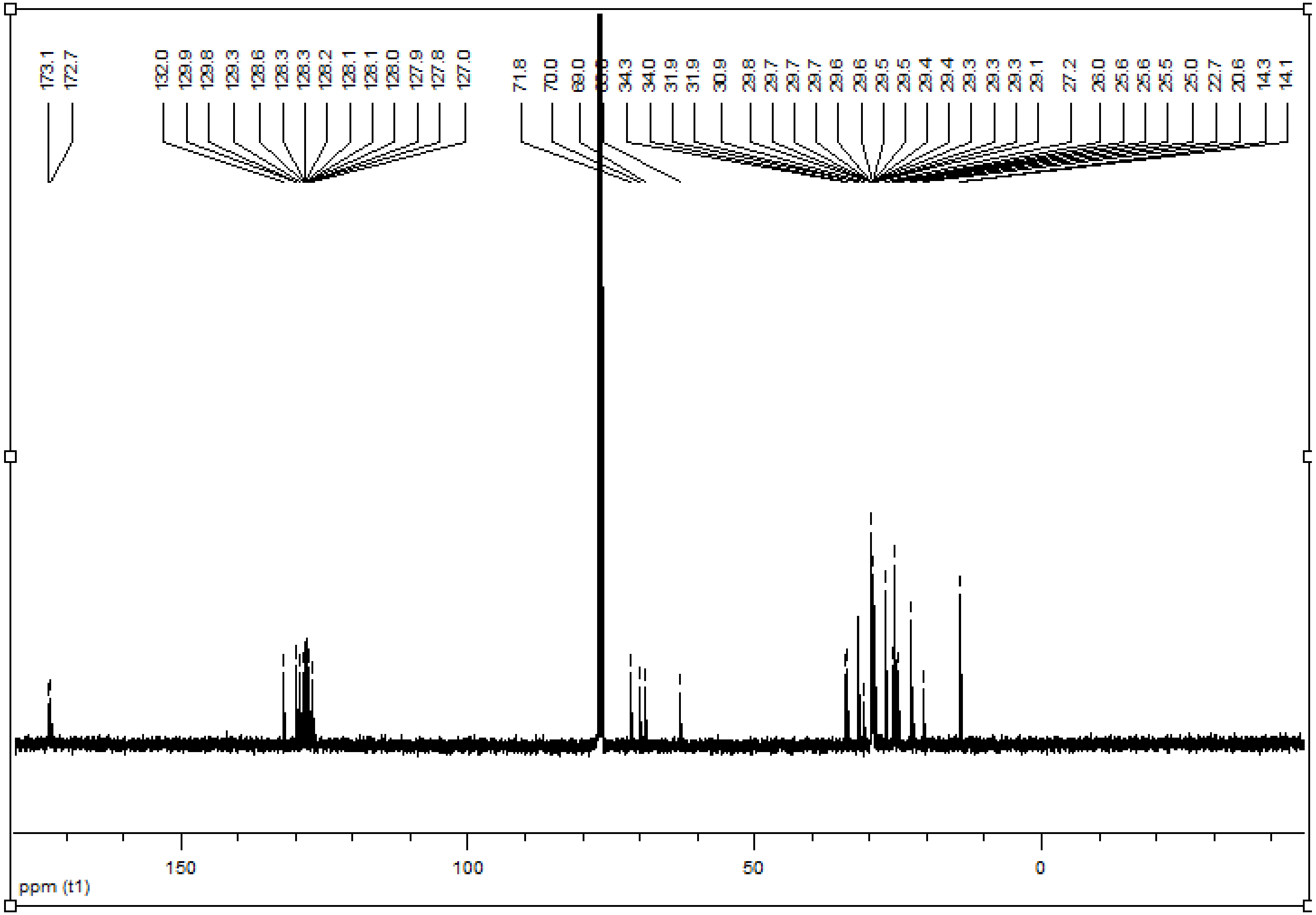

Figure S14. ^{13}C-NMR ($CDCl_3$, 100 MHz) of compound **9f**.

© 2015 by the authors; licensee MDPI, Basel, Switzerland. This article is an open access article distributed under the terms and conditions of the Creative Commons Attribution license (http://creativecommons.org/licenses/by/4.0/).
